# Mapping the distributions of blood-sucking mites and mite-borne agents in China: a modeling study

**DOI:** 10.1186/s40249-022-00966-0

**Published:** 2022-04-09

**Authors:** Tao Wang, Fanfei Meng, Tianle Che, Jinjin Chen, Haiyang Zhang, Yang Ji, Zhengwei Fan, Guoping Zhao, Wenhui Zhang, Baogui Jiang, Qiang Xu, Chenlong Lv, Taoxing Shi, Shiman Ruan, Lanzheng Liu, Wei Liu, Yang Yang, Liqun Fang

**Affiliations:** 1 State Key Laboratory of Pathogen and Biosecurity, Beijing Institute of Microbiology and Epidemiology, 20 Dong-Da Street, Fengtai District, Beijing, 100071 People’s Republic of China; 2 Jinan Center for Disease Control and Prevention, Jinan, 250021 People’s Republic of China; 3 Department of Biostatistics, College of Public Health and Health Professions and Emerging Pathogens Institute, University of Florida, Gainesville, FL USA

**Keywords:** Mite, Mite-borne pathogen, Mite-borne disease, Distribution, Risk determinant, China

## Abstract

**Background:**

Emerging mite-borne pathogens and associated disease burdens in recent decades are raising serious public health concerns, yet their distributions and ecology remain under-investigated. We aim to describe the geographical distributions of blood-sucking mites and mite-borne agents and to assess their ecological niches in China.

**Methods:**

We mapped 549 species of blood-sucking mites belonging to 100 genera at the county level and eight mite-associated agents detected from 36 species of blood-sucking mites in China during 1978–2020. Impacts of climatic and environmental factors on the ecology of 21 predominant vector mites and a leading pathogen, *Orientia tsutsugamushi*, were assessed using boosted regression tree (BRT) models, and model-predicted risks were mapped. We also estimated the model-predicted number, area and population size of affected counties for each of the 21 mite species in China.

**Results:**

*Laelaps echidninus* is the leading mite species that potentially affects 744 million people, followed by *La. jettmari* (517 million) and *Eulaelaps stabularis* (452 million). *Leptotrombidium scutellare* is the mite species harboring the highest variety of mite-borne agents including four *Rickettsia* species and two viruses, followed by *Eu. stabularis* (2 agents), *L. palpale* (2) and *La. echidninus* (2). The top two agents that parasitize the largest number of mite species are *O. tsutsugamushi* (28 species) and hantavirus (8). Mammalian richness, annual mean temperature and precipitation of the driest quarter jointly determine the ecology of the mites, forming four clusters of major mite species with distinct geographic distributions. High-risk areas of *O. tsutsugamushi* are mainly distributed in southern and eastern coastal provinces where 71.5 million people live.

**Conclusions:**

Ecological niches of major mite species and mite-borne pathogens are much more extensive than what have been observed, necessitating expansion of current filed surveillance.

**Graphic Abstract:**

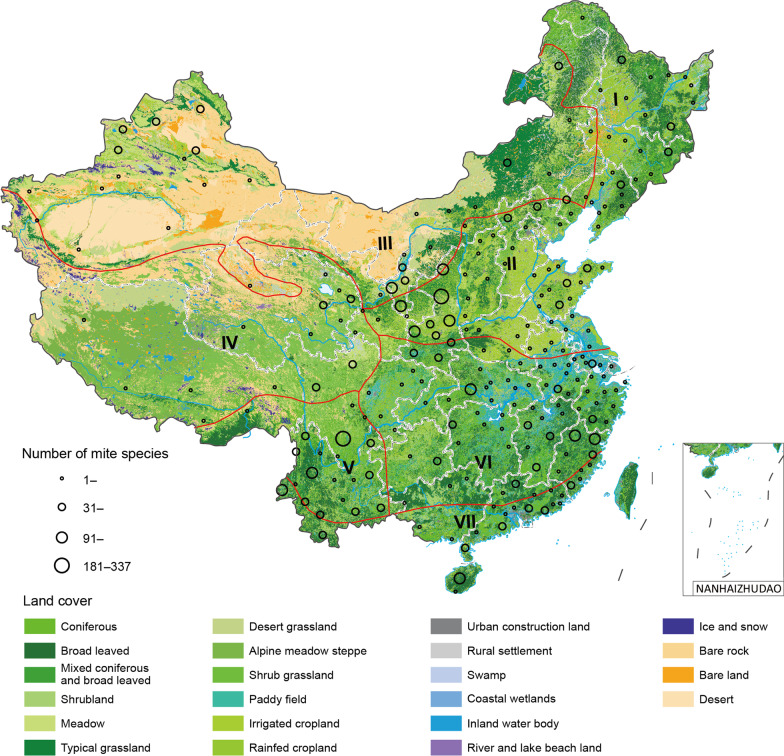

**Supplementary Information:**

The online version contains supplementary material available at 10.1186/s40249-022-00966-0.

## Background

Vector-borne infections (VBI), which are defined as infectious diseases transmitted through the bites of blood-feeding arthropods (including but not limited to ticks, mosquitoes, mites, fleas, sandflies, etc.) [1], constitute a significant proportion of the global infectious disease burden. Ticks and mosquitoes are recognized as two major vectors in the transmission of pathogens to human and animals worldwide [2]. Research has been prioritized for VBIs transmitted by mosquitoes or ticks, e.g., malaria, dengue, chikungunya, Zika, lymphatic filariasis, Lyme disease, tick-borne spotted fever, severe fever thrombocytopenia syndrome, and tick-borne encephalitis, while mite-borne infections were largely neglected. Blood-sucking mites play an important role in transmitting various pathogens to humans [3–5], including *O. tsutsugamushi* [6], hantaviruses [7] and even *Yersinia pestis* [8], and some of these pathogens are distributed globally. Moreover, mites can parasitize a great variety of vertebrates including livestock, birds, rodents, and other wild life [9–11]. As a competent vector for transmitting various pathogens across multiple host species, mites are becoming a serious concern in agriculture, veterinary medicine and public health [12, 13].

Currently, more than 55,000 species of mites are known to exist in the world with complex habitation environment and feeding activities, afflicting human and animal health in different ways [14]. Some of the domestic mite species such as house dust mites are found in indoor environments of warm or tropical regions and cause allergic disorders rather than infection via blood sucking. Among parasitic blood-sucking mites with medical implications [15–17], species or sub-species of gamasid mites, chigger mites and oribatid mites are considered as the most common mites that transmit pathogens [18].

Notably, the increasing incidence and spatial spread of mite-borne diseases in human beings have been related to the ongoing geographic expansion of mite species, possibly driven by climatic and environmental changes, with scrub typhus as the most prominent example [19, 20]. Scrub typhus has been historically considered native to the “Tsutsugamushi Triangle” bounded by Japan in the east, Pakistan in the west, Russia in the north and Australia in the south. However, recent reports of human cases/outbreaks went beyond the previously recognized endemic region, e.g., in Kenya [21] and Chile [22, 23]. The potentially changing ecology of mites is calling for studies to update the diversity and distribution of mite species, in order to better understand the spatial distributions and levels of the risks of VBIs. Here we conduct an up-to-date review on the distributions of blood-sucking mites and mite-associated agents and build predictive models, designed to describe their most recent geographical distribution and to assess their ecological niches in China.

## Methods

### Data on blood-sucking mites and mite-associated agents

A comprehensive database of all blood-sucking mites and mite-associated agents that have ever been reported in China (the database is publicly available in Additional files 1 and 2 [24]) was assembled from two sources: (1) literature search in public databases in either Chinese or English (Additional file 3: Fig. S1), (2) collection of mite-related records from two books in Chinese: *China Economic Entomology* compiled by Institute of Zoology, Chinese Academy of Sciences [25]; and *Atlas of Epidemiology of Natural Focus Diseases in China* complied by Beijing Institute of Microbiology and Epidemiology [26] (Additional file 3: Table S1, S2). For the literature review, we searched four major electronic databases, China National Knowledge Infrastructure (CNKI) (http://www.cnki.net/), Wan Fang (http://www.wanfangdata.com.cn/), VIP (http://www.cqvip.com/), and PubMed (https://pubmed.ncbi.nlm.nih.gov/) for studies published between 1978 and 2020, using the following keywords: (“mite” or “mites”) and “China” (Additional file 3: Fig. S1). Studies were eligible if they described identification of mites and/or laboratory detection of mite-associated agents. At the same time, we excluded studies that were: (I) not related to detection result of mite species and mite-associated agents; (II) testing insecticides, detection tools, drugs or vaccines; or (III) focusing on molecular research of mites and agents. Details on the inclusion and exclusion criteria can be found in Additional file 3: Table S3. Each article was screened by two team members independently to collect the following information using a standard form: study date, study location, spatial resolution of each record, blood-sucking mite species identified, laboratory methods and detection results for mite-associated agents. Data were checked carefully to ensure that standard extraction criteria were met, similar to the approach used in Herrera et al. [27]*.*

The county or the finest available spatial resolution were extracted from each reported of blood-sucking mites or mite-associated agents. If a blood-sucking mite specie or a mite-associated agent was reported more than once in the same county during the study period (e.g., found by different study groups at different times or locations within the same county), only one record was counted for our analyses. If more than one pathogen were determined from the same mite, a record was created for each pathogen in our database. In addition, we collected 51 socioenvironmental and ecoclimatic factors that are potentially associated with the ecology of blood-sucking mite species and mite-associated agents (Additional file 3: Materials and Methods, Tables S4, S5). The spatial resolutions of the original socioenvironmental and ecoclimatic data are at the county or finer levels and are listed in Table S5. Data at finer resolutions were summarized at the county level to be used in the ecological models.

### Ecological modeling of distribution of mites

Boosted regression tree (BRT) models were constructed to predict the distribution of 21 dominant blood-sucking mite species at the county level. Briefly, a case-control study design was applied to build predictive models, where counties having at least one record of occurrence serve as “cases” and those surveyed but yielding no evidence of occurrence serve as “controls” [28]. The remaining counties where either no survey was conducted or surveys did not lead to conclusive findings were excluded from model-building but were included for risk mapping. To counterbalance the potential sampling bias of surveyed counties, we estimated the sampling probabilities of all counties by building a logistic regression with mite-survey history at the county level (1: yes, 0: no) as the response and ecoclimatic and socioenvironmental variables as predictors. Predictors were chosen using a backward procedure at the significance level of 0.05. The reciprocals of predicted sampling probabilities of all surveyed counties were used as weights (rescaled to have a mean 1) in the BRT models [29–31]. This weighting scheme creates a balanced pseudo-sample population when there is a sampling bias related to the outcome of interest [29], and it has been used in several ecological modeling studies [32–34]. In provincial-level administrative divisions (PLADs) where investigations of mites were scarce, e.g., in Guangxi, counties have been mostly assigned higher weights as they were under-sampled (Additional file 3: Fig. S2).

As eco-climatic predictors are often highly correlated with each other, we performed a clustering analysis on these predictors based on their pairwise correlation coefficients using the package “NbClust” of the R 4.0.3 software (Lucent Technologies, Jasmine Mountain, USA). Specifically, a binary distance matrix was formed with the distance between any pair of eco-climatic variables being 0 if the absolute value of correlation coefficient is bigger than 0.8 and 1 otherwise. The best number of clusters was chosen by the Krzanowski and Lai index [35]. This clustering analysis found eight clusters of the ecoclimatic (Additional file 3: Table S4). A continuous distance matrix where the distance is one minus the absolute value of correlation coefficient also identified the same clusters. Only one predictor from each cluster was used for model-fitting (Additional file 3: Table S4). For each BRT model, a total of 40 variables including 30 environmental factors, 8 ecoclimatic factors, 1 economic factor and 1 demographic factor (Additional file 3: Materials and Methods, Table S4, S5) were used as predictors.

The BRT models were fitted with a tree complexity of 5, a learning rate of 0.005 and a bagging fraction of 75%, based on their satisfactory performance in our previous researches [36, 37]. The output of each BRT model consists of both predicted probabilities of occurrence and relative contributions (or influences) of predictors. A training set with 75% of data points was randomly sampled without replacement, and the remaining 25% served as a test set. A BRT model was built using the training set, and then applied to the test set for validation if needed. The model-fitted risks were plotted on each predictor. Furthermore, receiver-operating characteristic (ROC) curves and areas under the curve (AUC) were produced to assess the predictive power of the models. Considering the possibility of false negative and false positive counties in the observed data, we also calculated partial area AUC with a tolerance level of 0.2 for omission error as described in Peterson’s study [38].

Model-predicted probabilities of occurrence were mapped to demonstrate the risk distribution for each of the 21 blood-sucking mites. We chose maximizes sensitivity + specificity along the ROC curve as a cut-off value for each final BRT model [39, 40]. Counties with predicted probabilities above the cut-off value for a given model were considered as having a high risk of harboring the corresponding blood-sucking mite species. We further estimated the sizes of populations in high-risk regions. For each mite species, the number, area and population size of model-predicted high-risk counties were compared to the quantities of counties with observed occurrence (Table 1). All statistical analyses were performed using the dismo and gbm packages of the R 4.0.3 software (Lucent Technologies, Jasmine Mountain, USA).

**Table 1:** The average testing areas-under-curve (AUC) of the BRT models at the county level and model-predicted numbers, areas and population sizes of affected counties for the 21 most prevalent mite species in China

Mite species	Average AUC (5 ‒95% percentiles)	Predicted/observed (relative difference%)		
		No. of counties	Area (10,000 km^2^)	Population size (million)
*Ha. glasgowf*^*abc*^	0.78 (0.74, 0.82)	897/259 (246.3)	456.8/189.8 (140.7)	415.6/115.2 (260.8)
*La. echidninus*^*abc*^	0.78 (0.73, 0.83)	1,493/200 (646.5)	295.8/58.0 (410.0)	744.1/91.3 (715.0)
*Eu. stabularis*^*abc*^	0.77 (0.73, 0.82)	861/188 (358.0)	208.4/76.4 (172.8)	451.7/93.9 (381.0)
*La. jettmari*^*abc*^	0.81 (0.77, 0.87)	1,046/185 (465.4)	242.1/67.3 (259.7)	516.7/91.7 (463.5)
*Hy. pavlovskii*^*b*^	0.80 (0.73, 0.85)	703/140 (402.1)	197.5/65.8 (200.2)	250.6/46.4 (440.1)
*La. nuttalli*^*ac*^	0.81 (0.76, 0.85)	745/138 (439.9)	175.7/43.8 (301.1)	320.6/64.1 (400.2)
*L. deliense*	0.92 (0.88, 0.95)	534/126 (323.8)	106.6/32.9 (224.0)	287.2/68.4 (319.9)
*Hi. sunci*	0.78 (0.72, 0.85)	495/117 (323.1)	111.6/37.5 (197.6)	213.4/47.1 (353.1)
*Or. bacoti*	0.76 (0.69, 0.83)	598/109 (448.6)	99.4/26.9 (269.5)	333.1/62.4 (433.8)
*Hy. lubrica*	0.80 (0.72, 0.86)	393/100 (293.0)	132.5/57.1 (132.0)	135.9/31.5 (331.4)
*Tr. myonysognathus*	0.85 (0.81, 0.90)	263/97 (171.1)	55.9/20.4 (174.0)	138.6/52.9 (162.0)
*L. scutellare*	0.85 (0.78, 0.90)	402/86 (367.4)	64.5/18.8 (243.1)	243.4/43.8 (455.7)
*Od. majesticus*	0.93 (0.89, 0.96)	349/75 (365.3)	49.9/13.7 (264.2)	233.6/58.1 (302.1)
*Eu. shanghaiensis*	0.86 (0.77, 0.94)	389/59 (559.3)	75.7/14.2 (433.1)	172.8/23.4 (638.5)
*L. intermedium*	0.78 (0.67, 0.89)	420/50 (740.0)	94.4/14.1 (569.5)	176.5/23.2 (660.8)
*L. yui*	0.89 (0.81, 0.95)	169/49 (244.9)	30.1/11.9 (152.9)	74.9/23.2 (222.8)
*Hi. isabellinus*	0.82 (0.70, 0.90)	238/43 (453.5)	155.2/50.4 (207.9)	55.6/12.1 (359.5)
*L. fuji*	0.89 (0.82, 0.94)	140/40 (250.0)	34.9/11.8 (195.8)	55.3/16.2 (241.4)
*L. palpale*	0.84 (0.75, 0.93)	384/39 (884.6)	44.8/8.0 (460.0)	225.6/25.2 (795.2)
*As. indica*	0.92 (0.85, 0.97)	148/37 (300.0)	36.2/10.3 (251.5)	57.3/12.6 (354.8)
*L. rubellum*	0.95 (0.90, 0.99)	62/28 (121.4)	19.0/9.9 (91.9)	20.4/8.9 (129.2)

The predicted numbers are compared with the actual observations from field surveys and the relative differences (%) are given in parentheses

^a^Top 5 mite species affecting largest numbers of counties

^b^Top 5 mite species affecting largest areas

^c^Top 5 mite species affecting largest population sizes

### Population at risk for emerging mite-associated agents

BRT models were also used to evaluate the risks and risk drivers for the presence of *O. tsutsugamushi*, the etiological pathogen for scrub typhus which is one of the reportable infectious diseases in China. The same 40 environmental and ecoclimatic variables used for modeling mite species were considered as potential predictors. In addition, the predicted occurrence probabilities of nine blood-sucking mite species, possible vectors of *O. tsutsugamushi* as reported in the literature were also included as potential predictors. All counties in the mainland of China where human cases of scrub typhus were reported during 2010‒2018 were regarded as “case” counties in the BRT model. In addition, those counties where no human case of scrub typhus was reported but the pathogen “*O. tsutsugamushi*” was detected in mites between 1978 and 2020 were also considered as “case” counties. For a county to be assigned as a “control”, the following conditions must be satisfied: (1) No human cases of the associated mite-borne diseases have been reported in the national surveillance system and no other evidence for the presence of the pathogen was found in the literature; (2) Either the primary mite vectors of *O. tsutsugamushi* for scrub typhus (including *L. deliense*, *L. fuji, L. intermedium*, *Od*. *majesticus*, *L. rubellum*, *L*. *scutellare*, *L. palpale*, *As*. *indica*, *L. yui*) were surveyed but not found, or the primary mite vectors were not surveyed and the model-predicted probability of existence of each primary vector is smaller than the cut-off that yields the best predictive performance of the ecological model for that vector (represented by the Youden’s index). The counties meeting neither the “case” nor the “control” definitions were excluded from modeling. In total, only 142 counties, about 5.0% of all counties in the mainland of China, were excluded.

## Results

### Distribution of mite species

We compiled a database comprising 6,443 unique records on geographic distributions of 549 blood-sucking mite species belonging to 100 genera, which were recorded in 759 counties (27% of all counties in the mainland of China) (Additional file 3: Materials and Methods, Fig. S3, Table S1). Seven mite genera were found in > 200 counties and were considered as the common mites in China. The most widely distributed was *Laelaps* (detected in 362 counties), followed by *Haemolaelaps* (301), *Eulaelaps* (270), *Hirstionyssus* (242), *Leptotrombidium* (243), *Haemogamasus* (217), and *Hypoaspis* (208) (Additional file 3: Fig. S4–10, Table S1). At the species level, 10 were detected in > 100 counties, with *Ha*. *glasgowf* as the most predominant (in 259 counties), followed by *La. echidninus* (200), *Eu. stabularis* (188), *La. jettmari* (188), *Hy*. *pavloskii* (147), *La*. *nuttall* (140), *L. delicense* (126), *Hi*. *sunci* (117), *Hy*. *lubrica* (117) and *Or. bacoti* (113) (Additional file 3: Table S1).

**Fig. 1:**
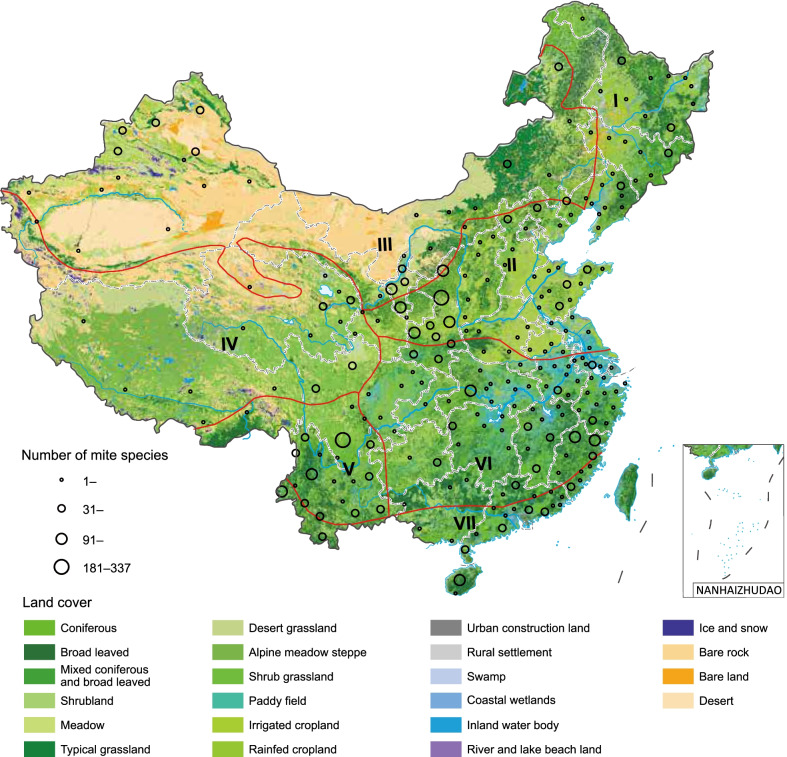
Mite species richness (circles) at the prefecture level in seven biogeographic zones in the mainland of China from 1978 to 2020. I = Northeast zone, II = North China zone, III = Inner Mongolia-Xinjiang zone, IV = Qinghai-Tibet zone, V = Southwest China zone, VI = Central China zone, VII = South China zone (Additional file 3: Materials and Methods). Source data are provided in the Additional file 4

We mapped all the recorded mite species on the seven biogeographic regions in China, four of which (Central China, North China, South China and Southwest China), hosting 251, 141, 88 and 60 mite species, respectively (Additional file 4). At the prefecture level, Liangshan Yi Autonomous Prefecture located in the Southwest China and Yan’an City located in the North China had the highest mite-richness, each reporting over 180 mite species (Fig. 1).

### Risk mapping and risk factors for the predominant mite species

Most mite genera showed region-specific distribution (Additional file 3: Fig. S4–10). To determine the ecological suitability of blood-sucking mites, we performed ecological modeling for each of the 21 most predominant mite species. All models attained decent accuracy with the average testing AUC ranging from 0.76 to 0.95 (Table 1) and the testing partial AUC ratio ranging from 1.21 to 1.80, although the significant ecoclimatic and environmental variables differed between these species (Additional file 3: Table S6–S9).

**Fig. 2:**
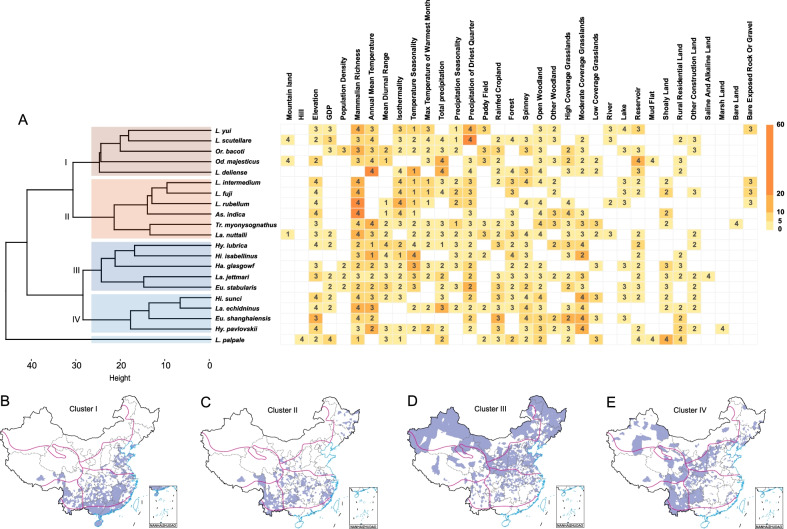
Clustering of mite species based on their ecological features and spatial distributions at the county level. The dendrogram in panel **A** displays the clusters I‒IV of mite species (Additional file 3: Materials and Methods). The features used for clustering are three quantities associated with each predictor in the BRT models. Two of the three quantities were displayed in **A** to indicate the possible level of ecological suitability: relative contributions (colors in ascending order from yellow to red) and standardized median value of the predictor (numbers in the heatmap) among counties with mite occurrence (numbers 1‒4 indicate the position of this median in reference to the quartiles of this predictor among all counties). **B**‒**E** indicate the spatial distribution of the four clusters (clusters I‒IV). The boundaries of the provinces and seven biogeographic zones are shown as black and red lines, respectively. Source data are provided in Additional file 1

Based on these models, mammalian richness and annual mean temperature were the two most common important predictors, contributing ≥ 5% to the ensemble of models for 18 and 15 mite species, respectively, followed by precipitation of driest quarter which contributed ≥ 5% to models for 10 mite species (Fig. 2, Additional file 3: Table S6–9). The same predictor, however, might have driven the risk in different directions for different mite species (Additional file 3: Fig. S11–31). For example, a high mammalian richness was associated with a high probability of presence for majority of the 18 mite species, e.g., *Ascoschoengastia indica* and *L. rubellum*, but with a low probability for *L. palpale* (Additional file 3: Fig. S18–S19, S29).

The model-predicted suitable habitats for the 21 mite species were much more spatially extensive than what was actually observed, 121‒885% greater in the number of affected counties, 92‒570% in the area, and 129‒795% in the population size (Table 1, Additional file 3: Fig. S32–35). *La. echidninus* was predicted to be the most influential species, potentially affected 744 million people in 1,493 counties, followed by *La. jettmari* and *Eu. stabularis* that affected 517 and 452 million people in 1,046 and 861 counties, respectively (Table 1). Areas with highly suitable habitats of these three mite species collectively covered nearly all densely populated areas in China, mainly involving PLADs in the central, eastern, northern and southwestern China (Additional file 3: Fig. S34–35). *Ha. glasgowf*, *La. echidninus*, *La. jettmari*, and *Eu. stabularis* mites were the top four mite species affecting largest areas ranging 2.1‒4.6 million km^2^ (Table 1).

### Ecological clustering of mite species

Based on the ecological similarity represented by the environmental and ecoclimatic predictors, the 21 predominant mite species were grouped into four clusters with a clear pattern of spatial aggregation (Fig. 2). *L. yui*, *L. scutellare*, *Or. bacoti*, *Od. majesticus* and *L. deliense* constituted Cluster I that covered the vast region in northern China that stretched over biogeographic zones II, IV, V, VI and VII. This cluster was characterized by middle to high levels of elevation, mammalian richness and annual mean temperature, as well as relatively weak temperature seasonality and precipitation seasonality (Fig. 2A and B, Additional file 3: Fig. S11–15). *L. intermedium*, *L. fuji*, *L. rubellum*, *As. indica*, *Tricholaelaps myonysognathus* and *La. nuttalli* were grouped into Cluster II, which was mainly found in biogeographic zones I, IV, V, and VI in southeastern and southwestern China, featuring high levels of mammalian richness and isothermality, and low levels of temperature seasonality and max temperature of warmest month (Fig. 2A and C, Additional file 3: Fig. S16–21). *Hy. lubrica*, *Hi*. *isabellinus*, *Ha. glasgowf*, *La. jettmari* and *Eu. stabularis* were grouped into Cluster III that was mainly distributed in biogeographic zones I–VI, characterized by low-medium mammalian richness, low annual mean temperature and isothermally, high temperature seasonality, and medium coverage by forest and spinney (Fig. 2A and D, Additional file 3: Fig. S22–26). Cluster IV, composed of *Hi. sunci*, *La. echidninus, Eu. shanghaiensis* and *Hy. pavlovskii*, were mainly distributed in biogeographic zones I–VI in central and southwestern China, which are covered by spinney, open woodland and grasslands and characterized by high mammalian richness and medium annual mean temperature (Fig. 2A and E, Additional file 3: Fig. S27–30). *L. palpale*, although shared similar geographic distribution to Cluster III, was unique for its ecological niche and thus not clustered with others (Fig. 2A, Additional file 3: Fig. S31).

### Distribution of mite-associated agents

**Fig. 3:**
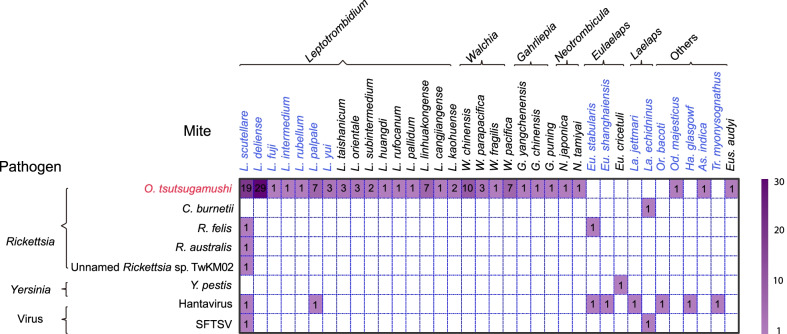
The matrix of mite species and the mite-associated agents in China from 1978 to 2020. The mite species marked in blue and mite-associated agents marked in red were taken into consideration in the BRT models. The colors and numbers of cells indicate the number of relative literatures. Source data are provided in Additional file 2

**Fig. 4:**
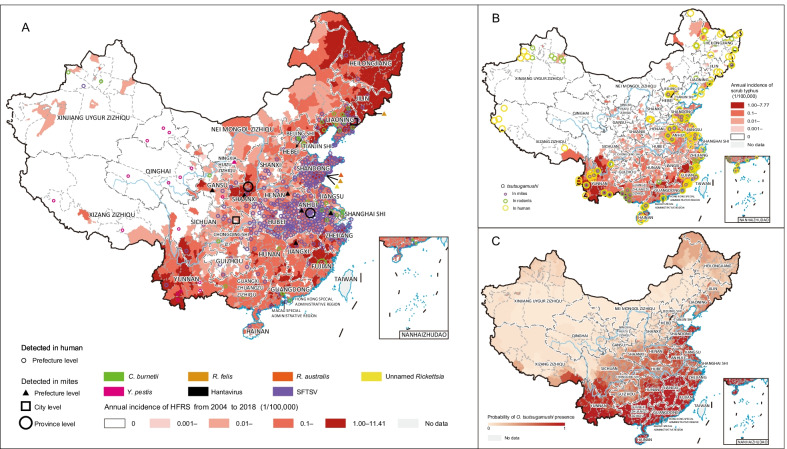
The distribution of mite-associated agents detected in mites, human and the reported and model-predicted distribution of *O. tsutsugamushi* in China. **A** Locations of hantavirus, SFTSV, *Y. pestis*, *R. felis*, *R. australis*, *C. burnetii* and unnamed *Rickettsia* sp. detected in mites at prefecture, city and province levels, locations of SFTSV (2010 ‒2018), *Y. pestis* (2004 ‒2018) and *C. burnetii* (2004 ‒2018) detected in human, and reported annual incidence rate of human hemorrhagic fever with renal syndrome (2004 ‒2018) (Additional file 3: Materials and Methods); **B** Reported annual incidence rate of human scrub typhus from 2010 to 2018 and locations of *O. tsutsugamushi* detected from mites, rodents and human; **C** Spatial distribution of model-predicted probabilities of *O. tsutsugamushi* presence. Source data are provided in Additional file 2

A total of eight mite-associated agents were detected from 36 mite species in 170 counties in China, including five *Rickettsia* species, one *Yersinia* and two viruses (Figs. 3, 4A, Additional file 3: Table S2). *L. scutellare* is the mite species harboring the highest variety of mite-borne agents, including four *Rickettsia* species (*O. tsutsugamushi*, *R*. *felis*, *R*. *australis* and an unnamed *Rickettsia* sp. TwKM02) and two viruses [hantavirus and severe fever with thrombocytopenia syndrome virus (SFTSV)]. Other competent mite species that carry two or more agents are *Eu. stabularis* (2 agents), *L. palpale* (2), and *La. echidninus* (2). Each of the other 32 mite species was found to carry only one agent (Fig. 3).

*O. tsutsugamushi*, the causative pathogen of scrub typhus, was detected from 28 mite species in 33 counties, mostly distributed in northeastern and southeastern China (Fig. 4B). The most frequently reported carriers of *O. tsutsugamushi* were *L. delicense*, located in 22 counties of Fujian, Guangxi, and Yunnan provinces, and *L. scutellare*, located in 13 counties of Hebei, Henan, and Shandong provinces. Sporadic detections of *O. tsutsugamushi* were reported for the remaining 26 mite species, each of which was recorded in < 10 counties mainly in southeastern China.

In *Rickettsia,* besides *O. tsutsugamushi, Coxiella burnetii* also caused human disease, as the causative pathogen of Q fever and a tick-borne pathogen, which was also isolated from *La. echidninus* in Fujian Province (Fig. 3, Fig. 4A). One of the other three *Rickettsia* detected from mites, *R. felis*, originally detected in fleas, ticks and their parasitizing animal hosts, was additionally detected in *Eu. stabularis* by polymerase chain reaction (PCR) in the Changbai County, Liaoning Province for the first time in the year of 2012. In addition, in Huangdao District (district is an urban administrative area equivalent to county) of Qingdao City, *R. felis*, *R. australis* and an unnamed *Rickettsia* sp. TwKM02 were detected in *L. scutellare* by one-step real-time polymerase chain reaction (RT-PCR) and nested PCR. The only *Yersinia* of mite-associated agents, *Y. pestis*, the causative pathogen of plague and commonly known as rodent-borne pathogen, was additionally detected in *Eu*. *cricetuli* in Ningxia Hui Autonomous Region in the year of 1989 (Fig. 4A)*.* During 2004 ‒2018, totally 1,253 and 44 human cases had confirmed infections with *C. burnetii* and *Y. pestis*, respectively, in the mainland of China.

The two viruses that were detected in mites were hantavirus, the causative pathogen of HFRS, and SFTSV (Additional file 3: Table S2) [41]. A total of eight mite species, including *L. scutellare*, *L. palpale*, *Eu. stabularis*, *Eu. shanghaiensis*, *La. jettmari*, *Or. bacoti*, *Ha. glasgowf* and *Tr. myonysognathus*, were reported to harbor hantavirus as determined by PCR, RT-PCR, or cultural isolation, but the detection was mainly limited to central and eastern China (Anhui, Henan, Jiangsu, Jiangxi, Shaanxi, Sichuan, and Liaoning provinces) (Fig. 3, Fig. 4A). During 2004 ‒2018, in total 189,237 HFRS human cases with 1,772 deaths [case fatality rate (CFR): 0.94%] had been reported in the mainland of China, the highest disease burden of HFRS in the world. SFTSV, an emerging tick-borne pathogen that was firstly identified in China in 2010 [42], were detected in *L. scutellare* and *La. echidninus* in Donghai County, Jiangsu Province for the first time in the year of 2012. By the end of 2018, a total of 7,721 SFTS human cases with 810 deaths (CFR: 10.5%) had been confirmed in the mainland of China, and the interregional spread of the virus continued in recent years (Fig. 4A).

### Ecological analysis and risk mapping for *O. tsutsugamushi*

Among all eight investigated mite-associated agents recorded, *O. tsutsugamushi* was selected for further study of its ecological niches and risk mapping using BRT models. Other agents were not modeled due to either an insufficient number of detections in mites or the incompetence of mites as transmission vectors, e.g., hantavirus and SFTSV. Based on the mapping of observed detections, a high spatial consistency was observed between the detection of *O. tsutsugamushi* in mites and the high incidence areas of human scrub typhus (Fig. 4B). At the county level, the distribution of model-predicted probabilities of occurrence of *O. tsutsugamushi* (Fig. 4C) largely resembled that of reported incidences of human scrub typhus (Fig. 4B). A total of 1,224 counties were predicted with probability of *O. tsutsugamushi* presence exceeding 50%, where approximately 71.5 million people reside. Five predictors significantly contributed to the presence of *O. tsutsugamushi*. Total precipitation was by far the most influential predictor with a relative contribution (RC) of 38.6% (Table 2), and it was also a leading predictor for the presence of major mite carriers of *O. tsutsugamushi* (Additional file 3: Table S6, S7). Other important contributors include mean diurnal range of air temperature (RC = 17.6%), distribution of *L. deliense* (RC = 10.3%), distribution of *Od. majesticus* (RC = 6.0%) and precipitation of driest quarter (RC = 5.3%).

**Table 2:** BRT-model-estimated mean (standard deviation) relative contributions of major ecoclimatic and environmental factors to the spatial distributions of *O. tsutsugamushi*

Category	Variable	*O. tsutsugamushi*
Ecoclimatic	Total precipitation	38.60 (5.21)
	Mean diurnal range	17.59 (5.23)
	Precipitation of driest quarter	5.33 (1.76)
	Temperature seasonality	4.67 (0.72)
	Annual mean temperature	2.74 (0.70)
Environmental	High coverage grasslands	3.52 (0.45)
	Mammalian richness	3.08 (0.60)
Mite Distribution	*L. deliense*	10.25 (0.82)
	*Od. majesticus*	5.97 (0.89)
	*L. scutellare*	4.68 (0.80)
	*L. palpale*	3.58 (0.80)
AUC	Train	0.943 (0.940‒0.946)
	Test	0.923 (0.905‒0.935)
Partial AUC ratio	Train	1.33
	Test	1.30

Mean AUCs (95% percentiles) and partial area AUC ratio (calculated at tolerance level of 0.2) are given

*AUC* areas-under-curve

*O. tsutsugamushi* ecologically prefers habitats with a total annual precipitation exceeding 800 mm, which covers all the provinces to the south of Qinling Mountains (latitude 32–34° N) in China. In addition, high-risk areas of *O. tsutsugamushi* presence also feature a low mean diurnal range and a high precipitation of driest quarter, as well as higher probabilities for the presence of *L. deliense* and *Od. majesticus* (Additional file 3: Fig. S36).

## Discussion

We assembled the most comprehensive records of blood-sucking mites and mite-associated agents in China covering more than 40 years of research. The cross-tabulation of 100 genera and 549 species of mites in association with the agents they harbor was established. Compared to a previous study that reviewed less than 300 mite species in single province [43], our study is not only more widely scoped but also more in-depth, as we used a robust machine-learning algorithm to predict the potential habituating regions of predominant mites (particularly *L. palpale*, *L. intermedium*, *La. echidninus*, *Eu. shanghaiensis*), which could be up to as tenfold large as what have been observed (Table 1). The ability of mites to feed on a wide range of domestic and wildlife hosts, to reproduce asexually, and to survive in various environmental conditions has likely contributed to their establishment throughout the country. The much wider model-predicted than observed spatial distributions could have resulted from limited field investigations or incomplete sampling; yet it is also possible that our models were missing unmeasured risk drivers and thus overestimated the scopes.

The BRT model, a machine learning algorithm, has been widely used for risk mapping of infectious diseases such as leishmaniasis [44], avian influenza [45] and even COVID-19 [46], due to its advantages in allowing for nonlinear relationships between outcomes and covariates and multicollinearity among covariates [47]. Based on BRT models, estimated ecological niches for mites are complex, and the key predictors differ even within the same genus. It is therefore critical to group mite species by their ecological characteristics, in addition to their genera, to better understand the overall risk of mite exposure at any given place. We found four clusters of mite species that share comparable ecological niches and geographic distributions. Such clustering offers additional information for risk assessment and field investigation of mite-borne agents. Among the ecoclimatic and socioenvironmental factors, the most influential for mite ecology are mammalian richness and annual mean temperature (Additional file 3: Table S6–9). The significant role of mammalian richness is expected, as availability of host species is the most important determinant in ectoparasite community assembly. The composition, richness and abundance of mites are highly influenced by host species [48]. Meanwhile, mites have tiny body size and thin body wall, which determines their low ability to regulate internal temperature and high vulnerability to fluctuation of the ambient temperature. Moreover, temperature dramatically influences the developmental process of mites, e.g., the hatching duration is prolonged under low temperature. According to our modeling results, ambient temperature of 10 ‒20 ℃ might be optimal for mite propagation (Fig. S11–31). Our analyses also disclosed the nontrivial role of vegetation coverage. Forest, spinney, grassland and woodland constitute the suitable habitat types for mites and their hosts. Human settlements influence the distributions of some mite species as well. For example, the distributions of both *Eu. shanghaiensis* and *Hy. pavlovskii* were positively associated with the coverage of rural residential land and rainfed cropland, likely due to their tendency of parasitizing farming livestock. The potential preference of *L. deliense*, *Od. majesticus*, *Hy. lubrica* and *L. yui* for a high coverage of reservoir, was also associated with the need of these species for humid microclimates [49].

*La. echidninus* is by far the most widely distributed mite species in the mainland of China, affecting over 54% of the nation’s population in 1,493 counties of the central and southwestern provinces. *La. echidninus* harbors SFTSV, the etiological pathogen for a disease endemic in Central and Eastern China with a relatively high CFR. However, the competence of mites as a vector for SFTSV is likely low as detection in mites has been rare and there has been no evidence for transmission of SFTSV from mites to humans. Our study prompted the need for close surveillance and laboratory test of SFTSV in mites to monitor the evolvement of mites as a carrier of SFTSV. These findings may also be implicative for southeastern Asian countries where *La. echidninus* was frequently reported, including Malaysia, Thailand and Bangladesh [50–52]. In addition, studies have reported hantavirus detected in mites and laboratory evidence of bite transmission of hantavirus from mites to mice, indicating that mites might have been playing an untrivial role in the circulation of hantavirus between vectors and animal hosts [7, 18, 53].

During 2006 ‒2016, a total of 4,382 human cases infected *O. tsutsugamushi*, including 57 deaths (CFR: 1.30%), were reported, which were mostly distributed in coastal provinces in eastern and southeastern China and Yunnan Province of the southwestern China [54]. In China, there has been increasing interest in the spatiotemporal patterns of human cases with scrub typhus [37, 54, 55]. Compared with previous studies, we investigated the ecological drivers and suitability for *O. tsutsugamushi* in a more general setting, i.e., our data incorporate not only human cases but also detections in vectors and animal hosts. In addition, our analysis was conducted at a more refined spatial resolution. Our findings bear some similarity with those from a previous ecological analysis on human cases of scrub typhus [37]. For example, the previous study found precipitation, sunshine hour, temperature, relative humidity and percentage coverage of crop field to be key predictors for the scrub typhus incidence. However, the current work identified more precise climatic conditions, e.g., total precipitation, mean diurnal range and precipitation of driest quarter (Table 2). Moreover, this study uniquely identified the presence of two mites, *L. deliense* and *Od. majesticus*, as significant contributors to the ecology of *O. tsutsugamushi*.

The current study unfolded a wide geographic range of 1,224 counties (2,190,000 km^2^) with a large population (71.5 million people) at risk of potential *O. tsutsugamushi* occurrence, much more extensive than the observed range. Surveillance of scrub typhus and field monitoring of *O. tsutsugamushi* should be strengthened in high-risk areas in southern China, especially surrounding middle and downstream areas of the Yangtze River. On the other hand, ecological models are known to predict risk areas much larger than actual habitats, and therefore surveillance efforts need to be balanced between risk and cost, e.g., using sampling scheme informed by the risk map we produced.

These mite-associated agents have also been detected in ticks, rodents or human blood samples [56, 57]. Among mite-borne agents detected in China, SFTSV and an unnamed *Rickettsia* sp. TwKM02 were newly identified in the past two decades. Moreover, due to the geographic expansion of blood-sucking mites, increasing international travel and more advanced detection technology, more agents would be discovered in blood-sucking mites in the future [58]. These mite-associated agents may not be transmitted to human through mites, but it is necessary to keep alert to mites for the probable vector competence.

Our study was subject to several limitations. Firstly, the survey locations of mites were extracted from literature and monographs, thus unlikely to be sampled randomly. However, the surveyed 759 counties cover 27% counties extending over all biogeographical zones in the nation (Additional file 3: Fig. S1), supporting the representativeness of the sampled sites. In addition, we used a statistical approach, weighting the counties by estimated probabilities of being surveyed, to reduce the potential sampling bias in the BRT models. Secondly, the high AUC values of the fitted BRT models do not necessarily reflect perfect goodness of fit and should be interpreted with caution, as the absence data are associated with high uncertainty [59]. Such uncertainty is inherent in cross-sectional surveys. Thirdly, “county” is an administrative unit rather than a natural geographical unit, and some large counties may have variable ecological and environmental conditions. Our ecological modeling was based on the mean levels of predictors in each county, ignoring the potential variability and possibly leading to ecological fallacy. Nevertheless, most counties have sizes (inter-quartile range: 703–2,789 km^2^) appropriate for ecological studies of mites and associated agents, and county is the finest resolution to locate most reported detections of mites and agents. Finally, we only focused on blood-sucking mites for the current investigation, due to their implication for public health.

## Conclusions

Our study provides a comprehensive update on the distribution of mites and mite-related pathogens that are relevant to public health in China. In particular, the risk map of *O. tsutsugamushi* can be useful for predicting the expansion of scrub typhus in the country, and the related methodology can be generalized to other regions of the world that might be ecologically suitable for blood-sucking mites. Our study also highlights the importance of integrating field survey, landscape, and meteorological data for risk assessment of mite-associated infection, especially when field surveillance data are limited. Given the popularity of outdoor recreational activities, more efforts should be dedicated to promote the public awareness of health risk posed by mites in high-risk areas.

## Supplementary Information

**Additional file 1.** The dataset for mites.

**Additional file 2.** The dataset for mite-borne agents.

**Additional file 3.** Additional information includes additional materials and methods, additional results, 36 figures, 9 tables and additional references.

**Additional file 4.** The number of mite species recorded in each county and seven biogeographic regions in China.

**Additional file 5. **The code of BRT model.

## Data Availability

The datasets supporting the conclusions of this article are included within the article and its additional files (Additional files 1, 2 and 4).

## Abbreviations

VBI: Vector-borne infections
BRT: Boosted regression tree
ROC: Receiver-operating characteristic
AUC: Areas under the curve
SFTSV: Severe fever with thrombocytopenia syndrome virus
PCR: Polymerase chain reaction
RT-PCR: Real-time polymerase chain reaction
CFR: Case fatality rate
RC: Relative contribution
