# Supplementary material for: Mapping the distributions of blood-sucking mites and mite-borne agents in China: a modeling study
Source: Infect Dis Poverty. 2022 Apr 9;11:41. doi: 10.1186/s40249-022-00966-0 (PMC8994071; doi:10.1186/s40249-022-00966-0)
Supplement: Supplementary file 3 — Additional file 3. Additional information includes additional materials and methods, additional results, 36 figures, 9 tables and additional references. [file 40249_2022_966_MOESM3_ESM.docx]

**Supplementary information**

Supplement to: Tao Wang, et al. Mapping the distributions of blood-sucking mites and mite-associated agents in China: a modeling study

**Table of Contents**

| **Page** | **Item** |
| --- | --- |
| 4 | Supplementary Materials and Methods |
| 5 | Supplementary Results |
| 6 | Fig. S1: The flow diagram of literature review. |
| 7 | Fig. S2: The weights of counties with investigation of mites by logistic model. |
| 8 | Fig. S3: The spatial distribution of the 759 counties with at least one record of blood-sucking mites (yellow) from 1978 to 2020 in China. |
| 9 | Fig. S4: The spatial distribution of the blood-sucking mite genus *Laelaps* recorded at county level from 1978 to 2020 in China. |
| 10 | Fig. S5: The spatial distribution of the blood-sucking mite genus *Haemolaelaps* recorded at county level from 1978 to 2020 in China. |
| 11 | Fig. S6: The spatial distribution of the blood-sucking mite genus *Eulaelaps* recorded at county level from 1978 to 2020 in China. |
| 12 | Fig. S7: The spatial distribution of the blood-sucking genus *Hirstionyssus* recorded at county level from 1978 to 2020 in China. |
| 13 | Fig. S8: The spatial distribution of the blood-sucking mite genus *Leptotrombidium* recorded at county level from 1978 to 2020 in China. |
| 14 | Fig. S9: The spatial distribution of the blood-sucking mite genus *Hamogamasus* recorded at county level from 1978 to 2020 in China. |
| 15 | Fig. S10: The spatial distribution of the blood-sucking mite genus *Hypoaspis* recorded at county level from 1978 to 2020 in China. |
| 16 | Fig. S11: The mean curves (red) and 95% percentiles (gray) for the effects of major predictors (RC≥5%) on the logit-transformed probability of occurrence of *L. yui* based on the ensemble of BRT models. |
| 17 | Fig. S12: The mean curves (red) and 95% percentiles (gray) for the effects of major predictors (RC≥5%) on the logit-transformed probability of occurrence of *L. scutellare* based on the ensemble of BRT models. |
| 18 | Fig. S13: The mean curves (red) and 95% percentiles (gray) for the effects of major predictors (RC≥5%) on the logit-transformed probability of occurrence of *Or. bacoti* based on the ensemble of BRT models. |
| 19 | Fig. S14: The mean curves (red) and 95% percentiles (gray) for the effects of major predictors (RC≥5%) on the logit-transformed probability of occurrence of *Od. majesticus* based on the ensemble of BRT models. |
| 20 | Fig. S15: The mean curves (red) and 95% percentiles (gray) for the effects of major predictors (RC≥5%) on the logit-transformed probability of occurrence of *L. deliense* based on the ensemble of BRT models. |
| 21 | Fig. S16: The mean curves (red) and 95% percentiles (gray) for the effects of major predictors (RC≥5%) on the logit-transformed probability of occurrence of *L. intermedium* based on the ensemble of BRT models. |
| 22 | Fig. S17: The mean curves (red) and 95% percentiles (gray) for the effects of major predictors (RC≥5%) on the logit-transformed probability of occurrence of *L. fuji* based on the ensemble of BRT models. |
| 23 | Fig. S18: The mean curves (red) and 95% percentiles (gray) for the effects of major predictors (RC≥5%) on the logit-transformed probability of occurrence of *L. rubellum* based on the ensemble of BRT models. |
| 24 | Fig. S19: The mean curves (red) and 95% percentiles (gray) for the effects of major predictors (RC≥5%) on the logit-transformed probability of occurrence of *As. indica* based on the ensemble of BRT models. |
| 25 | Fig. S20: The mean curves (red) and 95% percentiles (gray) for the effects of major predictors (RC≥5%) on the logit-transformed probability of occurrence of *Tr. myonysognathus* based on the ensemble of BRT models. |
| 26 | Fig. S21: The mean curves (red) and 95% percentiles (gray) for the effects of major predictors (RC≥5%) on the logit-transformed probability of occurrence of *La. nuttalli* based on the ensemble of BRT models. |
| 27 | Fig. S22: The mean curves (red) and 95% percentiles (gray) for the effects of major predictors (RC≥5%) on the logit-transformed probability of occurrence of *Hy. lubrica* based on the ensemble of BRT models. |
| 28 | Fig. S23: The mean curves (red) and 95% percentiles (gray) for the effects of major predictors (RC≥5%) on the logit-transformed probability of occurrence of *Hi. isabellinus* based on the ensemble of BRT models. |
| 29 | Fig. S24: The mean curves (red) and 95% percentiles (gray) for the effects of major predictors (RC≥5%) on the logit-transformed probability of occurrence of *Ha. glasgowf* based on the ensemble of BRT models. |
| 30 | Fig. S25: The mean curves (red) and 95% percentiles (gray) for the effects of major predictors (RC≥5%) on the logit-transformed probability of occurrence of *La. jettmari* based on the ensemble of BRT models. |
| 31 | Fig. S26: The mean curves (red) and 95% percentiles (gray) for the effects of major predictors (RC≥5%) on the logit-transformed probability of occurrence of *Eu. stabularis* based on the ensemble of BRT models. |
| 32 | Fig. S27: The mean curves (red) and 95% percentiles (gray) for the effects of major predictors (RC≥5%) on the logit-transformed probability of occurrence of *Hi. sunci* based on the ensemble of BRT models. |
| 33 | Fig. S28: The mean curves (red) and 95% percentiles (gray) for the effects of major predictors (RC≥5%) on the logit-transformed probability of occurrence of *La. echidninus* based on the ensemble of BRT models. |
| 34 | Fig. S29: The mean curves (red) and 95% percentiles (gray) for the effects of major predictors (RC≥5%) on the logit-transformed probability of occurrence of *Eu. shanghaiensis* based on the ensemble of BRT models. |
| 35 | Fig. S30: The mean curves (red) and 95% percentiles (gray) for the effects of major predictors (RC≥5%) on the logit-transformed probability of occurrence of *Hy. pavlovskii* based on the ensemble of BRT models. |
| 36 | Fig. S31: The mean curves (red) and 95% percentiles (gray) for the effects of major predictors (RC≥5%) on the logit-transformed probability of occurrence of *L. palpale* based on the ensemble of BRT models. |
| 37 | Fig. S32: The predicted county-level distributions of the Cluster Ⅰ, averaged over the ensemble of BRT models (a) *L. yui*, (b) *L. scutellare*, (c) *Or. bacoti*, (d) *Od. majesticus* and (e) *L. deliense*. |
| 38 | Fig. S33: The predicted county-level distributions of the Cluster Ⅱ, averaged over the ensemble of BRT models (a) *L. intermedium*, (b) *L. fuji*, (c) *L. rubellum*, (d) *As. indica*, (e) *Tr. myonysognathus* and (f) *La. nuttalli*. |
| 39 | Fig. S34: The predicted county-level distributions of Cluster Ⅲ, averaged over the ensemble of BRT models (a) *Hy. lubrica*, (b) *Hi. isabellinus*, (c) *Ha. glasgowf*, (d) *La. jettmari* and (e) *Eu. stabularis*. |
| 40 | Fig. S35: The predicted county-level distributions of the Cluster Ⅳ, averaged over the ensemble of BRT models: (a) *Hi. sunci*, (b) *La. echidninus*, (c) *Eu. shanghaiensis* and (d) *Hy. pavlovskii.* |
| 41 | Fig. S36: The mean curves (red) and 95% percentiles (gray) for the effects of major predictors (RC≥5%) on the logit-transformed probability of occurrence of *O. tsutsugamushi* based on the ensemble of BRT models. |
| 42 | Table S1: The specific number of recorded counties with occurrence and references for each of 551 mite species from 100 genera in the mainland of China from 1978 to 2020. |
| 57 | Table S2: The specific references for pathogens detected in blood-sucking mites in China from 1978 to 2020. |
| 58 | Table S3: The inclusion and exclusion criteria for screening articles. |
| 59 | Table S4: Clustering analysis of eco-climatic predictors at the county level based on pairwise Pearson correlation coefficients. |
| 60 | Table S5: The social, environmental and ecoclimatic variables used for ecological modeling for mite species and mite-borne pathogens at county level in this study. |
| 62 | Table S6: BRT-model-estimated mean (standard deviation) relative contributions of top environmental and ecoclimatic factors (RC≥5%) to the spatial distribution of the Cluster Ⅰ. |
| 63 | Table S7: BRT-model-estimated mean (standard deviation) relative contributions of top environmental and ecoclimatic factors (RC≥5%) to the spatial distribution of the Cluster Ⅱ. |
| 64 | Table S8: BRT-model-estimated mean (standard deviation) relative contributions of top environmental and ecoclimatic factors (RC≥5%) to the spatial distribution of the Cluster Ⅲ. |
| 65 | Table S9: BRT-model-estimated mean (standard deviation) relative contributions of major environmental and ecoclimatic factors (RC≥5%) to the spatial distribution of the Cluster Ⅳ. |
| 66 | Supplementary References |

**Materials and Methods**

**1. Data on** **ecoclimatic and socioenvironmental factors.** A variety of environmental and climatic variables that are commonly used in ecological studies were collected to evaluate the spatial distribution of blood-sucking mite species and mite-associated agents [1-5]. The empirical ecological evidence in the literature and their spatial variability provided basis for the choice of variables. In addition, we focus on ecological variables that are potentially shared by multiple species so that the results can be compared across species.

The climatic data were collected from 2 113 weather surveillance stations in mainland China, covering 71.3% of 1 134 surveyed counties (http://www.nmic.cn/) recorded from 1981 to 2018. The climatic data include average monthly meteorological variables such as temperature, maximum temperature, minimum temperature, relative humidity, and rainfall during the 38 years. For the 750 counties without meteorological stations, the mean values of the nearest five surveillance stations were used as a proxy for their meteorological variables. 19 cross-sectional ecoclimatic variables (BIO01‒19, also called bioclimatic variables recommended by the U.S. Geological Survey) were created and their yearly averages were used as predictors in our risk models [6]. These ecoclimatic variables capture the seasonal trends of different species related to their physiological constraints [7].

Raster-type land cover data of China in year 2005 and 2015 with a resolution of one square kilometer were obtained from the National Earth System Science Data Sharing Infrastructure (http://www.geodata.cn/). While many blood-sucking mite surveys were conducted after 2010, the establishment of the mites’ habitats likely has evolved much longer. Therefore, we used the 2005 land cover data to model the distributions of blood-sucking mite species and mite-associated agents. Elevation data were obtained from the Shuttle Radar Topography Mission (SRTM) archives (http://www.srtm.csi.cigar.org/).

In total, 40 socioenvironmental and ecoclimatic variables at county level were extracted from these data using the ArcGIS Desktop 10.7.0.10450 software (ESRI Inc., Redlands, CA, USA) (Table S5). Data cleaning and reorganization with regard to these variables were performed in the statistical software RStudio Version 1.2.5001.

**2. Spatial mapping.** Recorded occurrences of blood-sucking mites, mite-borne agents and human cases were geo-referenced at the county level when data permit or at the prefecture or province level otherwise. All maps were produced using the ArcGIS Desktop 10.7.0.10450 software and the digital map of China’s administrative divisions was downloaded from Resource and Environment Science and Data Center, Chinese Academy of Sciences (http://www.resdc.cn/).

**3. Clustering mites with similar ecological niches and their spatial distribution.** A hierarchical cluster analysis based on the weighted-average linkage method was performed [8, 9] to explore similarity in ecological niches among the 21 blood-sucking mite species. Predictors that are not influential for all 21 mites were excluded first. For each mite species, three quantities associated with each remaining ecological predictor were calculated as features for clustering. One is the average relative contribution of this predictor in the final 100 BRT models. If the predictor was not included in the final models for this mite species, its relative contribution was set to zero. The second quantity is a measure for the difference in this predictor between case counties (positive for the given blood-sucking mite species) and all counties. We first calculated the median value of this predictor among all case counties and quartile intervals of the predictor among all counties in the nation. We then assigned one of the numbers 1‒4 according to which quartile interval the median lies in, e.g., assign 1 (4) if the median lies in the lowest (highest) quartile. The third quantity is the linear correlation between the predictor and model-predicted presence probabilities of the given mite species among all counties (averaged over the 100 models). These three quantities of all ecological predictors jointly serve as features for clustering. A dendrogram was created to demonstrate the clustering pattern of these 21 mite species, together with a thematic matrix illustrating the features (Fig. 2). This matrix has mite species as rows and predictors as columns. The color of each cell in the matrix shows the average relative contribution and the number shows the quartile (1‒4 for 1st‒4th quartiles) location of the median of cases. To map geographic distributions of the identified clusters of mite species at county level, we define the presence of each cluster as the presence of any mite species in that cluster.

**4. Data on clinical cases of hemorrhagic fever with renal syndrome and scrub typhus.** The county-level data of clinically diagnosed or laboratory-confirmed at clinics and hospitals, including hemorrhagic fever with renal syndrome cases during 2004‒2018 and scrub typhus cases during 2010‒2018, were extracted from the Chinese Scientific Data Center for Public Health (<http://www.phsciencedata.cn>/) and scrub typhus cases were used in the ecological models for the disease prediction.

**Results**

**The integrated database for blood-sucking mite species and mite-associated agents.** Through literature review, we found a total of 24 738 references, 1 839 in English and 22 899 in Chinese, which met our search criteria. With consensus of two independent reviewers, 334 publications met our study inclusion criteria and were used for data extraction, of which 156 reported detections of mite-associated agents. After pooling data from all sources, we obtained an integrated database of 7 417 blood-sucking mite records. The 21 predominant blood-sucking mites belong to 9 genera. For 9 mite genera, we assembled 994, 722, 464, 383, 369, 127, 99, 76 and 61 occurrence records for *Laelaps*, *Leptotrombidium*, *Hirstionyssus*, *Eulaelaps*, *Hypoaspi*, *Ornithonyssus*, *Tricholaelaps,* *Odontacarus* and *Ascoschoengastia*, respectively.

**Supplementary Figures**:

**Figure S1**: **The flow diagram of literature review.**


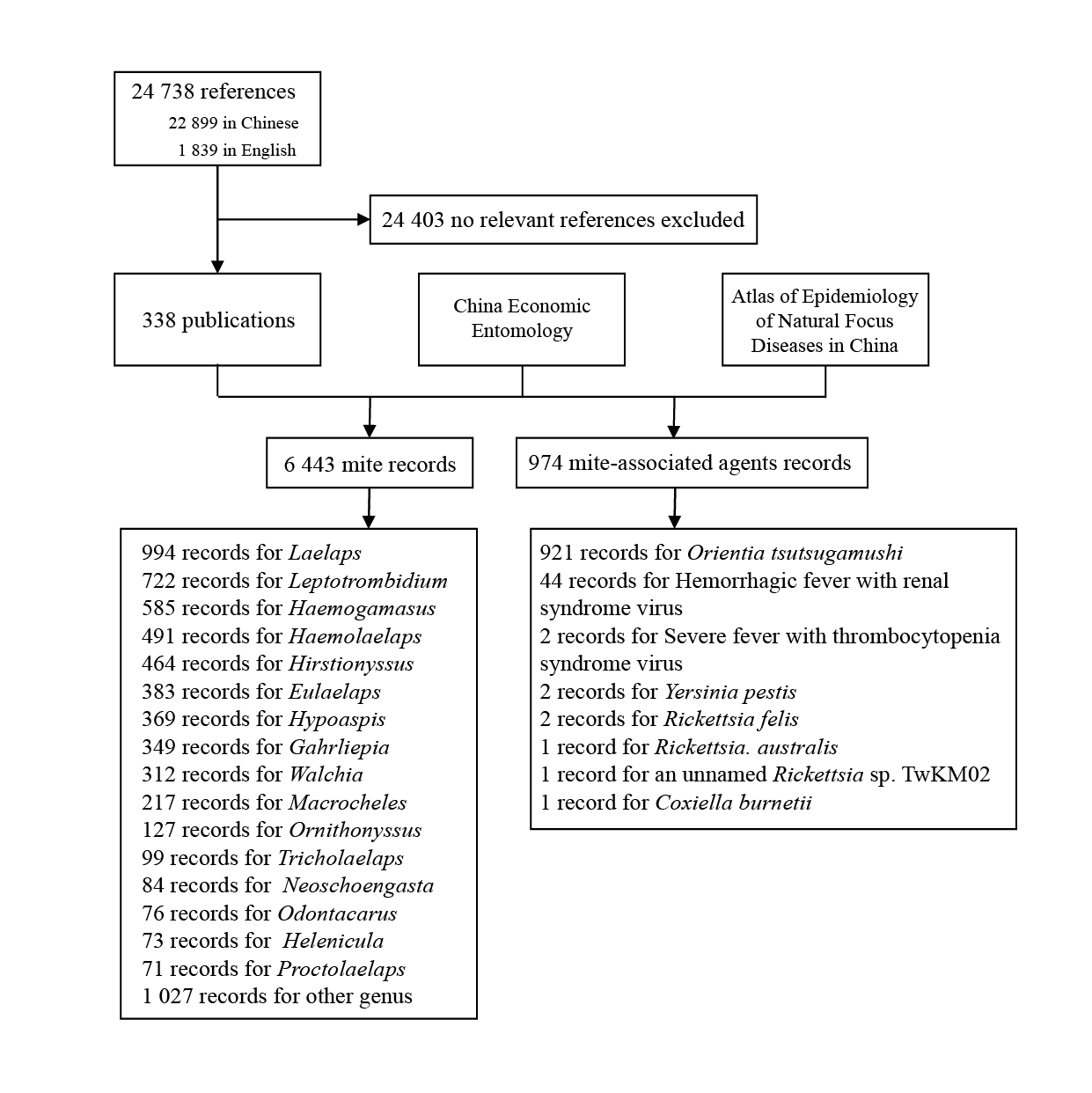


**Figure S2**: **The weights of counties with investigation of mites by logistic model.**


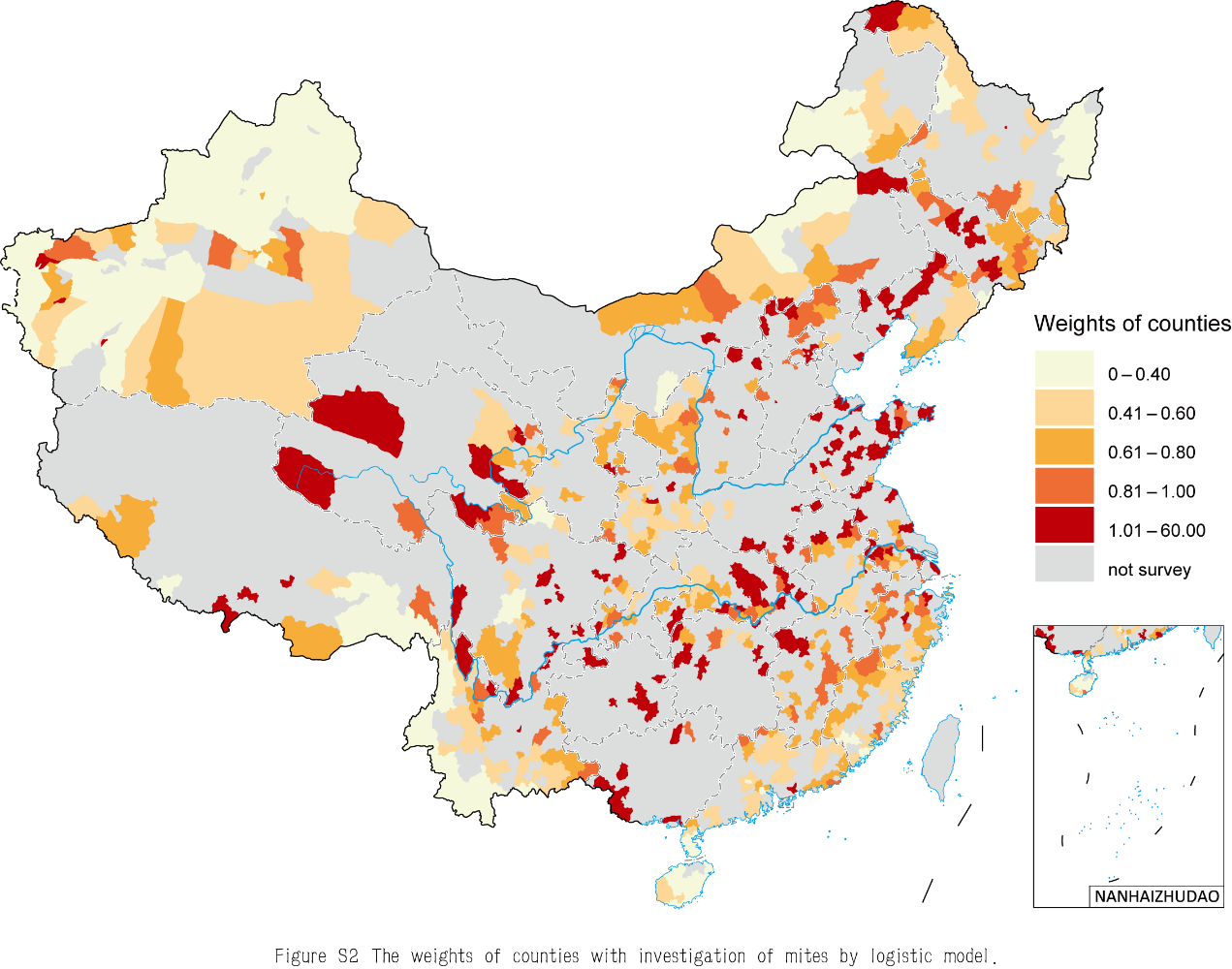


**Figure S3**: **The spatial distribution of the 759 counties with at least one record of blood-sucking mites (yellow) from 1978 to 2020 in China.**


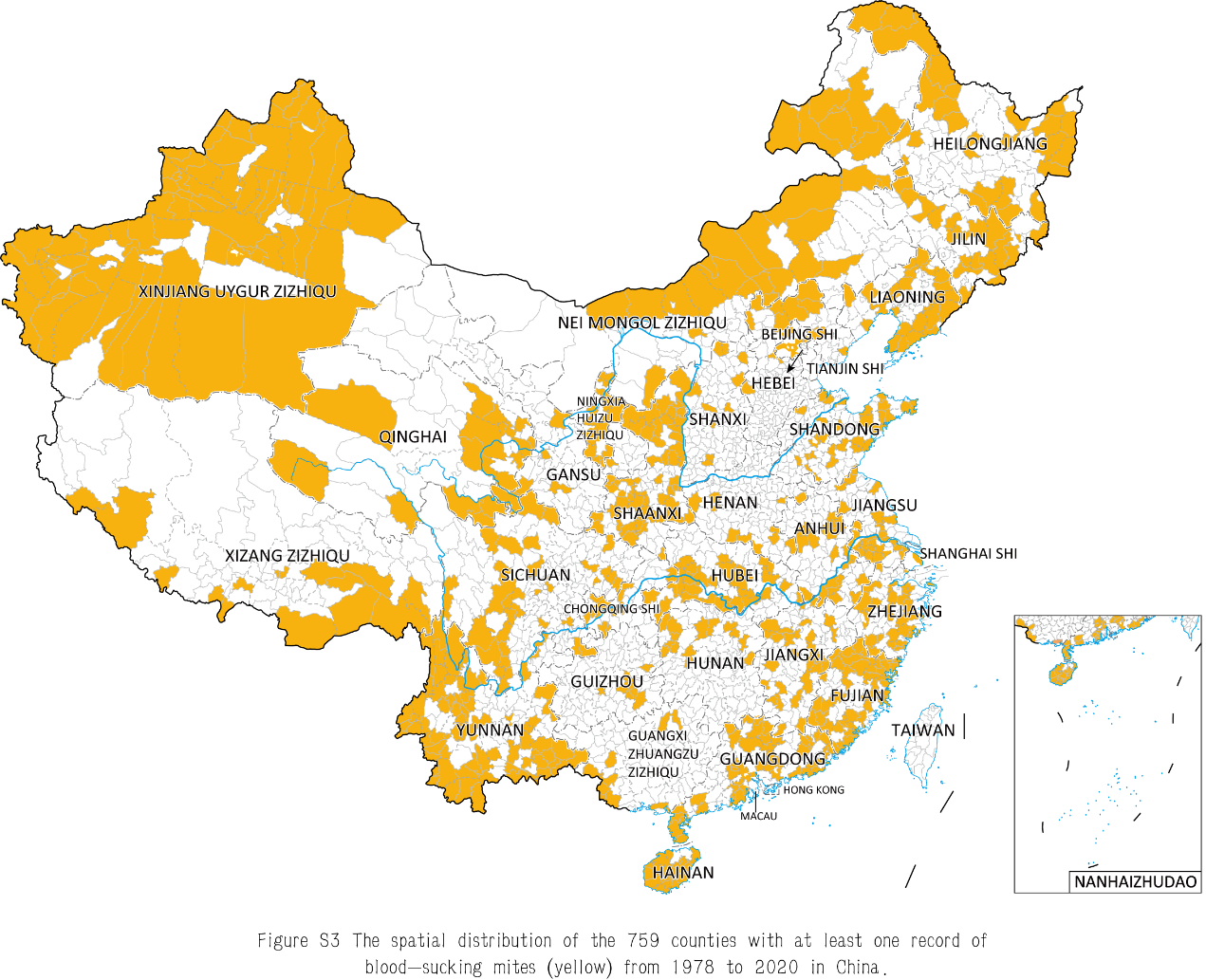


**Figure S4**: **The spatial distribution of the blood-sucking mite genus *Laelaps* recorded at county level from 1978 to 2020 in China.**

**
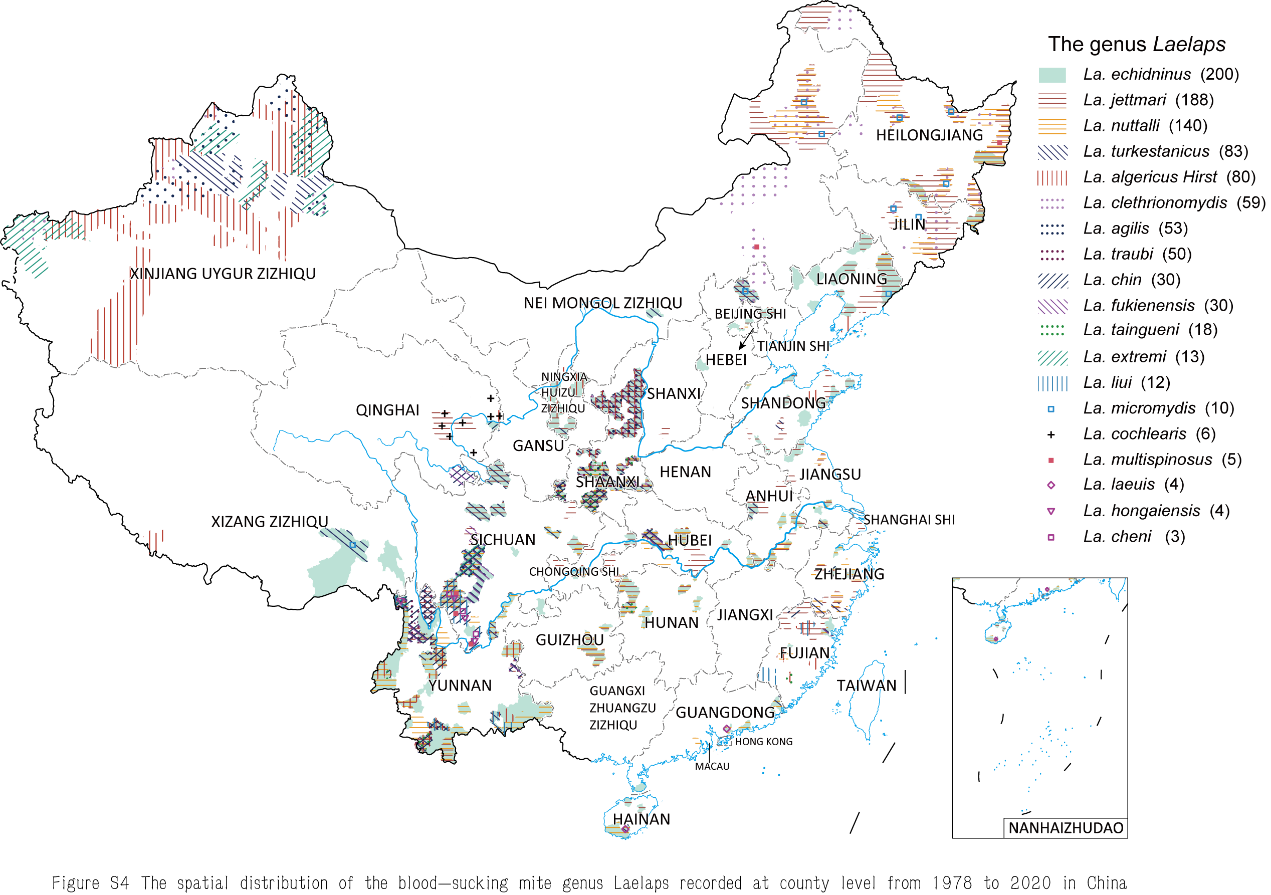
**

**Figure S5**: **The spatial distribution of the blood-sucking mite genus** ***Haemolaelaps* recorded at county level from 1978 to 2020 in China.**

**
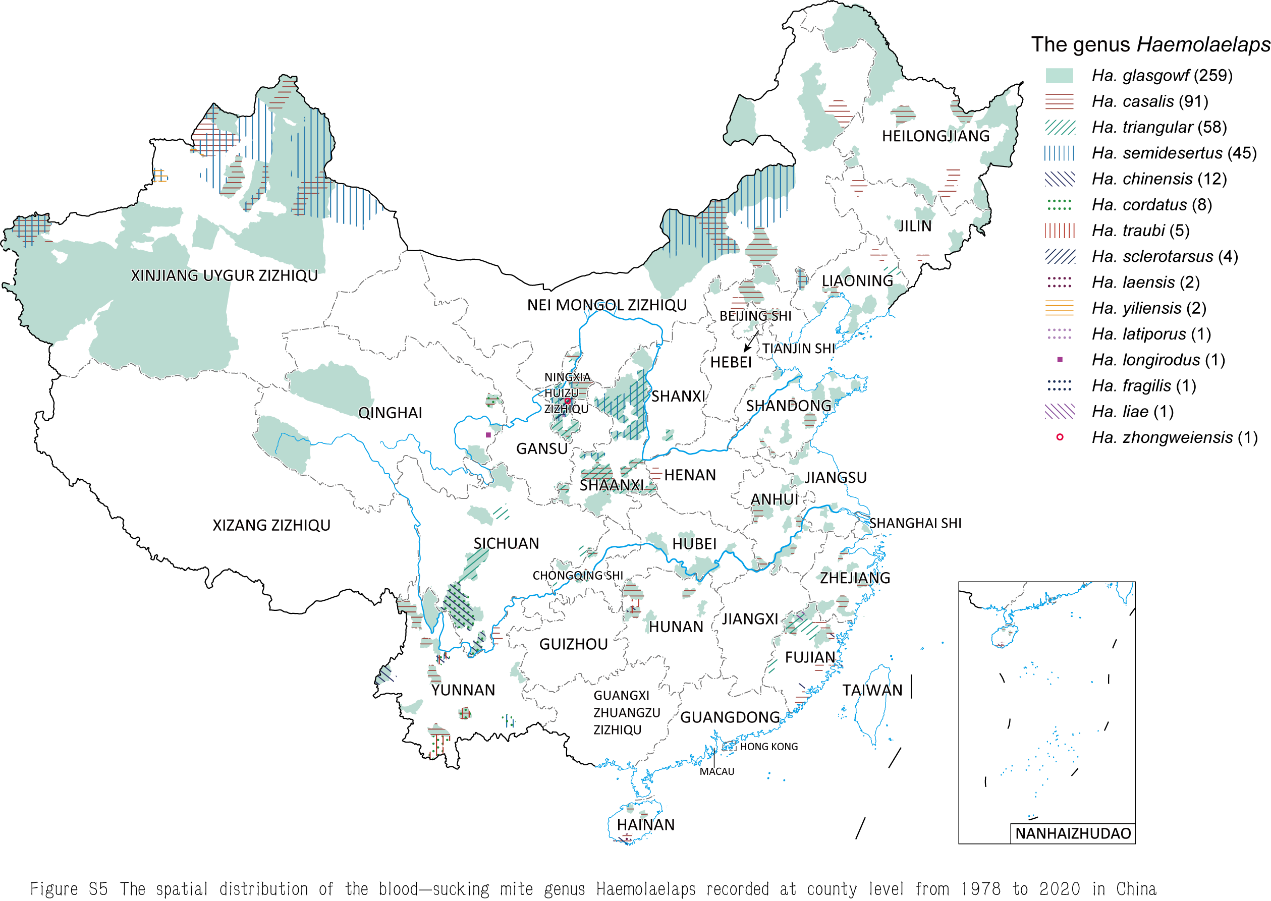
**

**Figure S6**: **The spatial distribution of the blood-sucking mite genus *Eulaelaps* recorded at county level from 1978 to 2020 in China.**

**
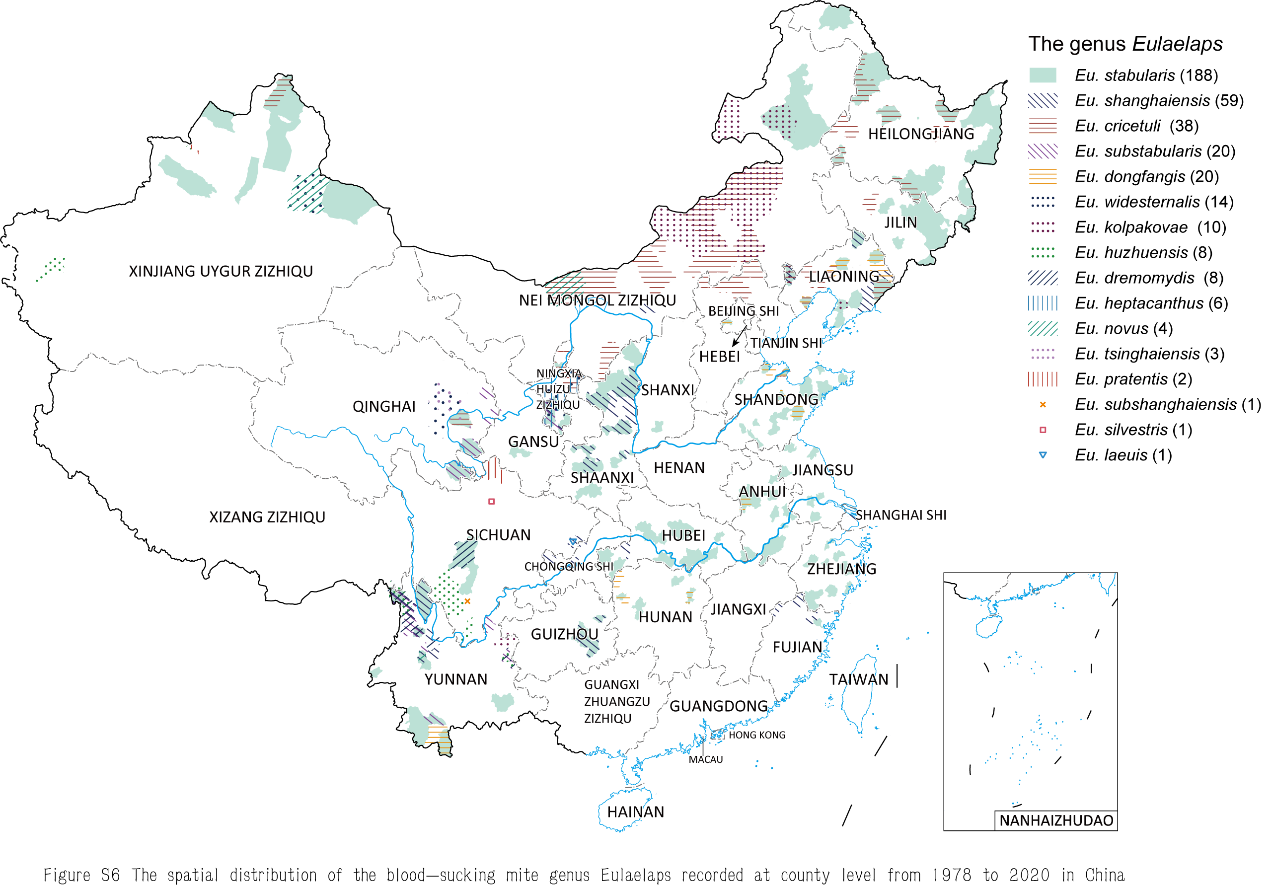
**

**Figure S7**: **The spatial distribution of the blood-sucking genus *Hirstionyssus* recorded at county level from 1978 to 2020 in China.**
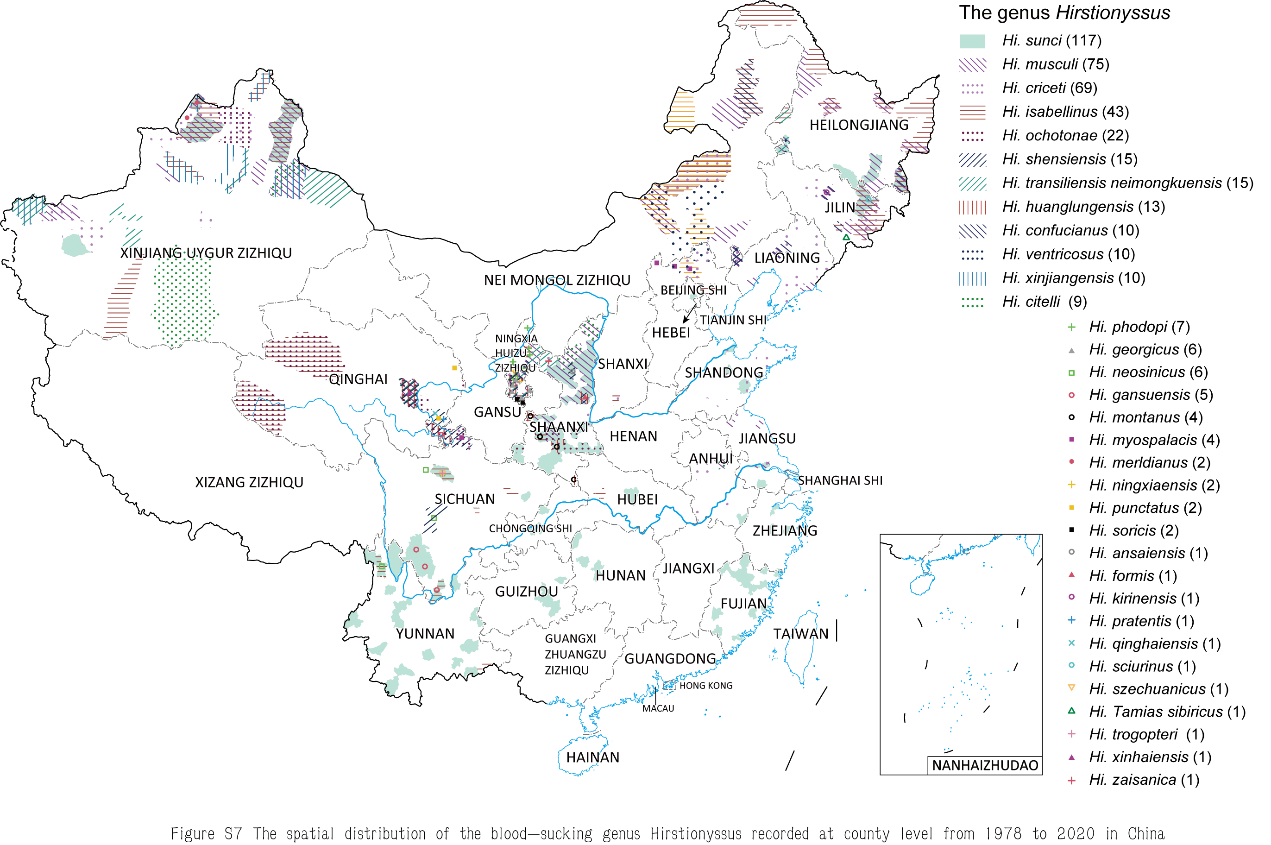


**Figure S8**: **The spatial distribution of the blood-sucking mite genus *Leptotrombidium* recorded at county level from 1978 to 2020 in China.**
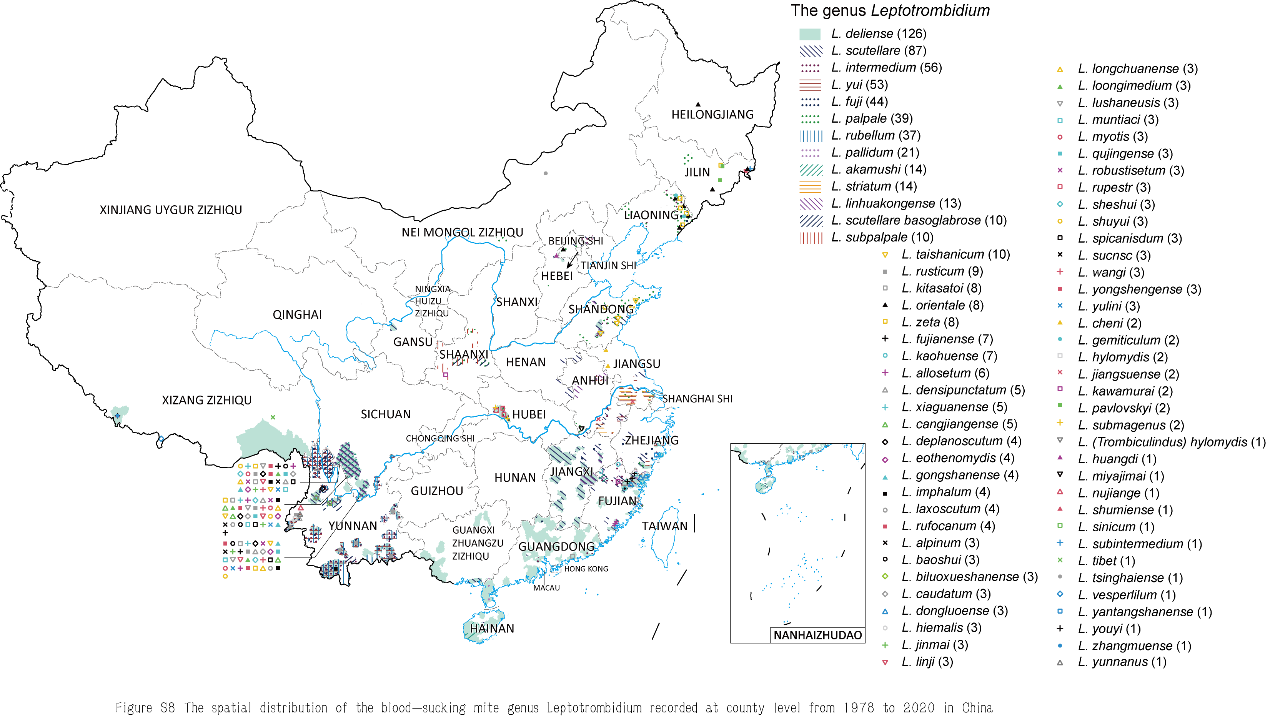


**Figure S9**: **The spatial distribution of the blood-sucking mite genus *Haemogamasus* recorded at county level from 1978 to 2020 in China.**

***
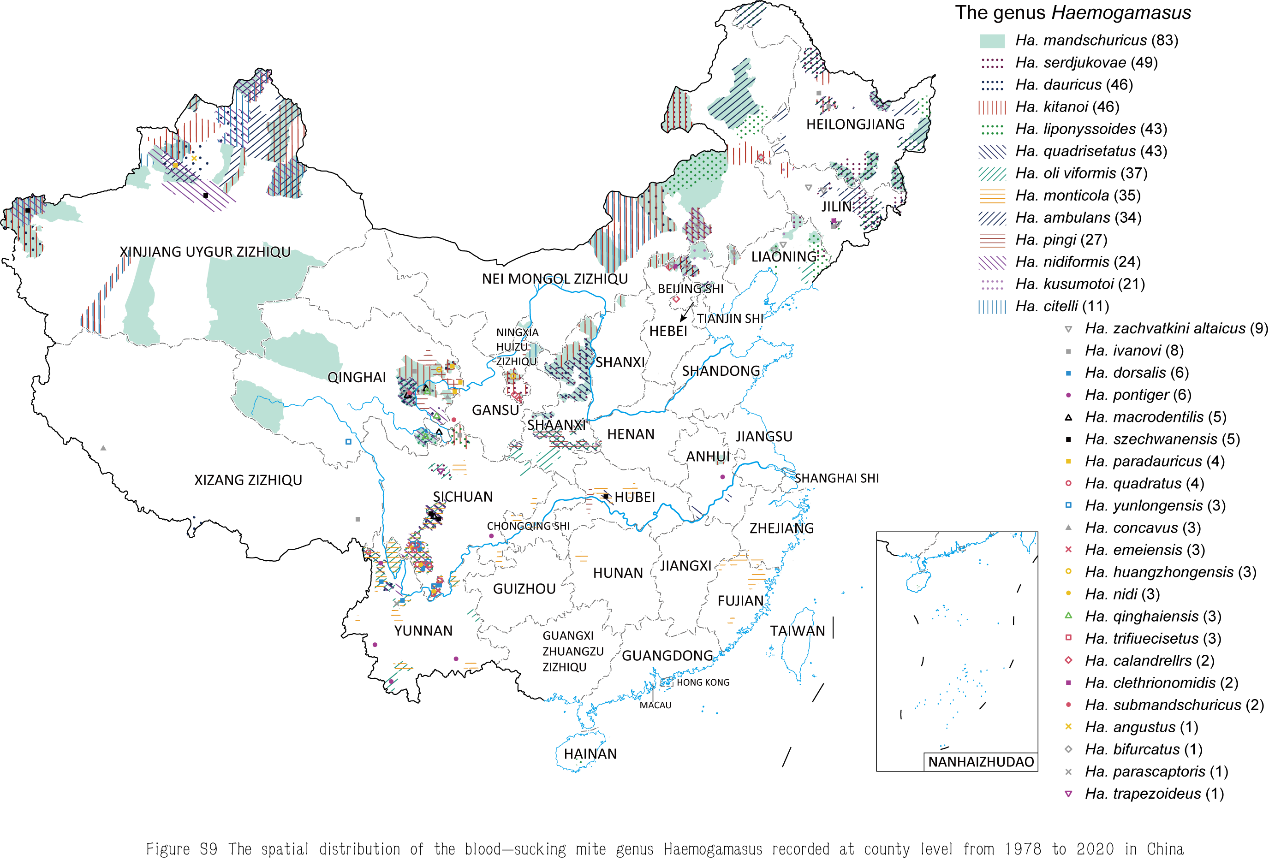
***

**Figure S10**: **The spatial distribution of the blood-sucking mite genus *Hypoaspis* recorded at county level from 1978 to 2020 in China.**

*
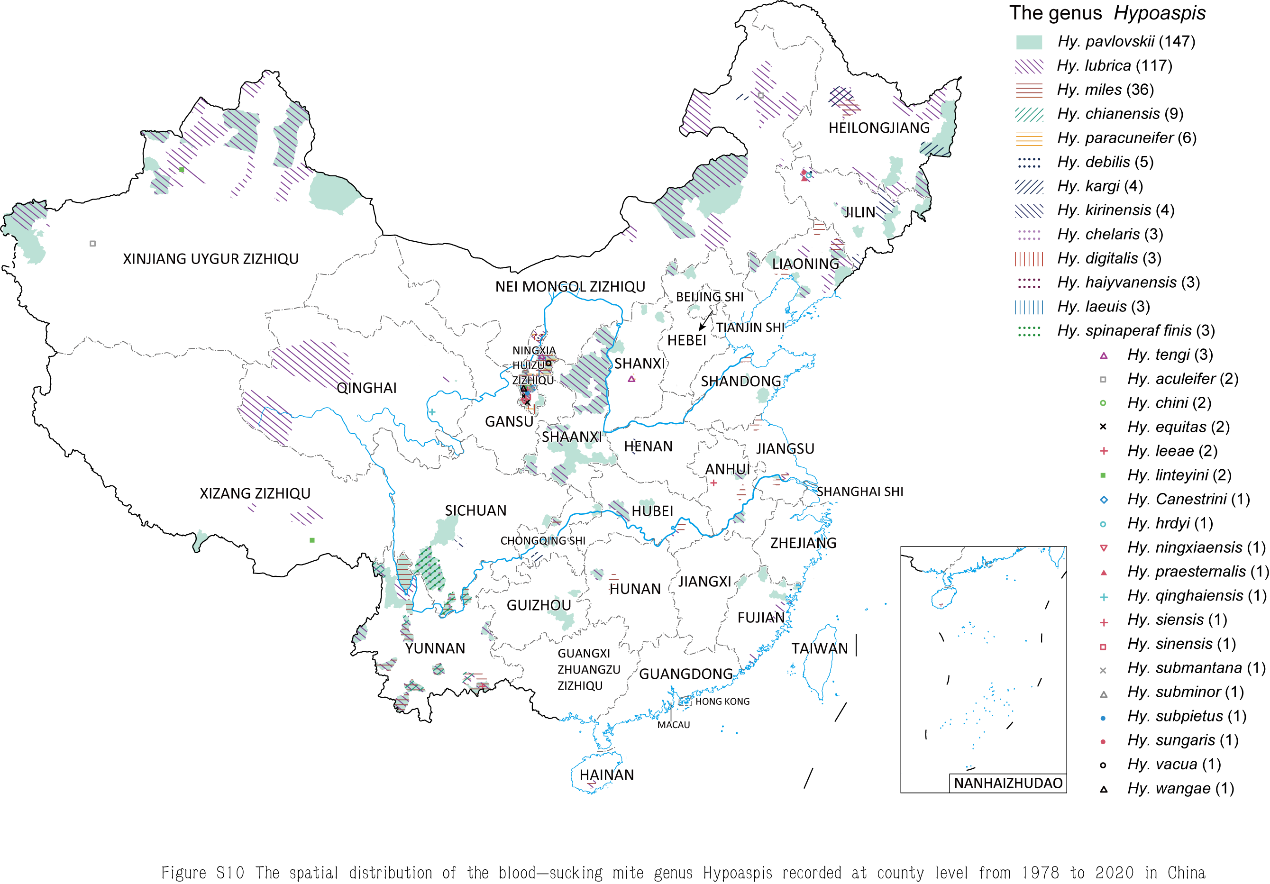
*

**Figure S11**: **The mean curves (red) and 95% percentiles (gray) for the effects of major predictors (RC≥5%) on the logit-transformed probability of occurrence of *L. yui* based on the ensemble of BRT models.** **Frequency distributions of the predictor is shown by the histograms in dark gray.**
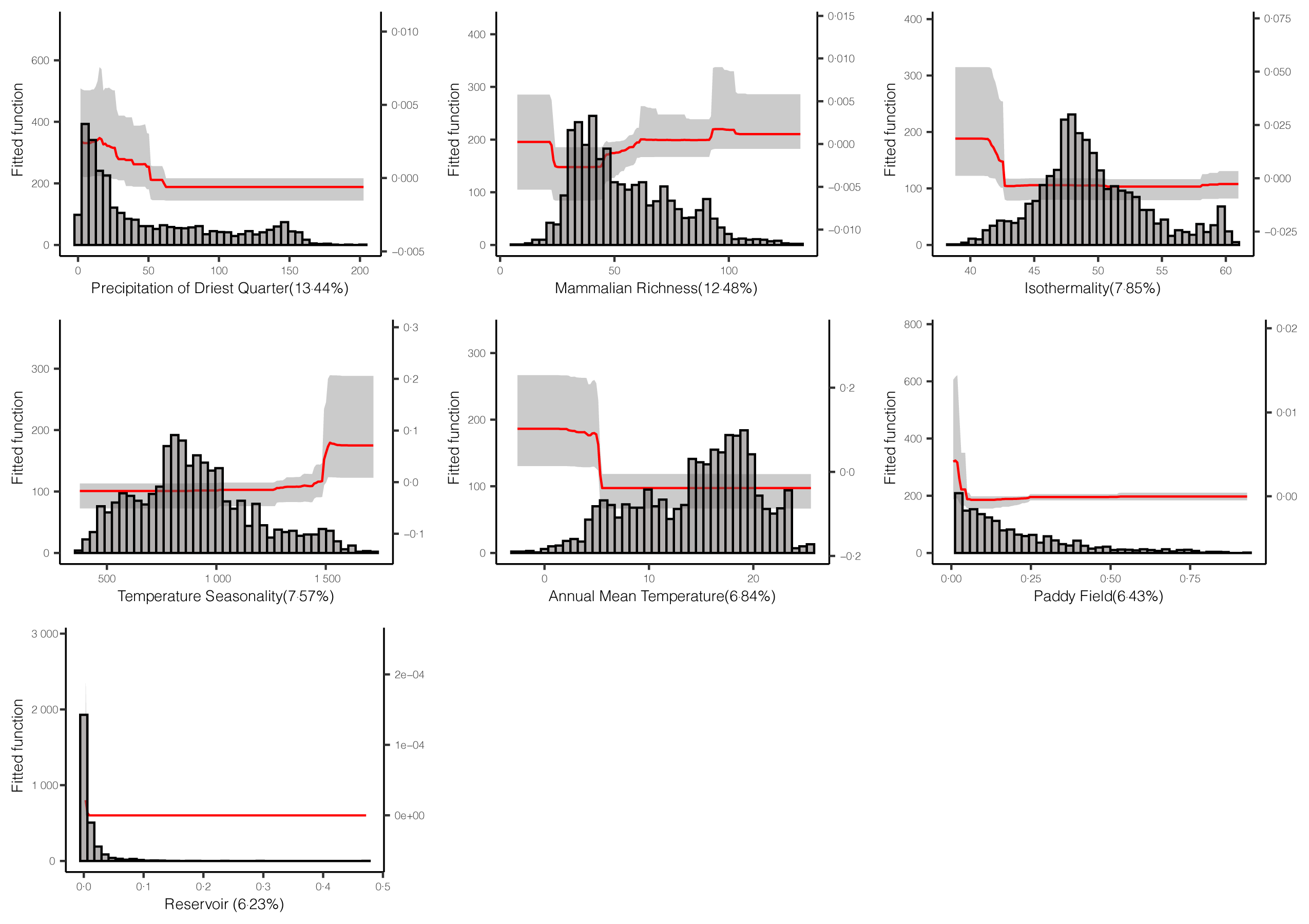


**Figure S12**: **The mean curves (red) and 95% percentiles (gray) for the effects of major predictors (RC≥5%) on the logit-transformed probability of occurrence of *L. scutellare* based on the ensemble of BRT models. Frequency distributions of the predictor is shown by the histograms in dark gray.**
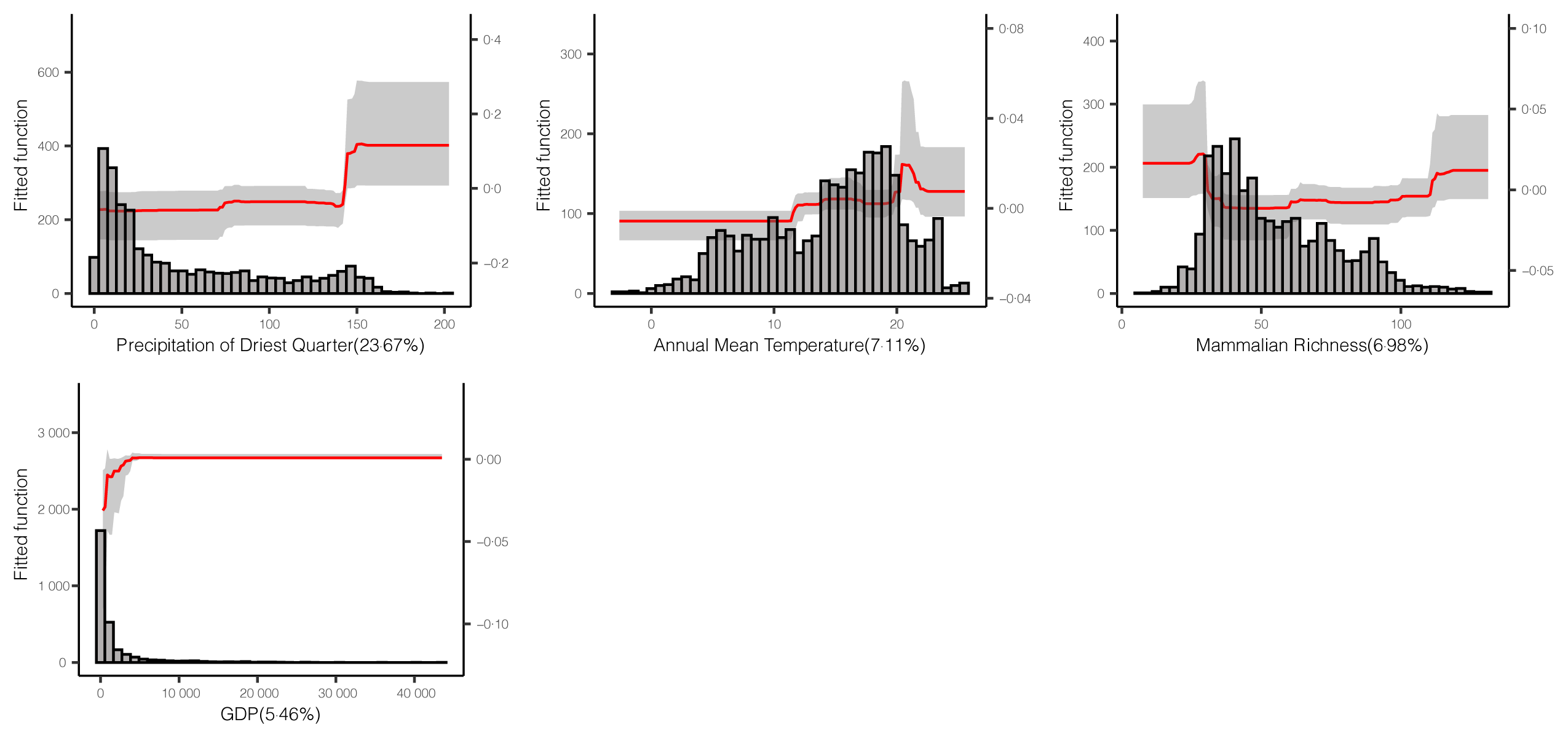


**Figure S13**: **The mean curves (red) and 95% percentiles (gray) for the effects of major predictors (RC≥5%) on the logit-transformed probability of occurrence of *Or. bacoti* based on the ensemble of BRT models. Frequency distributions of the predictor is shown by the histograms in dark gray.
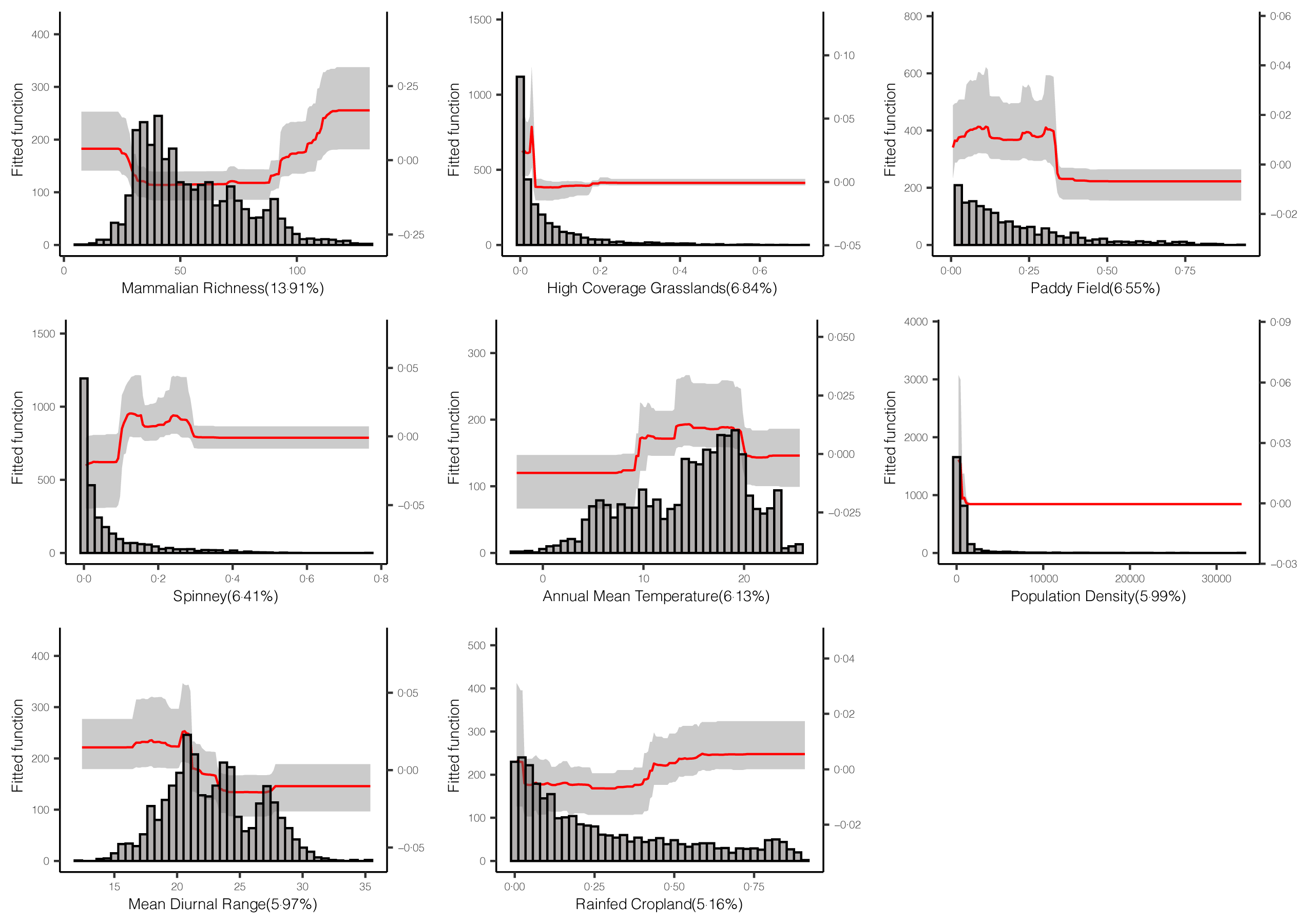
**

**Figure S14**: **on the logit-transformed probability of occurrence of *Od. majesticus* based on the ensemble of BRT models. Frequency distributions of the predictor is shown by the histograms in dark gray.**

**
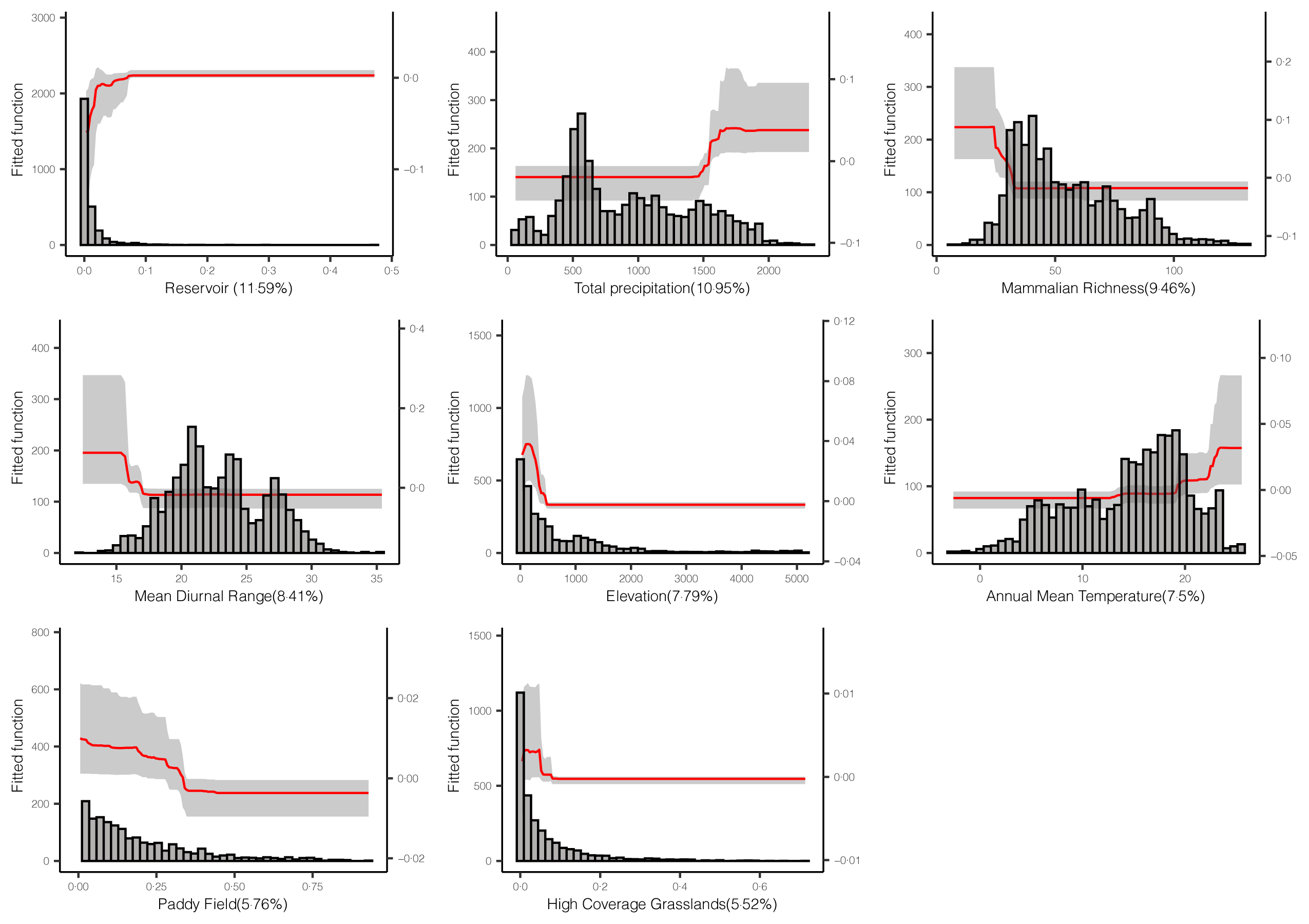
**

**Figure S15**: **The mean curves (red) and 95% percentiles (gray) for the effects of major predictors (RC≥5%) on the logit-transformed probability of occurrence of *L. deliense* based on the ensemble of BRT models. Frequency distributions of the predictor is shown by the histograms in dark gray.
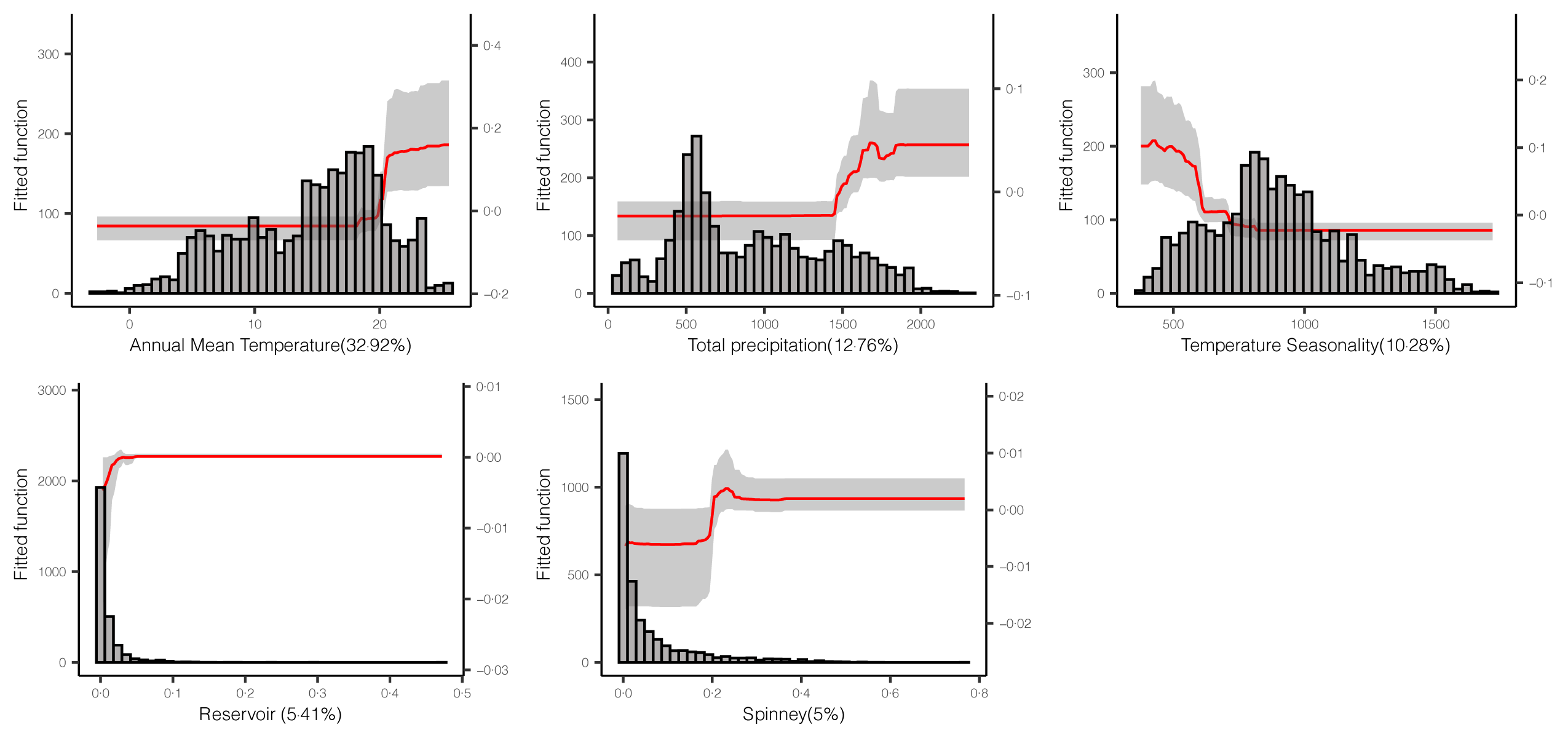
**

**Figure S16**: **The mean curves (red) and 95% percentiles (gray) for the effects of major predictors (RC≥5%) on the logit-transformed probability of occurrence of *L. intermedium* based on the ensemble of BRT models. Frequency distributions of the predictor is shown by the histograms in dark gray.
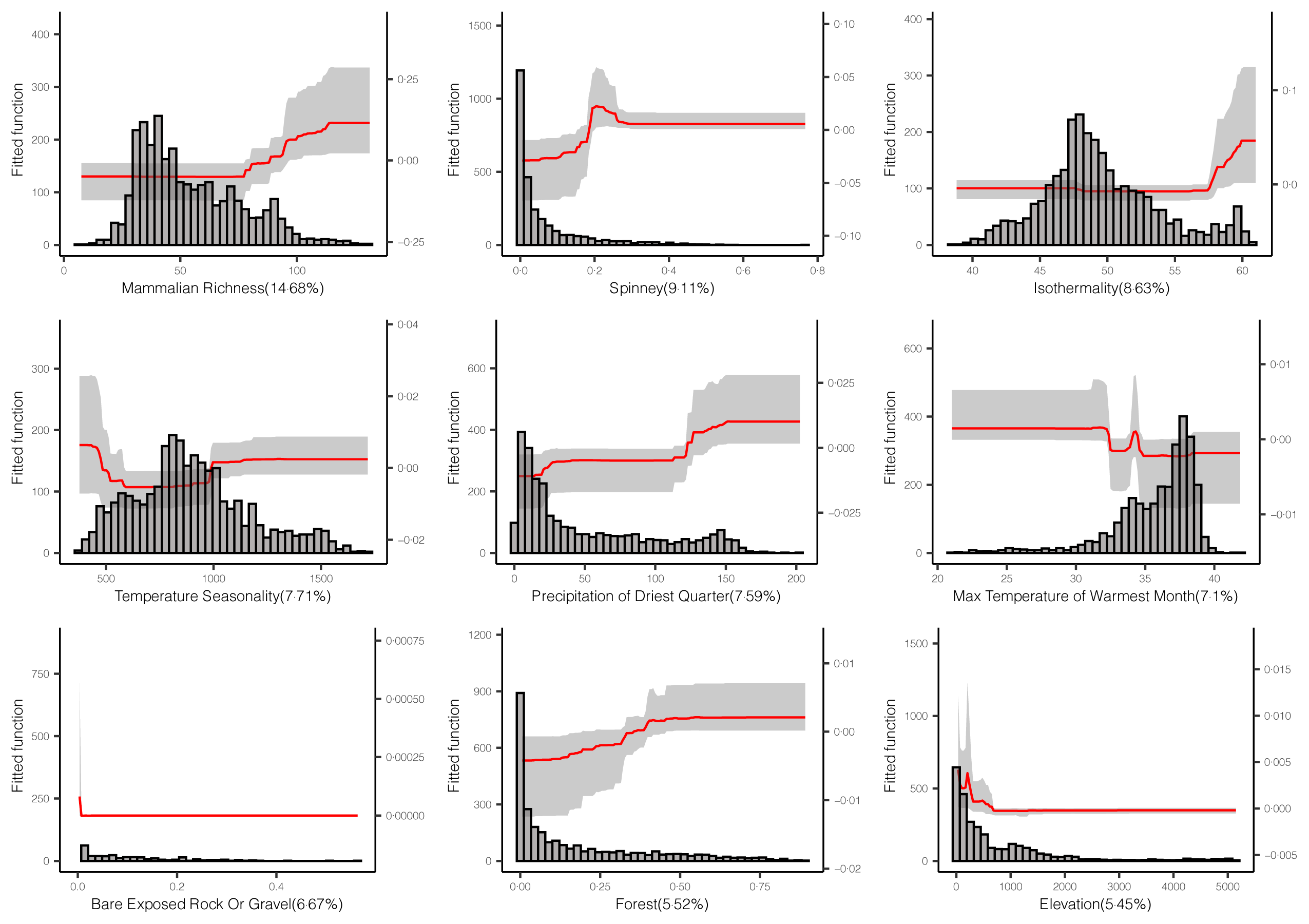
**

**Figure S17**: **The mean curves (red) and 95% percentiles (gray) for the effects of major predictors (RC≥5%) on the logit-transformed probability of occurrence of *L. fuji* based on the ensemble of BRT models. Frequency distributions of the predictor is shown by the histograms in dark gray.**

**
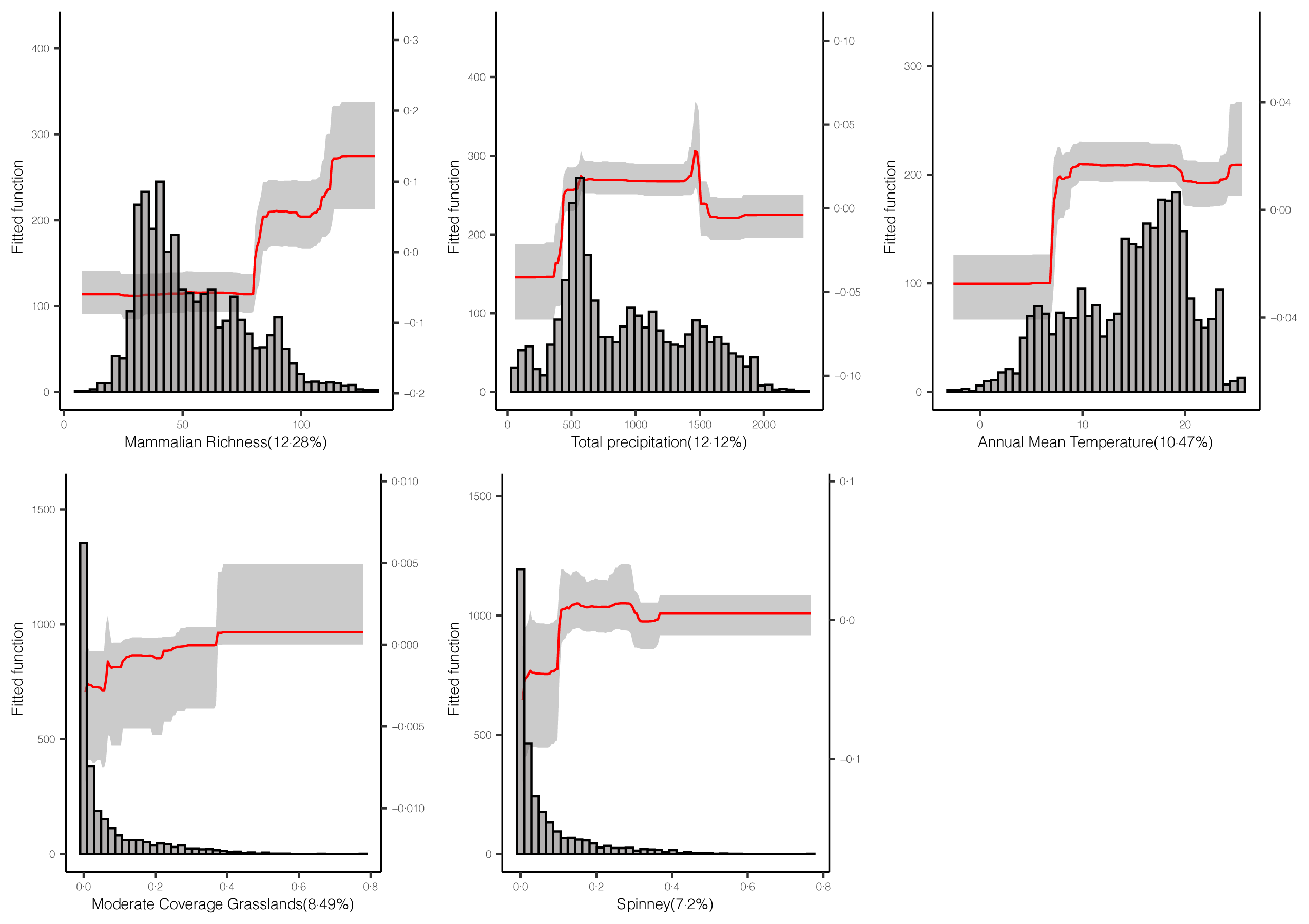
**

**Figure S18**: **The mean curves (red) and 95% percentiles (gray) for the effects of major predictors (RC≥5%) on the logit-transformed probability of occurrence of *L. rubellum* based on the ensemble of BRT models. Frequency distributions of the predictor is shown by the histograms in dark gray.
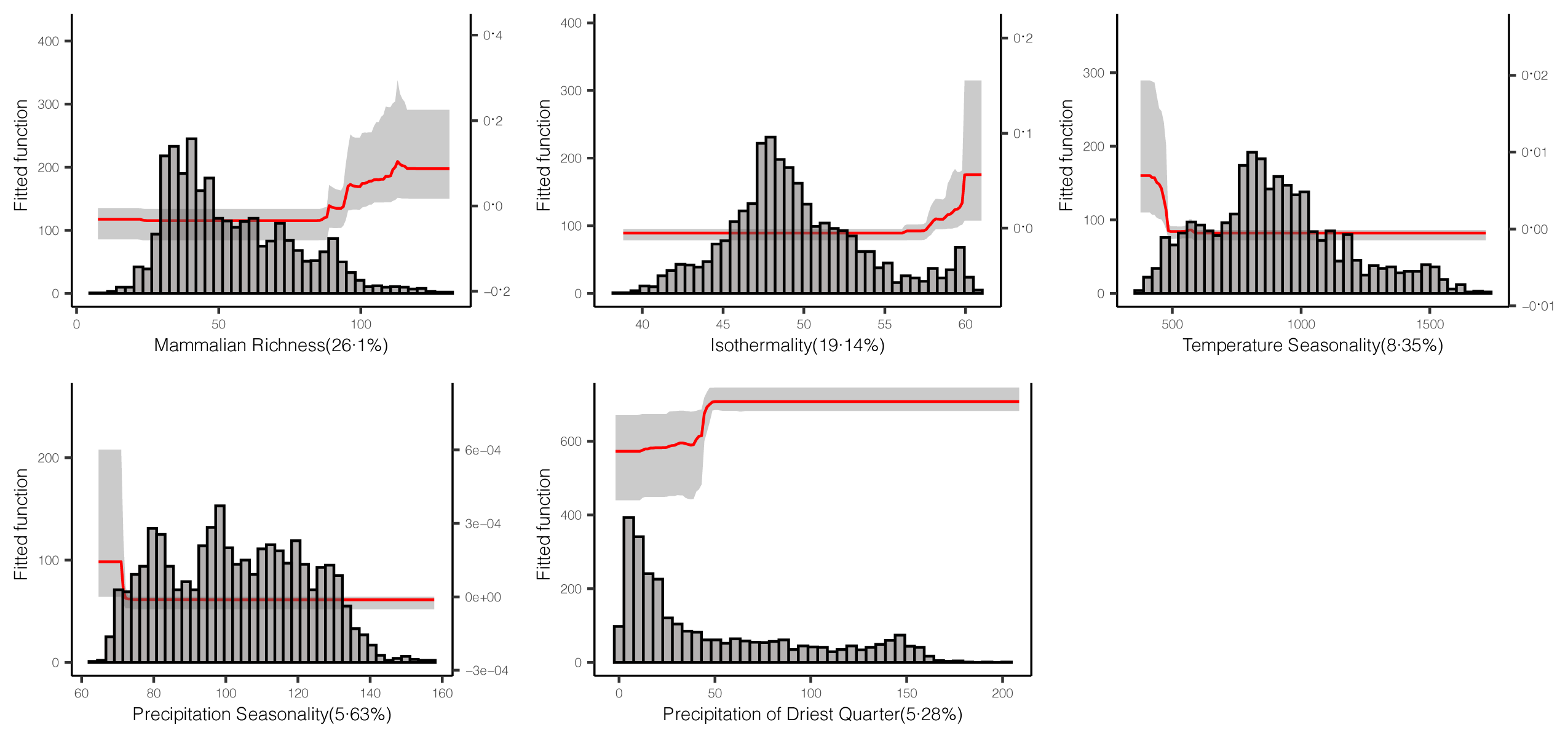
**

**Figure S19**: **The mean curves (red) and 95% percentiles (gray) for the effects of major predictors (RC≥5%) on the logit-transformed probability of occurrence of *As. indica* based on the ensemble of BRT models. Frequency distributions of the predictor is shown by the histograms in dark gray.
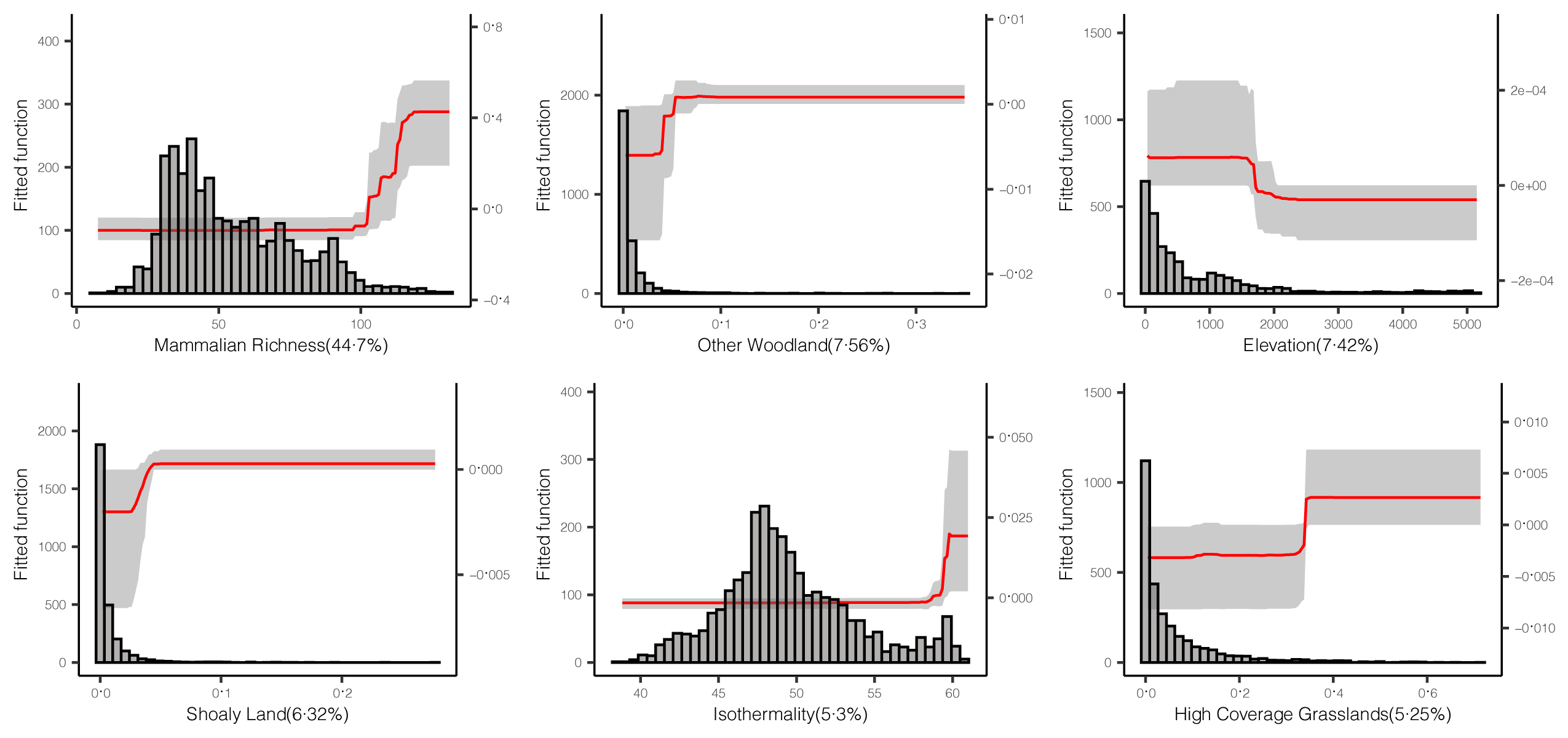
**

**Figure S20**: **The mean curves (red) and 95% percentiles (gray) for the effects of major predictors (RC≥5%) on the logit-transformed probability of occurrence of *Tr. myonysognathus* based on the ensemble of BRT models. Frequency distributions of the predictor is shown by the histograms in dark gray.
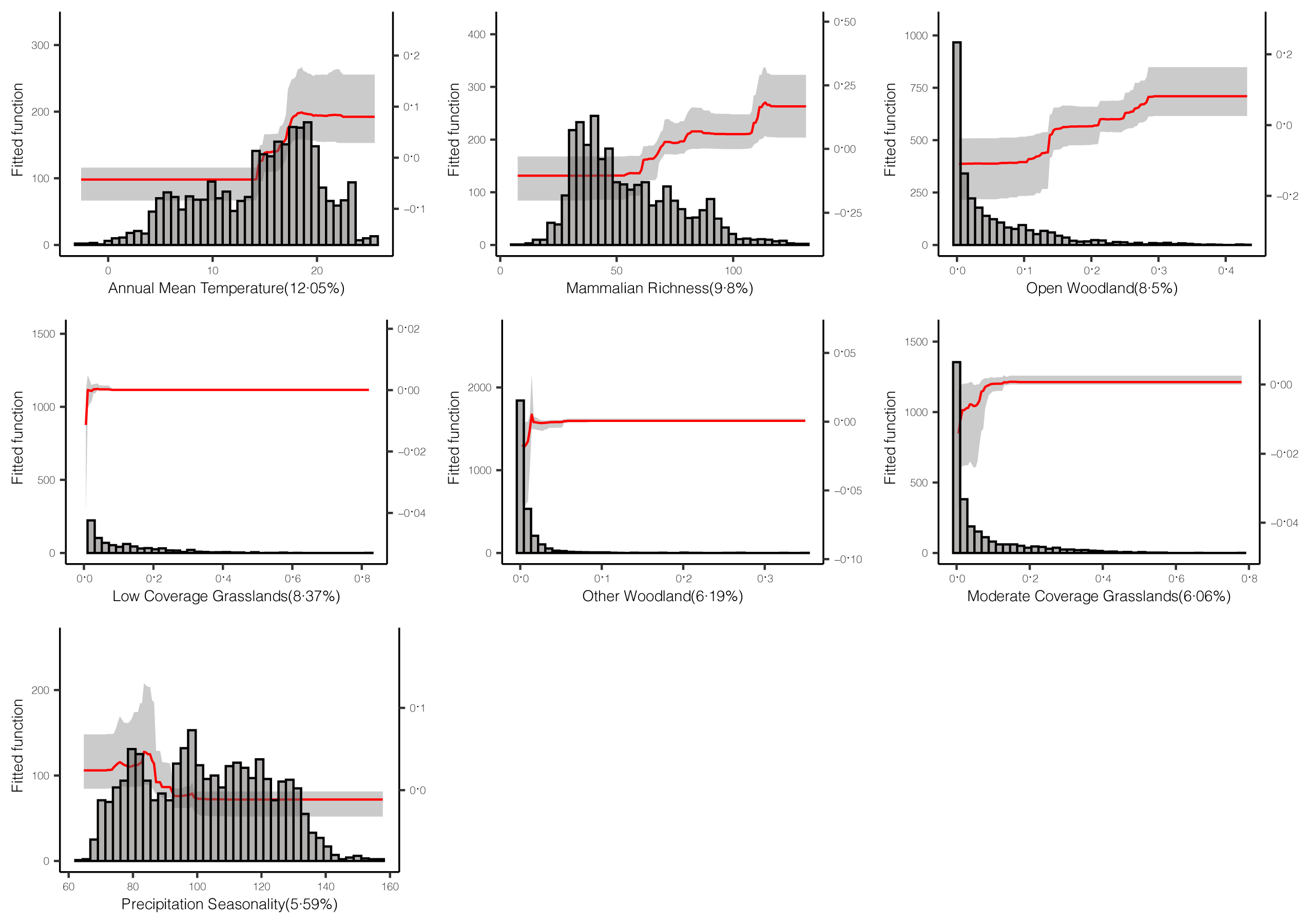
**

**Figure S21**: **The mean curves (red) and 95% percentiles (gray) for the effects of major predictors (RC≥5%) on the logit-transformed probability of occurrence of *La. nuttalli* based on the ensemble of BRT models. Frequency distributions of the predictor is shown by the histograms in dark gray.
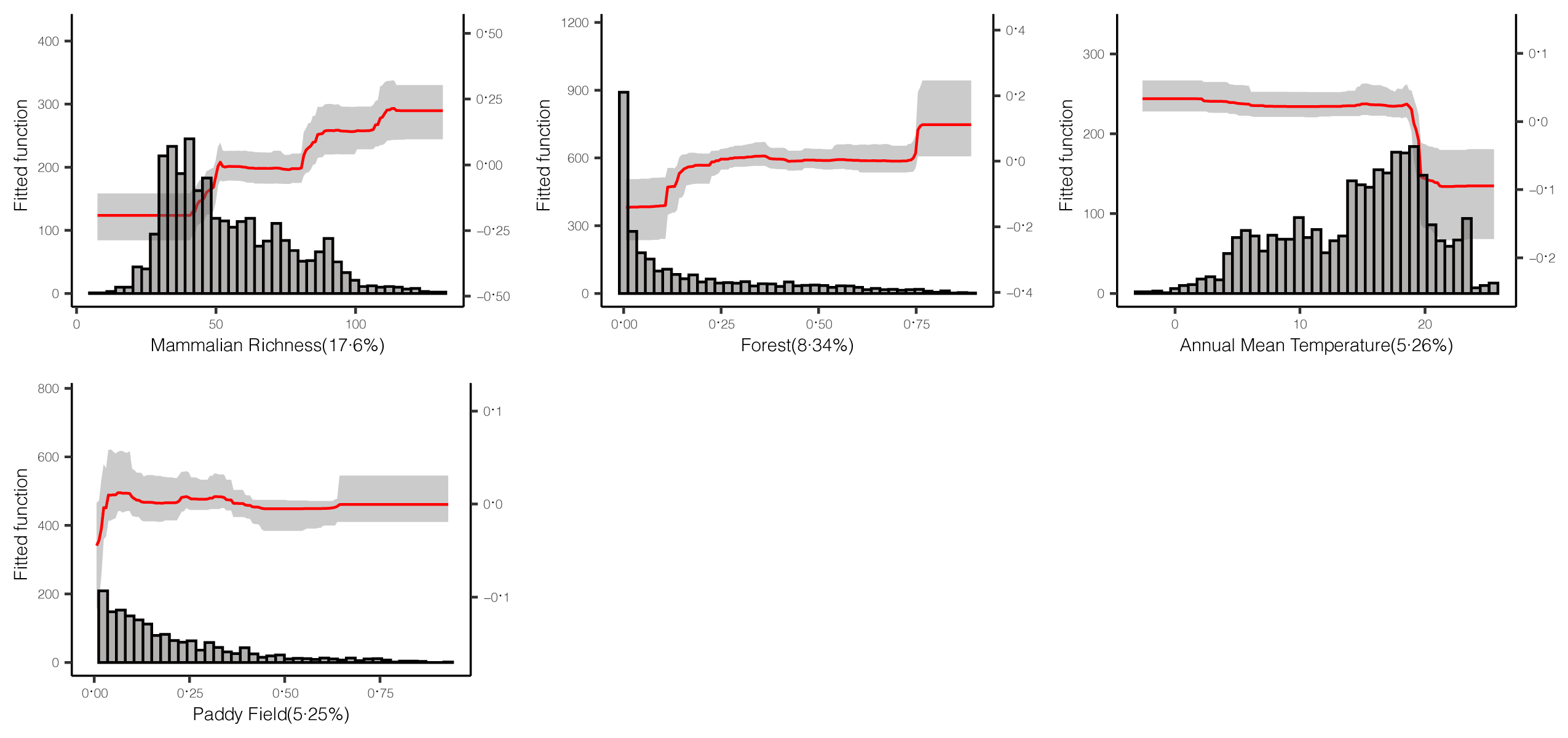
**

**Figure S22**: **The mean curves (red) and 95% percentiles (gray) for the effects of major predictors (RC≥5%) on the logit-transformed probability of occurrence of *Hy. lubrica* based on the ensemble of BRT models. Frequency distributions of the predictor is shown by the histograms in dark gray.
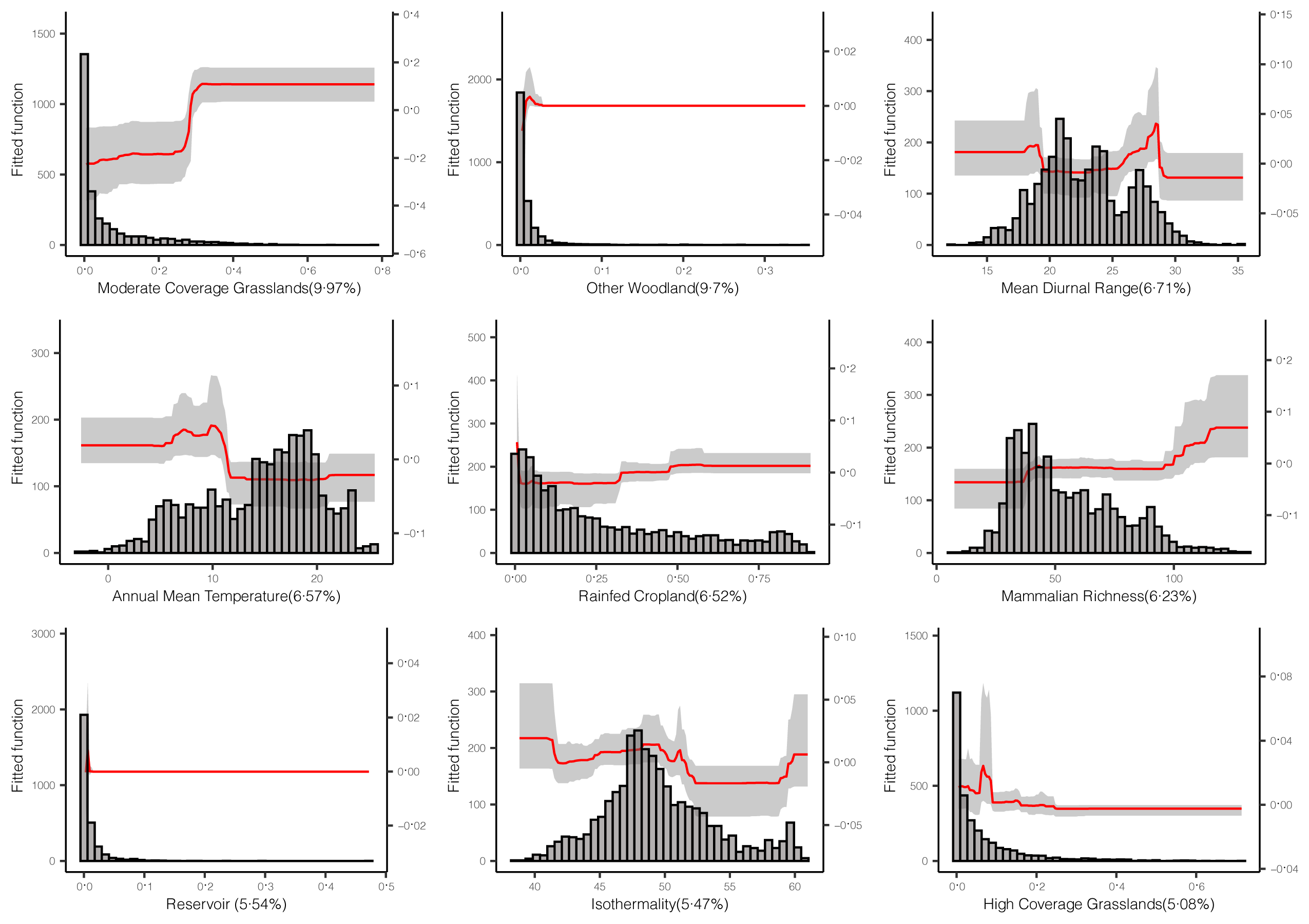
**

**Figure S23**: **The mean curves (red) and 95% percentiles (gray) for the effects of major predictors (RC≥5%) on the logit-transformed probability of occurrence of *Hi. isabellinus* based on the ensemble of BRT models. Frequency distributions of the predictor is shown by the histograms in dark gray.
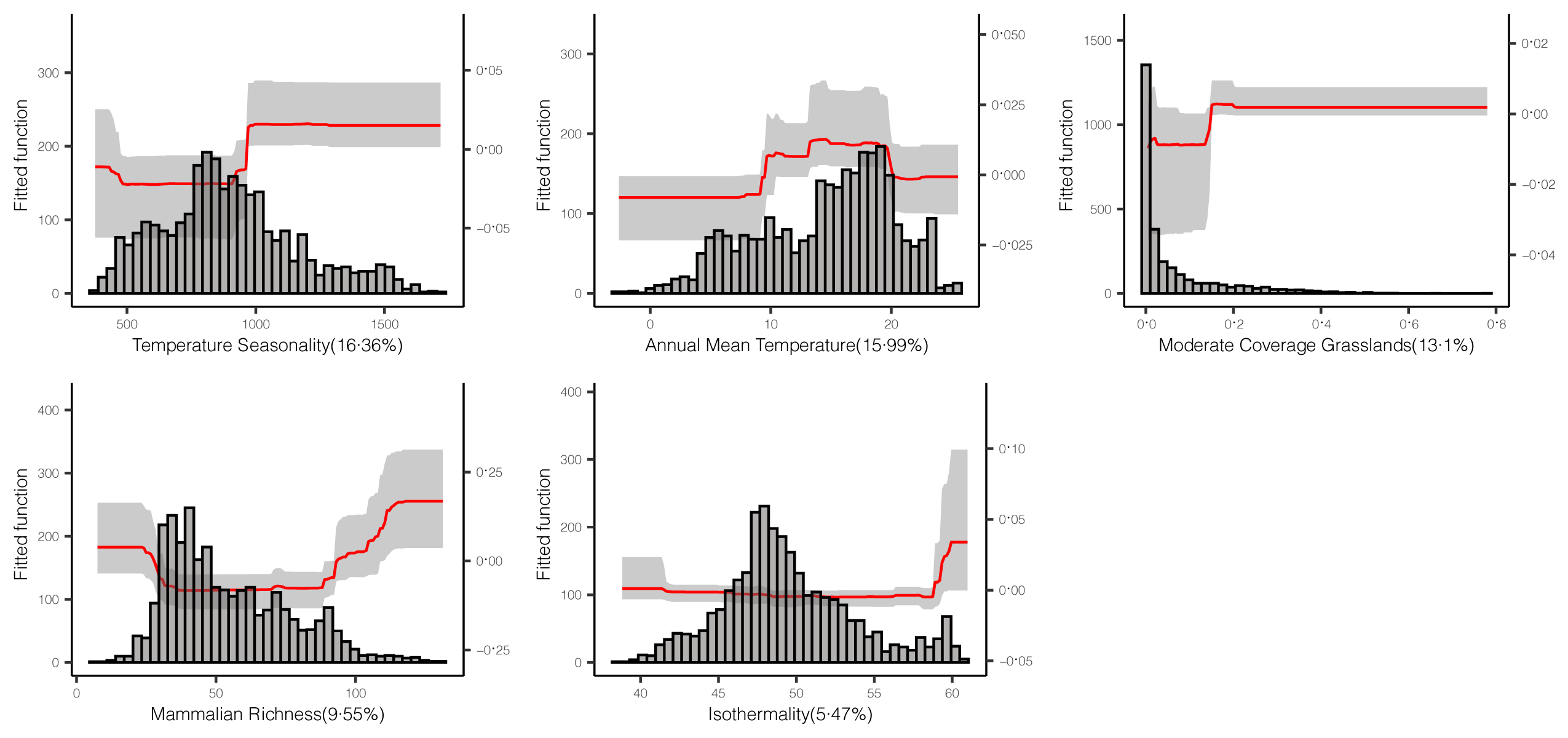
**

**Figure S24**: **The mean curves (red) and 95% percentiles (gray) for the effects of major predictors (RC≥5%) on the logit-transformed probability of occurrence of *Ha. glasgowf* based on the ensemble of BRT models. Frequency distributions of the predictor is shown by the histograms in dark gray.
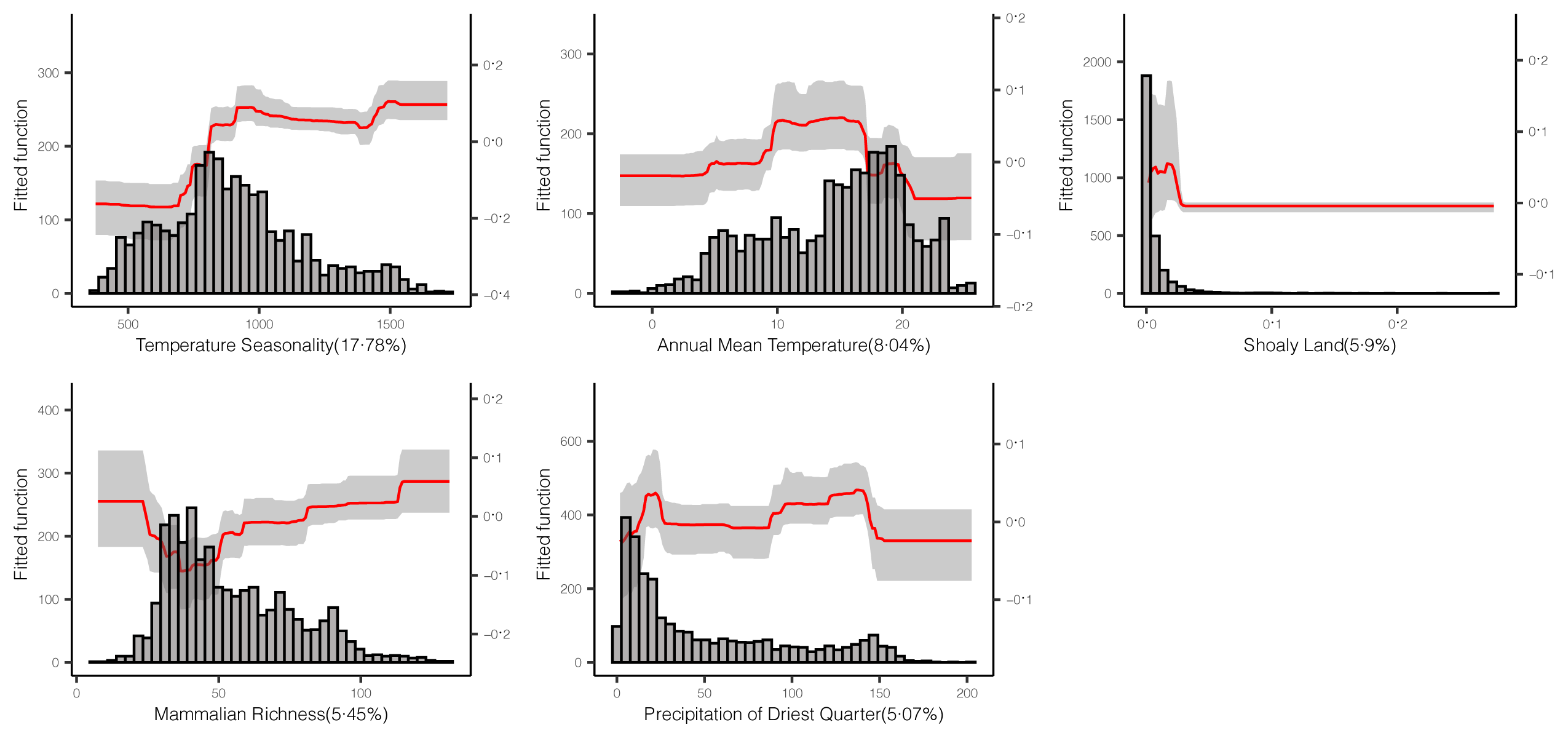
**

**Figure S25**: **The mean curves (red) and 95% percentiles (gray) for the effects of major predictors (RC≥5%) on the logit-transformed probability of occurrence of *La. jettmari* based on the ensemble of BRT models. Frequency distributions of the predictor is shown by the histograms in dark gray.
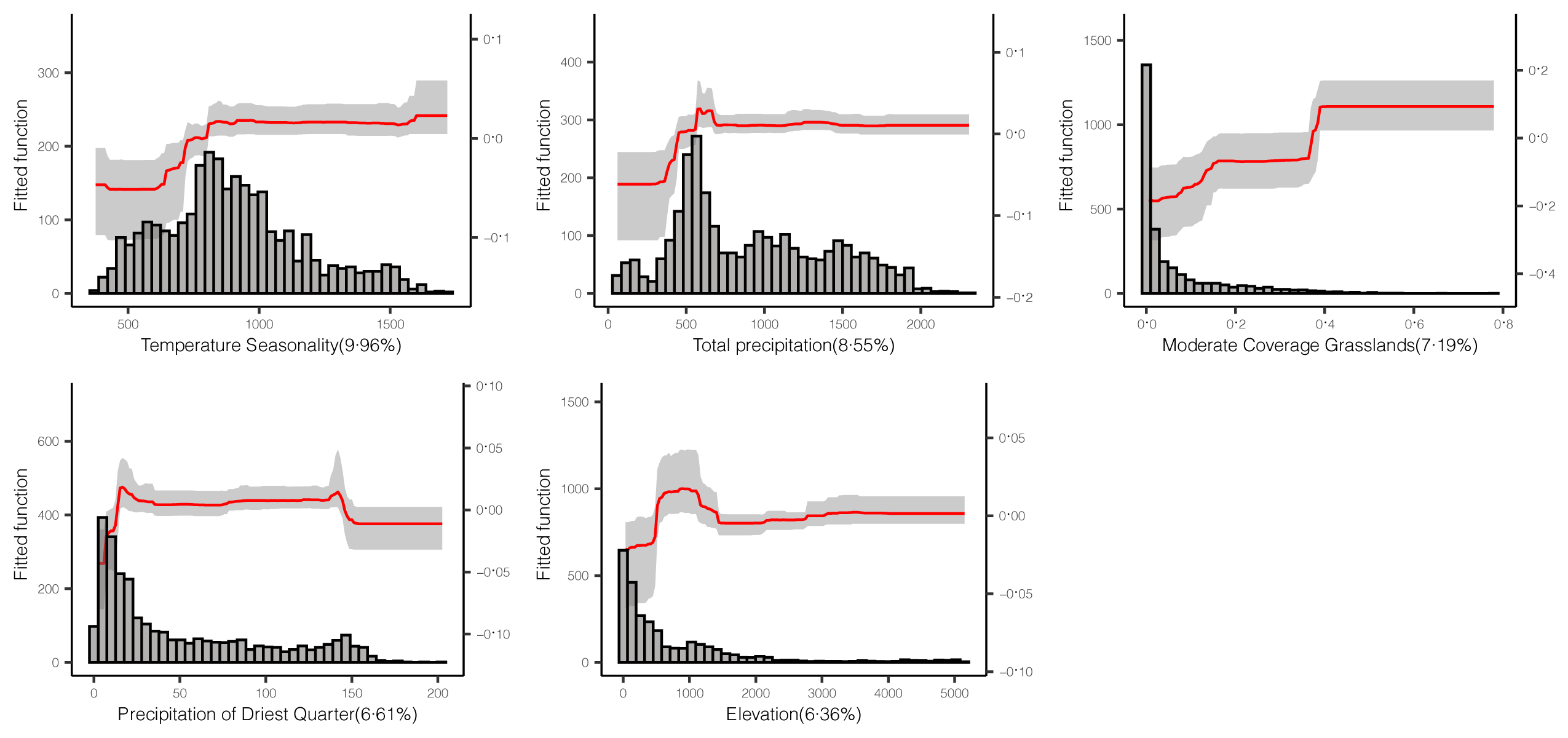
**

**Figure S26**: **The mean curves (red) and 95% percentiles (gray) for the effects of major predictors (RC≥5%) on the logit-transformed probability of occurrence of *Eu. stabularis* based on the ensemble of BRT models. Frequency distributions of the predictor is shown by the histograms in dark gray.**

**
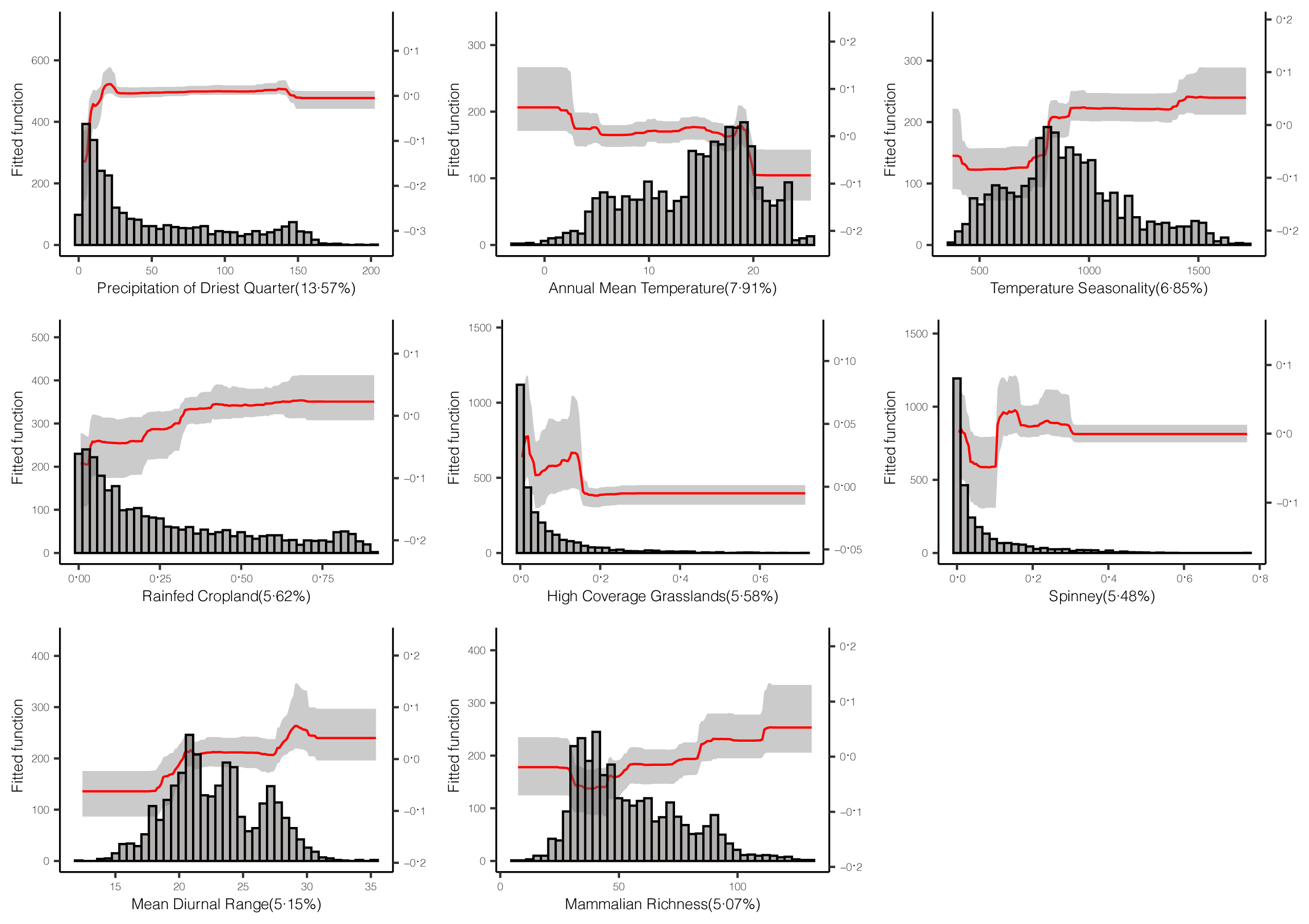
**

**Figure S27**: **The mean curves (red) and 95% percentiles (gray) for the effects of major predictors (RC≥5%) on the logit-transformed probability of occurrence of *Hi. sunci* based on the ensemble of BRT models. Frequency distributions of the predictor is shown by the histograms in dark gray.
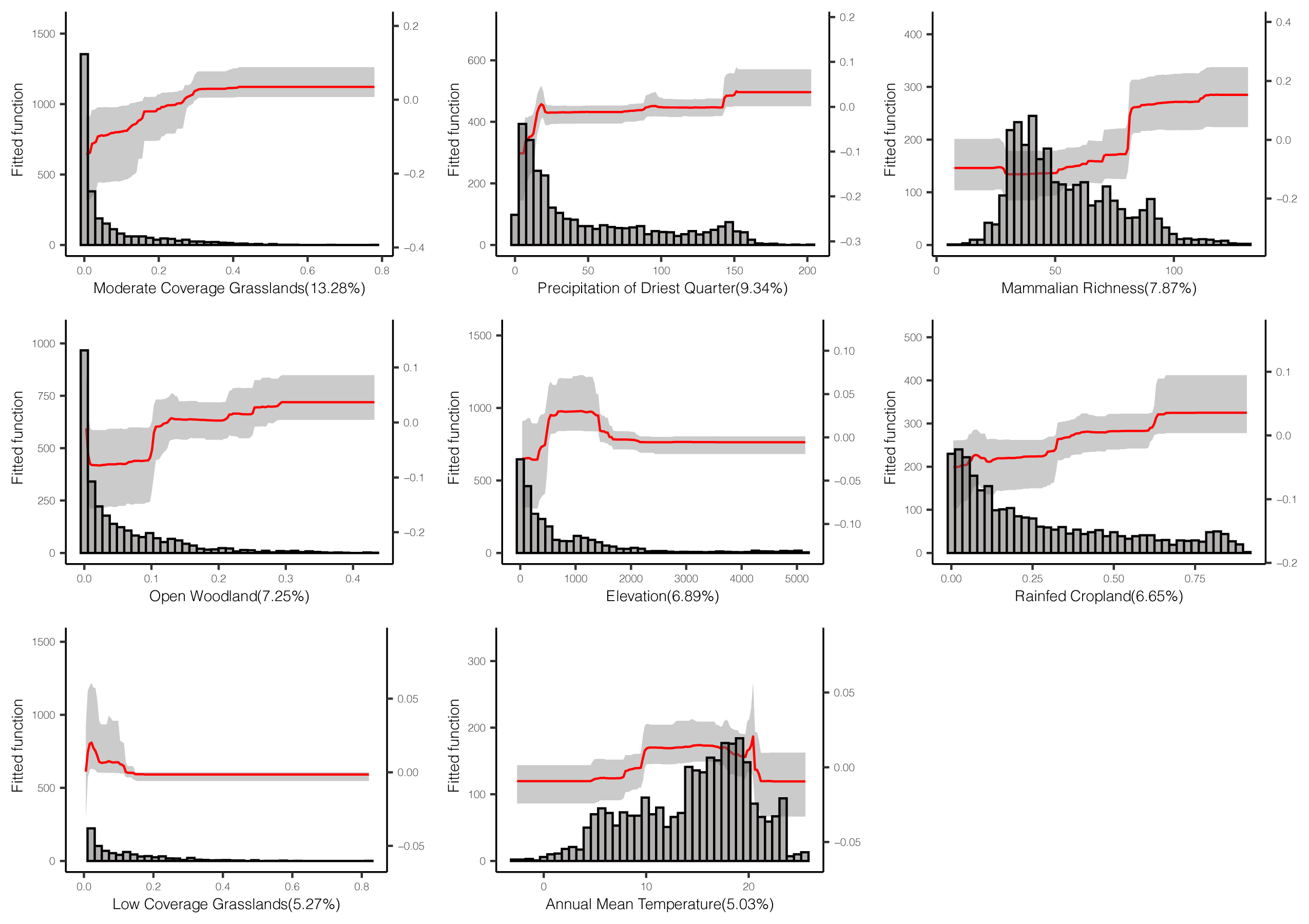
**

**Figure S28**: **The mean curves (red) and 95% percentiles (gray) for the effects of major predictors (RC≥5%) on the logit-transformed probability of occurrence of *La. echidninus* based on the ensemble of BRT models. Frequency distributions of the predictor is shown by the histograms in dark gray.
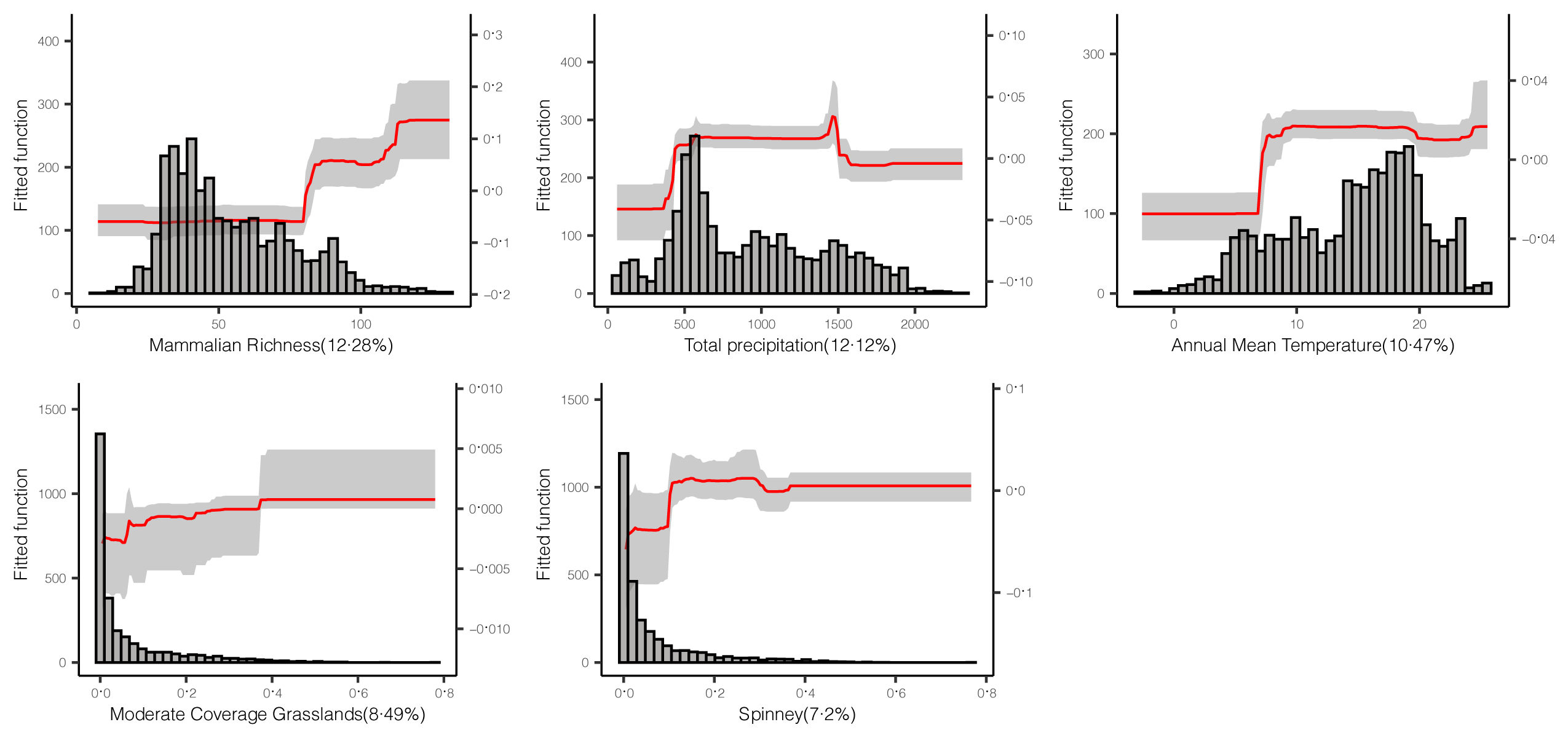
**

**Figure S29**: **The mean curves (red) and 95% percentiles (gray) for the effects of major predictors (RC≥5%) on the logit-transformed probability of occurrence of *Eu. shanghaiensis* based on the ensemble of BRT models. Frequency distributions of the predictor is shown by the histograms in dark gray.
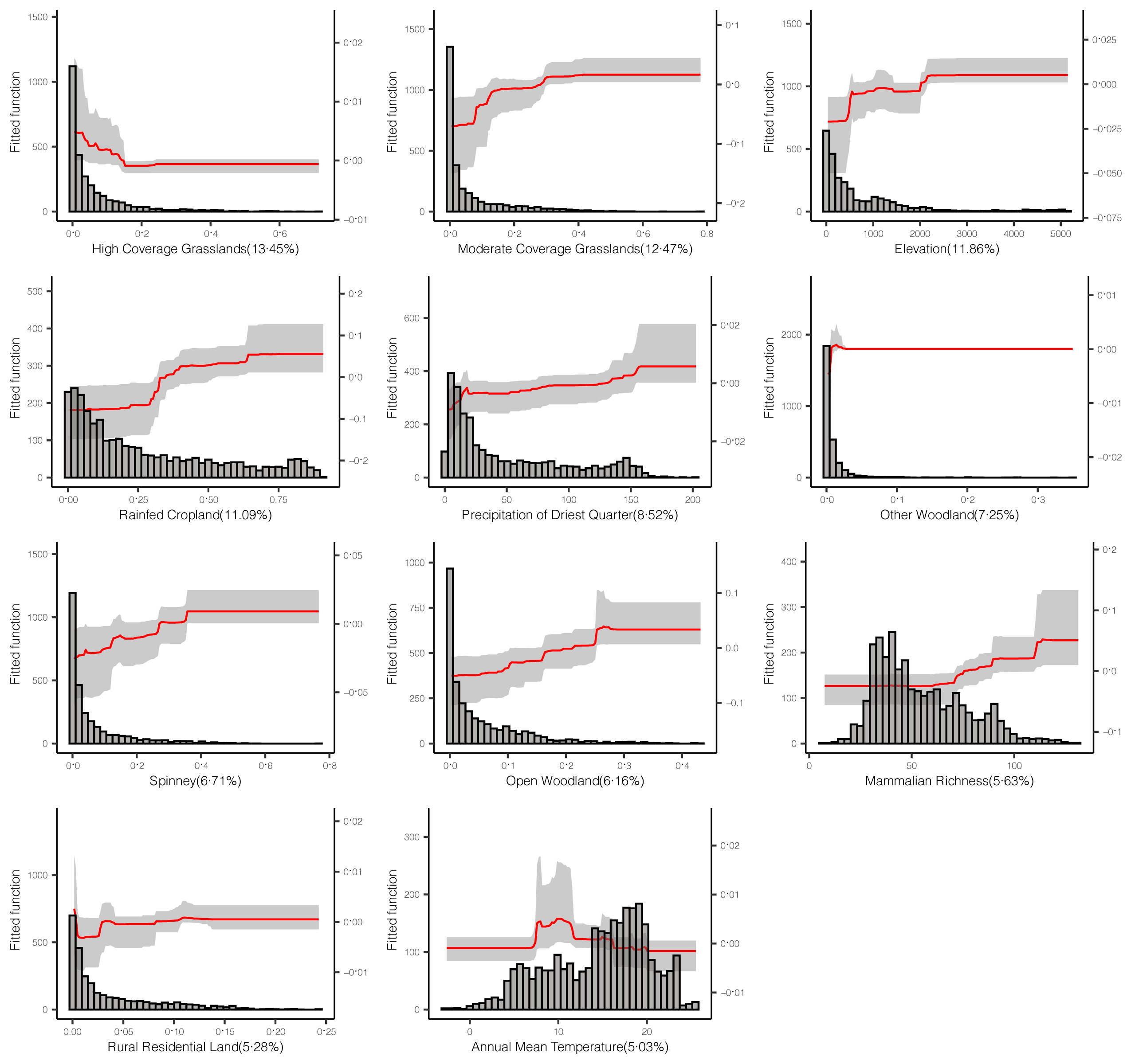
**

**Figure S30**: **The mean curves (red) and 95% percentiles (gray) for the effects of major predictors (RC≥5%) on the logit-transformed probability of occurrence of *Hy. pavlovskii* based on the ensemble of BRT models. Frequency distributions of the predictor is shown by the histograms in dark gray.
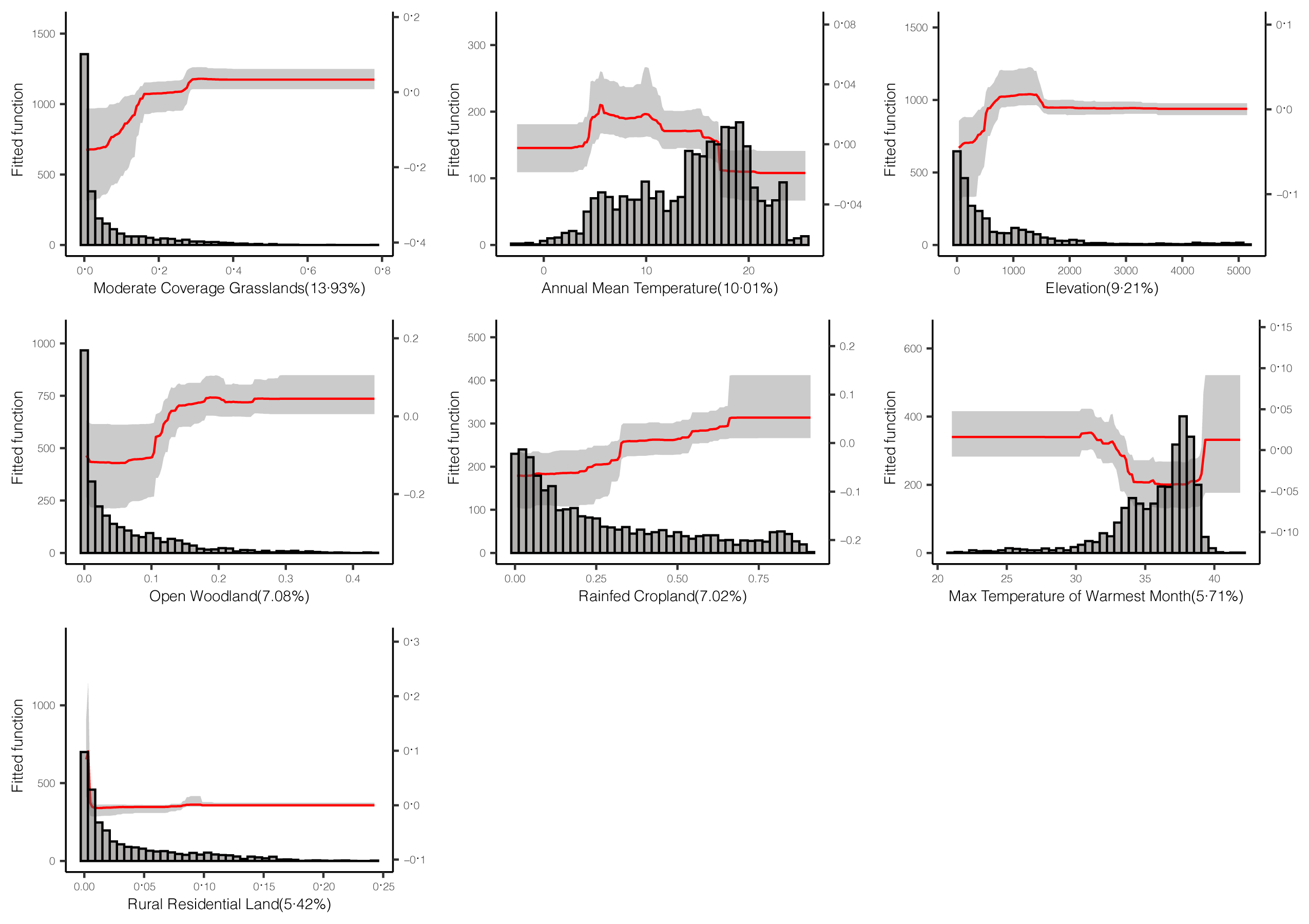
**

**Figure S31**: **The mean curves (red) and 95% percentiles (gray) for the effects of major predictors (RC≥5%) on the logit-transformed probability of occurrence of *L. palpale* based on the ensemble of BRT models. Frequency distributions of the predictor is shown by the histograms in dark gray.**

**
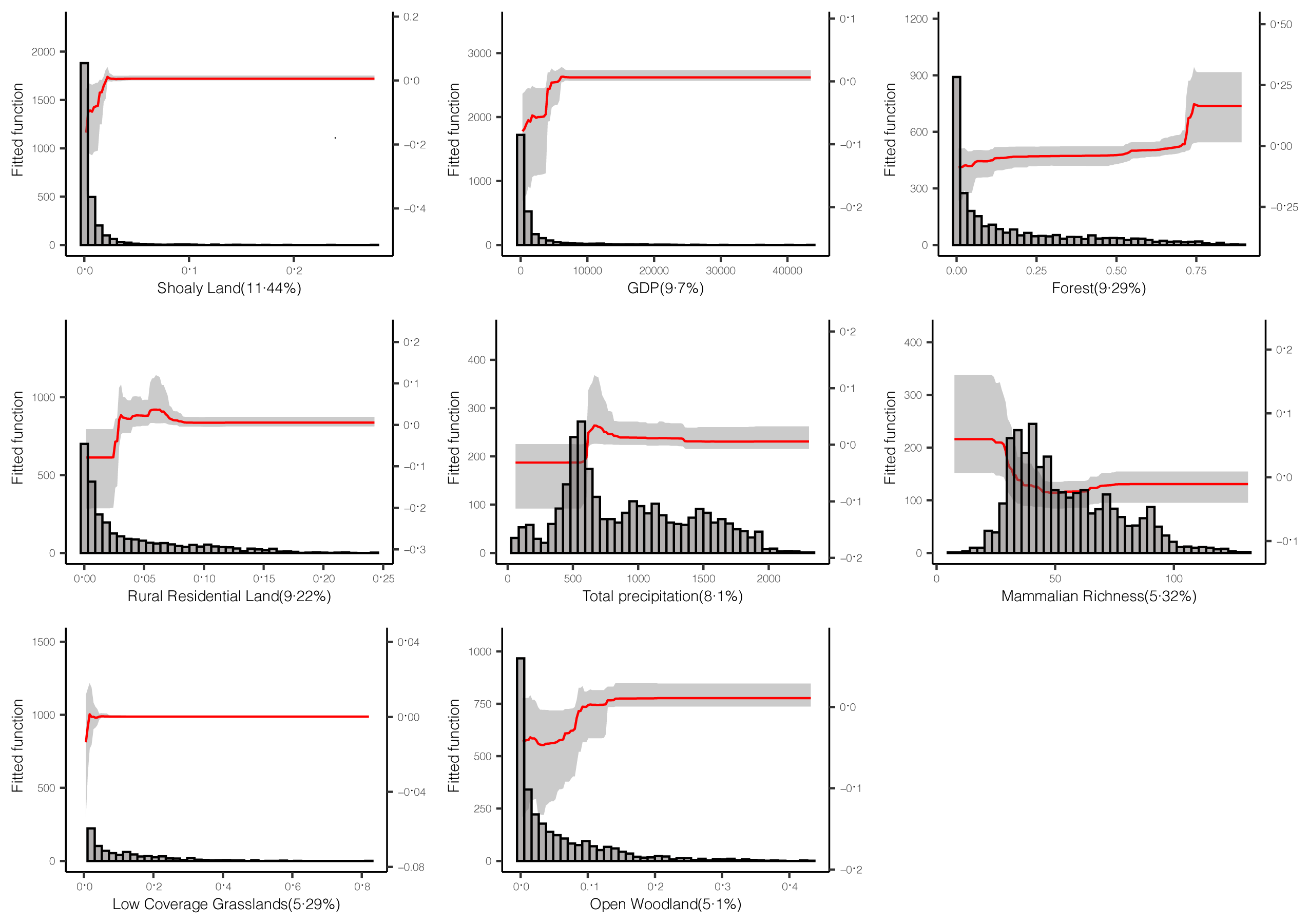
**

**Figure S32**: **The predicted county-level distributions of the Cluster Ⅰ, averaged over the ensemble of BRT models (a) *L. yui,* (b) *L. scutellare,* (c) *Or. bacoti,* (d) *Od. majesticus and* (e) *L. deliense*.**

**
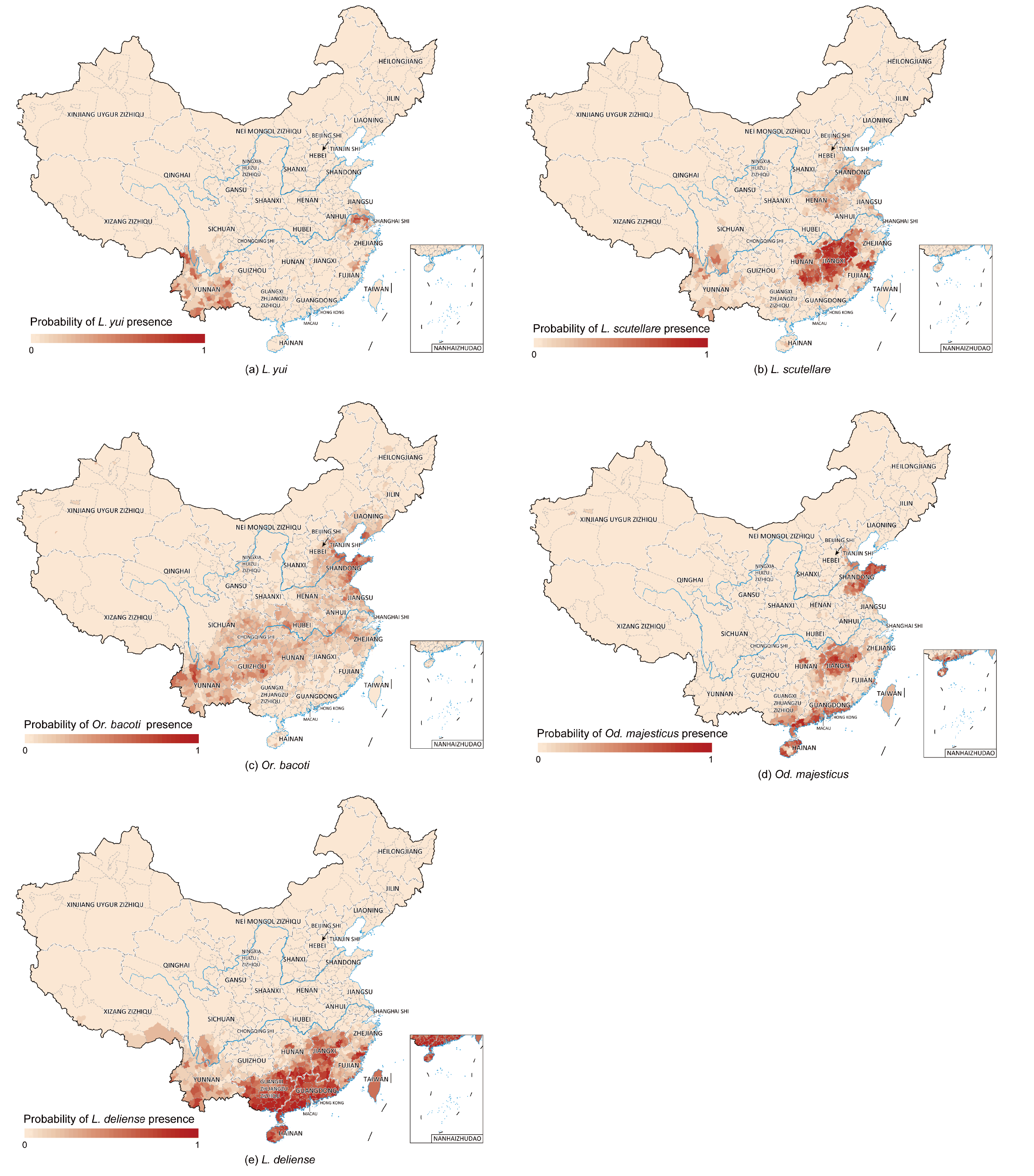
**

**Figure S33**: **The predicted county-level distributions of the Cluster Ⅱ, averaged over the ensemble of BRT models (a) *L. intermedium,* (b) *L. fuji*, (c) *L. rubellum*, (d) *As. indica,* (e) *Tr. myonysognathus and* (f) *La. nuttalli*.**

**
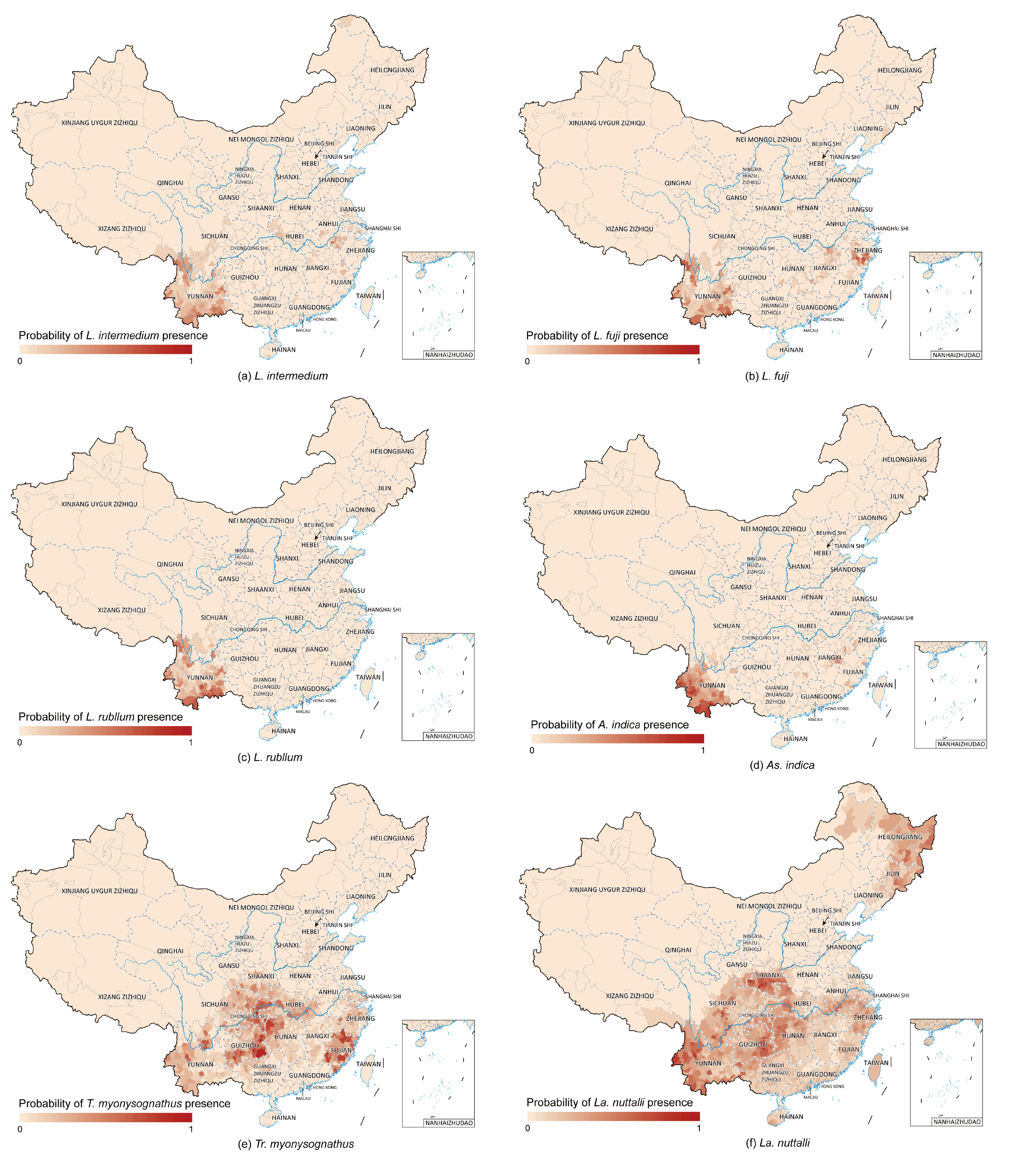
**

**Figure S34**: **The predicted county-level distributions of Cluster Ⅲ, averaged over the ensemble of BRT models (a) *Hy. lubrica,* (b) *Hi. isabellinus,* (c) *Ha. glasgowf,* (d) *La. jettmari and* (e) *Eu. stabularis*.**

**
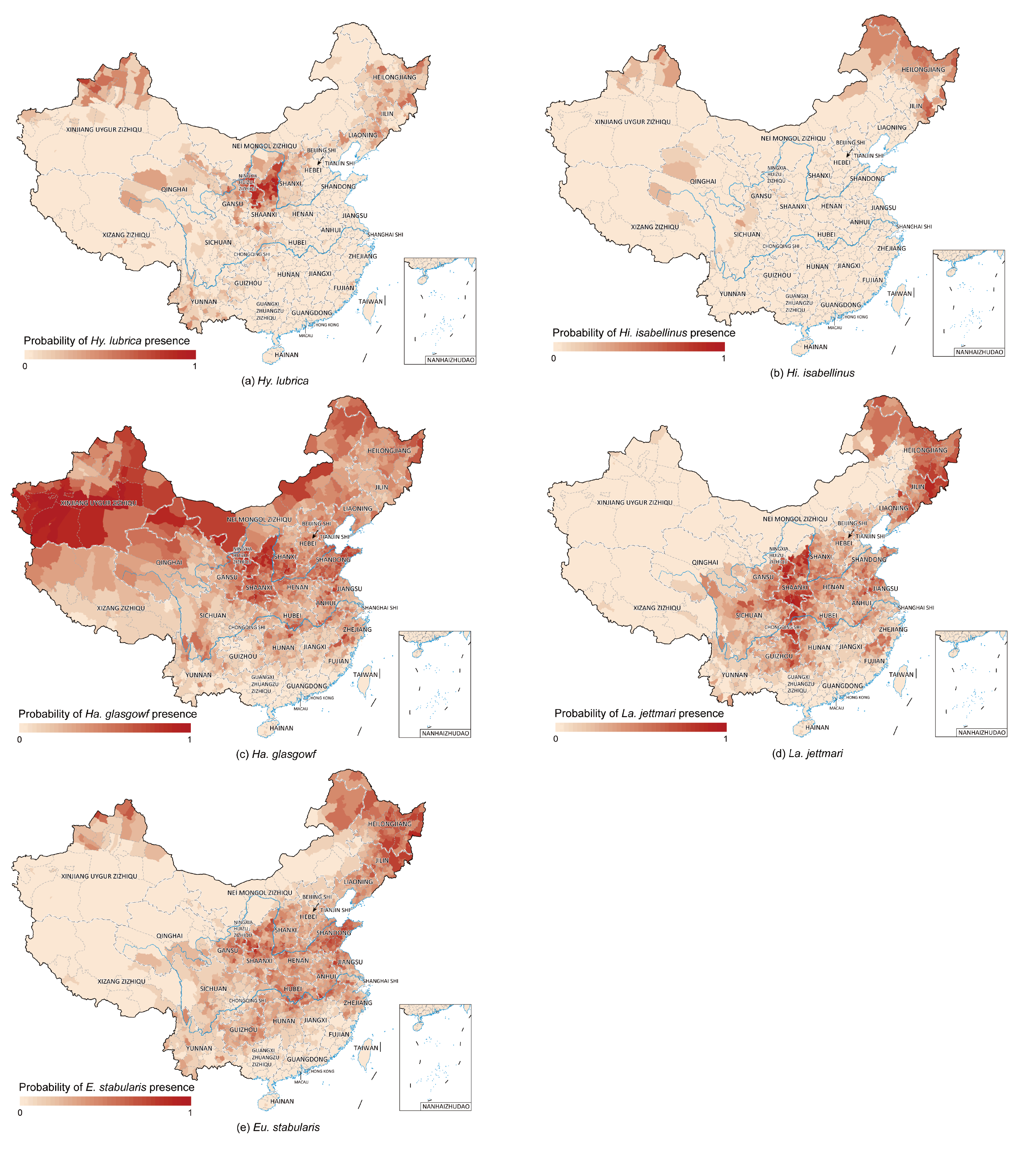
**

**Figure S35**: **The predicted county-level distributions of the Cluster Ⅳ, averaged over the ensemble of BRT models**: **(a) *Hi. sunci,* (b) *La. echidninus,* (c) *Eu. shanghaiensis and* (d) *Hy. pavlovskii*.
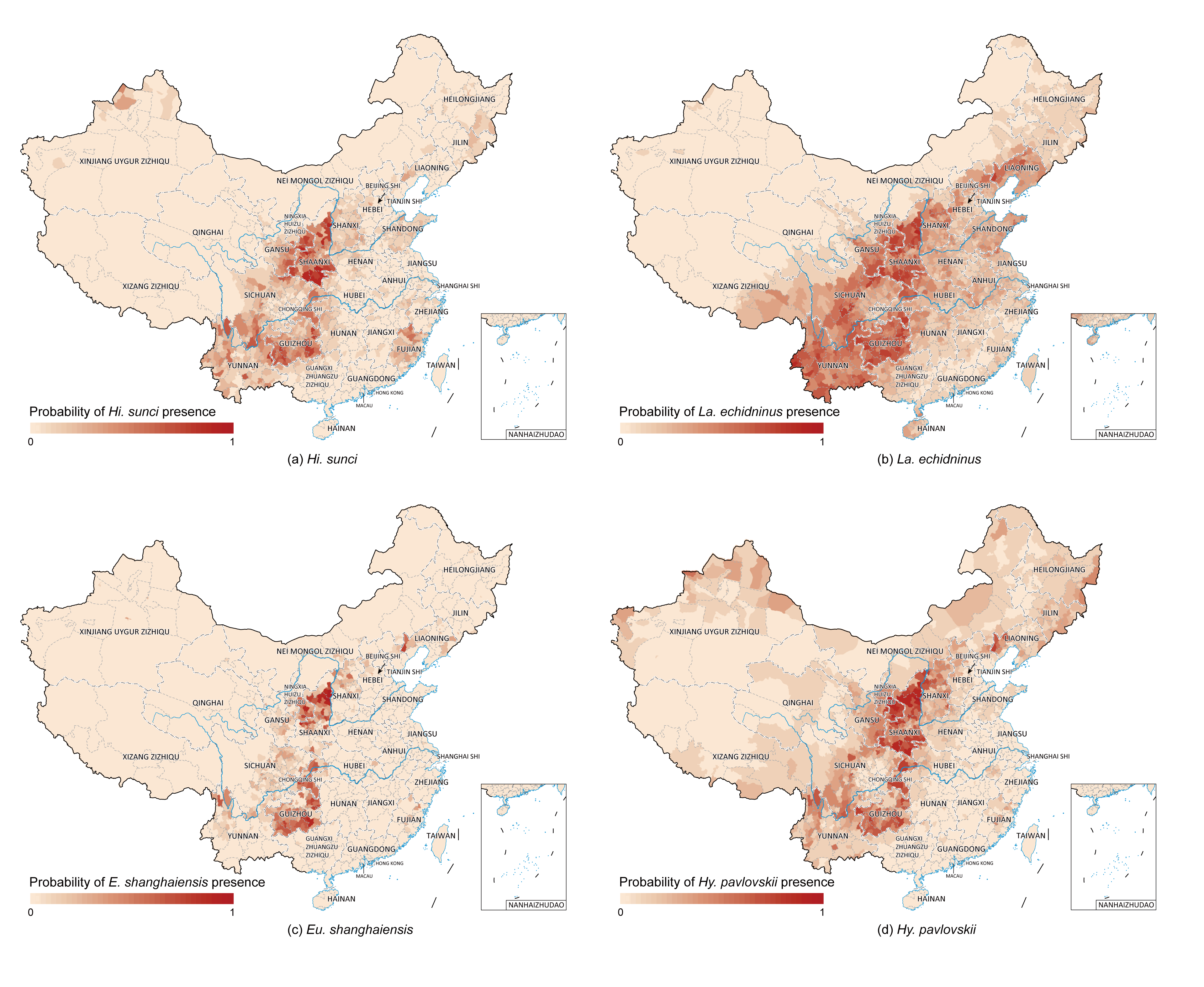
**

**Figure S36**: **The mean curves (red) and 95% percentiles (gray) for the effects of major predictors (RC≥5%) on the logit-transformed probability of occurrence of *O. tsutsugamushi* based on the ensemble of BRT models. Frequency distributions of the predictor is shown by the histograms in dark gray.
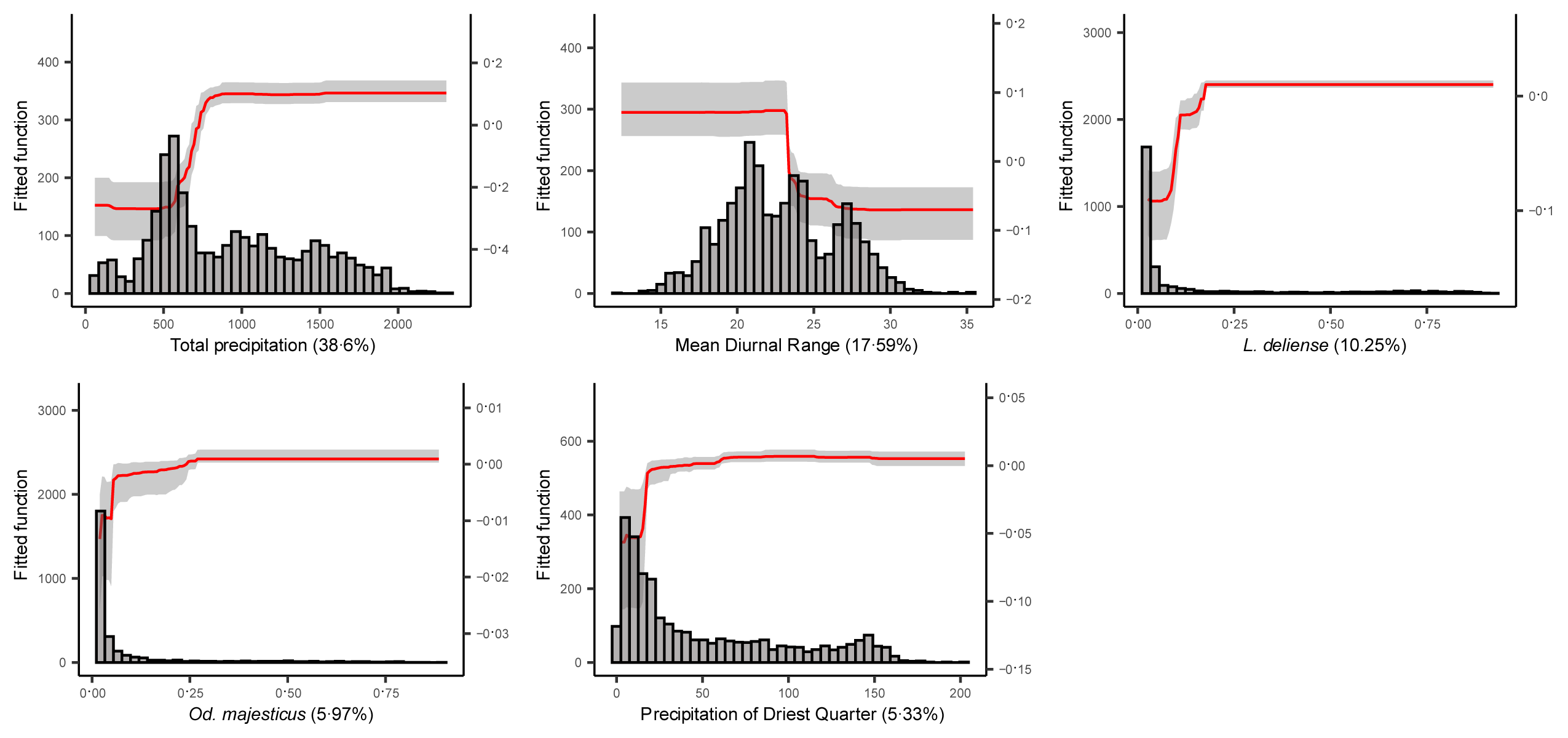
**

**Supplementary Tables**

**Table S1**: **The specific number of recorded counties with occurrence and references for each of 549 mite species from 100 genera in the mainland of China from 1978 to 2020.**

| **Genus (Number of species)** | **Species** | **Number of counties** | **Reference** |
| --- | --- | --- | --- |
| *Acomatacarus* (1) | *Ac. siseca* | 1 | [190] |
| *Alliphis* (1) | *Al. sinicus* | 2 | [105, 108] |
| *Allodermanysus* (1) | *Al. sanguineus* | 7 | [33, 61, 74, 98, 105, 191] |
| *Amblygamasus* (2) | *Am. atushiensissp* | 7 | [120, 137, 150] |
|  | *Am. liupanshanensis* | 1 | [120] |
| *Amblyseius* (3) | *Am. brientalis* | 1 | [151] |
|  | *Am. herbicolus* | 6 | [95, 108, 155] |
|  | *Am. tsugawai* | 2 | [95, 150] |
| *Ameroseius* (5) | *Am. curvatus* | 19 | [48, 105, 121, 130, 150] |
|  | *Am. guyimingi* | 3 | [121, 142] |
|  | *Am. magnisetosa* | 4 | [118, 121] |
|  | *Am. multus* | 4 | [48, 105, 121] |
|  | *Am. pavidus* | 1 | [121] |
| *Androlaelaps* (6) | *An. euryplatamus* | 2 | [122, 56] |
|  | *An. hsui* | 5 | [172, 191] |
|  | *An. karawaiewi* | 3 | [150, 190] |
|  | *An. novemspinosus* | 1 | [122] |
|  | *An. singularis* | 42 | [42, 50, 75, 77, 85, 105, 113, 118, 134, 140, 159, 190] |
|  | *An. trifurcatus* | 7 | [122] |
| *Anhemialges* (4) | *An. aegithalos* | 1 | [177] |
|  | *An. lioparus* | 1 | [177] |
|  | *An. seicercus* | 1 | [177] |
|  | *An. zosterops* | 1 | [177] |
| *Asca* (1) | *As. sinica* | 1 | [105] |
| *Ascoschoengastia* (6) | *As. indica* | 37 | [43, 127, 152, 190] |
|  | *As. latyshevi* | 6 | [190] |
|  | *As. leechi* | 11 | [15, 190] |
|  | *As. petauristae* | 1 | [144] |
|  | *As. sifanga* | 3 | [159] |
|  | *As. yunnanensis* | 3 | [35, 144] |
| *Austrophthiracarus* (2) | *Au. bacilliformis* | 1 | [187] |
|  | *Au. paralongisetosus* | 1 | [187] |
| *Blankaartia* (1) | *Bl. acuscutellaris* | 2 | [190] |
| *Chatia* (3) | *Cha. hertigi* | 3 | [37, 83] |
|  | *Cha. huanglungensis* | 4 | [159, 190] |
|  | *Cha. wissemani* | 3 | [159] |
| *Cheiroseius* (1) | *Che. fenghuangensis* | 2 | [114, 142] |
| *Cheladonta* (1) | *Che. ikaoensis* | 22 | [10, 13, 37, 77, 83, 84, 86, 190] |
| *Chiroptella* (2) | *Chi. anhui* | 1 | [13] |
|  | *Chi. pipistrella* | 1 | [190] |
| *Coleolaelaps* (2) | *Col. agrestis* | 3 | [122, 190] |
|  | *Col. tillaeCostaet* | 3 | [105, 122, 136] |
| *Cosmolaelaps* (6) | *Cos. acutiscutus* | 1 | [60] |
|  | *Cos. lapsretirugisp* | 1 | [123] |
|  | *Cos. miles* | 59 | [39, 45, 50, 61, 95, 98, 123, 190] |
|  | *Cos. ningxiaensis* | 5 | [118, 128, 159] |
|  | *Cos. paracuneifer* | 1 | [60] |
|  | *Cos. wangae* | 1 | [123] |
| *Dermanyssidae* (1) | *De. gallina* | 28 | [24, 27, 77, 88, 105, 118, 56, 166, 191] |
| *Dipolaelaps* (4) | *Di. jiangkouensis* | 6 | [73, 113] |
|  | *Di. longisetosus* | 1 | [123] |
|  | *Di. tongxinensis* | 2 | [105] |
|  | *Di. ubsunaris* | 8 | [105, 112, 123] |
| *Doloisia* (4) | *Do. brachypus* | 10 | [190] |
|  | *Do. chinensis* | 3 | [35, 190] |
|  | *Do. guangdongensis* | 10 | [65, 190] |
|  | *Do. hopuensis* | 4 | [65, 190] |
| *Echinolaelaps* (3) | *Ec. quensisi* | 1 | [190] |
|  | *Ec. traubi domrou* | 4 | [172, 191] |
|  | *Echinolaelaps* | 3 | [190] |
| *Epidamaeus* (5) | *Ep. alticola* | 1 | [125] |
|  | *Ep. cincinnatus* | 1 | [125] |
|  | *Ep. elegantis* | 1 | [125] |
|  | *Ep. longispinosus* | 1 | [125] |
|  | *Ep. yunnanensis* | 1 | [125] |
| *Eugamasus* (1) | *Eu. minus* | 1 | [120] |
| *Eulaelaps* (16) | *Eu. cricetuli* | 38 | [23, 27, 33, 53, 58, 136, 190] |
|  | *Eu. dongfangis* | 20 | [27, 50, 77, 81, 100, 191] |
|  | *Eu. dremomydis* | 8 | [73, 113] |
|  | *Eu. heptacanthus* | 6 | [105, 118] |
|  | *Eu. huzhuensis* | 8 | [61, 98, 113, 159] |
|  | *Eu. kolpakovae* | 10 | [190] |
|  | *Eu. laeuis* | 1 | [172] |
|  | *Eu. novus* | 4 | [36, 39, 190] |
|  | *Eu. pratentis* | 2 | [98, 190] |
|  | *Eu. shanghaiensis* | 59 | [39, 42, 85, 95, 101, 113, 122, 129, 151, 172, 191] |
|  | *Eu. silvestris* | 1 | [190] |
|  | *Eu. stabularis* | 188 | [11, 23, 24, 27, 39, 42, 53, 56, 58, 66, 73, 75, 77, 81, 82, 85, 87, 88, 100, 104, 113, 122, 136, 165, 174, 186, 191] |
|  | *Eu. subshanghaiensis* | 1 | [190] |
|  | *Eu. substabularis* | 20 | [87, 105, 113, 122, 128, 136, 140, 151, 164] |
|  | *Eu. tsinghaiensis* | 3 | [33, 122] |
|  | *Eu. widesternalis* | 14 | [36, 105, 118, 122] |
| *Euryparasitus* (2) | *Eur. citelli* | 1 | [105] |
|  | *Eur. emarginatus* | 29 | [11, 50, 74, 105, 173, 190] |
| *Euschoengastia* (10) | *Eu. alpina* | 14 | [13, 37, 77, 83, 190] |
|  | *Eu. koreaensis* | 2 | [190] |
|  | *Eu. olsufjevi* | 3 | [191] |
|  | *Eu. tanggulensis* | 1 | [145] |
|  | *Eus. audyi* | 3 | [190] |
|  | *Eus. audyi var* | 1 | [190] |
|  | *Eus. ikaoensis* | 2 | [190] |
|  | *Eus. indica* | 27 | [190] |
|  | *Eus. lorius* | 4 | [190] |
|  | *Eus. schlugeri var. fukienensis* | 1 | [190] |
| *Eutrombicula* (1) | *Eut. hirsti* | 1 | [144] |
| *Eviphis* (1) | *Ev. cryptognathus* | 1 | [105] |
| *Fissicepheus* (2) | *Fi. aokii* | 2 | [180] |
|  | *Fi. wangae* | 1 | [180] |
| *Gahrliepia* (31) | *G. agrariusia* | 24 | [43, 159, 190] |
|  | *G. chekiangensis* | 9 | [75, 159, 190] |
|  | *G. chinensis* | 45 | [16, 191] |
|  | *G. Cocteau* | 3 | [190] |
|  | *G. deqinensis* | 3 | [159] |
|  | *G. Ivin* | 9 | [190] |
|  | *G. jiangxi* | 2 | [75, 97] |
|  | *G. linguipelta* | 3 | [159] |
|  | *G. longipedalis* | 32 | [159, 163, 165] |
|  | *G. lui* | 1 | [190] |
|  | *G. miyi* | 3 | [159] |
|  | *G. myriosetosa* | 1 | [190] |
|  | *G. neosinensis* | 3 | [191] |
|  | *G. octosetosa* | 48 | [43, 75, 77, 84, 86, 97, 191] |
|  | *G. pacifica* | 34 | [66, 191] |
|  | *G. parapacifica* | 42 | [191] |
|  | *G. pingtan* | 3 | [75, 97, 190] |
|  | *G. puning* | 11 | [190] |
|  | *G. quemao* | 2 | [190] |
|  | *G. radiopunctata* | 5 | [190] |
|  | *G. romeri* | 2 | [190] |
|  | *G. sichuansis* | 2 | [190] |
|  | *G. silvatica* | 3 | [159] |
|  | *G. tibet* | 1 | [190] |
|  | *G. wuchihensis* | 12 | [190] |
|  | *G. yangchenensis* | 32 | [65, 75, 170, 190] |
|  | *G. youyan* | 1 | [190] |
|  | *G. yunnanensis* | 7 | [140, 159, 190] |
|  | *Ga. agrariusia* | 4 | [13] |
|  | *Ga. anhuiensis* | 1 | [13] |
|  | *Ga. kiangsiensis* | 1 | [13] |
| *Gahrliepia(Walchia)* (1) | *Gahrliepia(Walchia) fragilis* | 2 | [191] |
| *Galumnella* (3) | *Gal. nonporosa* | 2 | [184] |
|  | *Gal. parageographica* | 3 | [184] |
|  | *Gal. sidorchukae* | 1 | [184] |
| *Gamasholaspis* (2) | *Gam. duyunens* | 3 | [159] |
|  | *Gam. sinicus* | 4 | [75, 85, 190] |
| *Gamasodes* (3) | *Gam. guoluoensis* | 1 | [120] |
|  | *Gam. micherdzinskii* | 2 | [120] |
|  | *Gam. tongdensis* | 1 | [120] |
| *Haemogamasus* (49) | *Ha. ambulans* | 34 | [24, 36, 53, 58, 122, 191] |
|  | *Ha. angustus* | 1 | [122] |
|  | *Ha. bifurcatus* | 1 | [122] |
|  | *Ha. calandrellrs* | 6 | [81, 122, 128, 190] |
|  | *Ha. citelli* | 14 | [36, 118, 122, 190] |
|  | *Ha. clethrionomidis* | 2 | [190] |
|  | *Ha. Concavus* | 3 | [33, 122, 191] |
|  | *Ha. dauricus* | 46 | [33, 73, 90, 113, 122, 151, 155, 190, 191] |
|  | *Ha. dorsalis* | 6 | [113, 159, 190] |
|  | *Ha. emeiensis* | 3 | [159] |
|  | *Ha. huangzhongensis* | 3 | [122] |
|  | *Ha. ivanovi* | 8 | [33, 53, 122, 190] |
|  | *Ha. kitanoi* | 46 | [23, 33, 36, 53, 98, 112, 122, 156, 191] |
|  | *Ha. kusumotoi* | 21 | [122, 190] |
|  | *Ha. liponyssoides* | 43 | [27, 53, 58, 122, 159, 186, 190] |
|  | *Ha. macrodentilis* | 5 | [22, 33, 73, 122] |
|  | *Ha. mandschuricus* | 83 | [23, 24, 33, 36, 53, 58, 74, 98, 112, 118, 122, 128, 136, 156, 191] |
|  | *Ha. monticola* | 35 | [39, 50, 73, 75, 85, 104, 113, 129, 134, 159, 172, 190] |
|  | *Ha. nidi* | 3 | [159] |
|  | *Ha. nidiformis* | 24 | [24, 73, 98, 113, 122, 159, 191] |
|  | *Ha. oliviformis* | 37 | [67, 73, 113, 122, 140, 159, 165, 190] |
|  | *Ha. paradauricus* | 4 | [122] |
|  | *Ha. parascaptoris* | 1 | [191] |
|  | *Ha. pingi* | 27 | [85, 122, 136] |
|  | *Ha. pontiger* | 6 | [44, 113, 165] |
|  | *Ha. qinghaiensis* | 3 | [122] |
|  | *Ha. quadratus* | 4 | [113, 159] |
|  | *Ha. quadrisetatus* | 43 | [73, 75, 113, 122, 190] |
|  | *Ha. serdjukovae* | 49 | [53, 58, 122, 159, 190] |
|  | *Ha. szechwanensis* | 5 | [73, 122] |
|  | *Ha. trapezoideus* | 1 | [190] |
|  | *Ha. trifiuecisetus* | 3 | [159] |
|  | *Ha. yunlongensis* | 4 | [122, 159] |
|  | *Ha. zachvatkini altaicus* | 9 | [190] |
|  | *Ha.submandschuricus* | 2 | [122] |
| *Haemolaelaps* (15) | *Ha. casalis* | 91 | [36, 39, 41, 50, 60, 61, 77, 80, 88, 98, 99, 104, 105, 111-113, 115, 123, 128, 129, 134, 136, 142, 174, 190] |
|  | *Ha. chinensis* | 12 | [41, 50, 104, 113, 134, 159, 190] |
|  | *Ha. cordatus* | 8 | [102, 113, 159, 190] |
|  | *Ha. fragilis* | 1 | [105] |
|  | *Ha. glasgowf* | 259 | [24, 36, 39, 44, 50, 53, 58, 61, 66, 73, 74, 75, 77, 82, 88, 97, 98, 99, 100, 101, 105, 106, 109, 111, 113, 117, 118, 123, 129, 134, 159, 174, 186, 191] |
|  | *Ha. laensis* | 2 | [190] |
|  | *Ha. latiporus* | 1 | [105] |
|  | *Ha. liae* | 1 | [190] |
|  | *Ha. longirodus* | 1 | [123] |
|  | *Ha. sclerotarsus* | 4 | [105, 123] |
|  | *Ha. semidesertus* | 45 | [24, 36, 98, 112, 113, 123, 191] |
|  | *Ha. traubi* | 5 | [50, 113, 190] |
|  | *Ha. triangular* | 58 | [66, 73, 105, 123, 129, 134, 136, 172, 190] |
|  | *Ha. yiliensis* | 2 | [98, 123] |
|  | *Ha. zhongweiensis* | 1 | [105] |
| *Helenicula* (14) | *He. abaensis* | 3 | [159] |
|  | *He. aosuiensis* | 1 | [191] |
|  | *He. aulacochaeta* | 3 | [159] |
|  | *He. globularis* | 5 | [35, 190] |
|  | *He. hongkongenisis* | 1 | [35] |
|  | *He. hsui* | 4 | [65] |
|  | *He. kohlsi* | 6 | [190] |
|  | *He. kouensis* | 5 | [190] |
|  | *He. litchia* | 3 | [190] |
|  | *He. olsufjevi* | 3 | [159] |
|  | *He. rattihaikonga* | 6 | [190] |
|  | *He. saihsuensis* | 2 | [75, 190] |
|  | *He. simena* | 27 | [35, 65, 139, 140, 152, 159, 191] |
|  | *He. yunnanensis* | 4 | [159, 190] |
| *Herpetacarus* (34) | *Her. fukienensis* | 4 | [190] |
|  | *Her. cheni* | 1 | [190] |
| *Hirstionyssus* (33) | *Hi. ansaiensis* | 1 | [120] |
|  | *Hi. citelli* | 9 | [105, 120, 136] |
|  | *Hi. confucianus* | 10 | [58, 105, 190] |
|  | *Hi. criceti* | 69 | [58, 66, 77, 98, 105, 112, 120, 128, 191] |
|  | *Hi. formis* | 1 | [120] |
|  | *Hi. gansuensis* | 5 | [22, 120, 159] |
|  | *Hi. georgicus* | 6 | [105, 120] |
|  | *Hi. huangheensis* | 13 | [22, 120, 128] |
|  | *Hi. isabellinus* | 43 | [24, 53, 58, 66, 85, 98, 113, 120, 136, 151, 186, 191] |
|  | *Hi. kirinensis* | 1 | [190] |
|  | *Hi. merldianus* | 2 | [120, 137, 150] |
|  | *Hi. montanus* | 4 | [120] |
|  | *Hi. musculi* | 75 | [36, 53, 58, 77, 82, 98, 105, 120] |
|  | *Hi. myospalacis* | 4 | [190] |
|  | *Hi. neosinicus* | 6 | [73, 113, 120, 136, 190] |
|  | *Hi. ningxiaensis* | 2 | [105, 120] |
|  | *Hi. ochotonae* | 22 | [22, 105, 120, 190] |
|  | *Hi. phodopi* | 7 | [105, 120, 136] |
|  | *Hi. pratentis* | 1 | [120] |
|  | *Hi. punctatus* | 2 | [120] |
|  | *Hi. qinghaiensis* | 1 | [120] |
|  | *Hi. sciurinus* | 1 | [190] |
|  | *Hi. shensiensis* | 15 | [22, 73, 105, 120, 128, 190] |
|  | *Hi. soricis* | 2 | [120] |
|  | *Hi. sunci* | 117 | [11, 39, 42, 44, 50, 53, 61, 77, 82, 104, 106, 111, 113, 120, 129, 134, 140, 159, 174, 191] |
|  | *Hi. szechuanicus* | 1 | [190] |
|  | *Hi. Tamias sibiricus* | 1 | [190] |
|  | *Hi. transiliensis* | 5 | [190] |
|  | *Hi. transiliensis neimongkuensis* | 15 | [53, 61, 105, 112, 118, 120] |
|  | *Hi. trogopteri* | 1 | [190] |
|  | *Hi. xinhaiensis* | 1 | [120] |
|  | *Hi. xinjiangensis* | 10 | [120] |
|  | *Hi. zaisanica* | 1 | [120] |
|  | *Hi. ventricosus* | 10 | [53, 77, 190] |
| *Hsuella* (1) | *Hs. hubeiensis* | 1 | [75] |
| *Hyperlaelaps* (2) | *Hy. microti* | 30 | [24, 36, 50, 53, 58, 62, 113, 123, 190] |
|  | *Hy. orientalis* | 5 | [105, 123, 134, 136] |
| *Hypoaspis* (32) | *Hy. aculeifer* | 2 | [122, 190] |
|  | *Hy. Canestrini* | 1 | [105] |
|  | *Hy. chelaris* | 3 | [159] |
|  | *Hy. chianensis* | 9 | [113, 140, 159, 164] |
|  | *Hy. chini* | 2 | [105] |
|  | *Hy. debilis* | 5 | [60, 115, 122, 134] |
|  | *Hy. digitalis* | 3 | [122] |
|  | *Hy. equitas* | 2 | [105, 122] |
|  | *Hy. haiyvanensis* | 3 | [105, 122, 136] |
|  | *Hy. hrdyi* | 1 | [60] |
|  | *Hy. kargi* | 4 | [53, 63, 142, 160] |
|  | *Hy. kirinensis* | 4 | [73, 79, 115, 190] |
|  | *Hy. laeuis* | 3 | [105] |
|  | *Hy. leeae* | 2 | [105, 113] |
|  | *Hy. linteyini* | 2 | [122, 181] |
|  | *Hy. lubrica* | 117 | [11, 24, 27, 39, 44, 50, 53, 58, 60, 61, 75, 85, 97, 98, 105, 109, 112, 113, 118, 122, 129, 134, 136, 140, 141, 173, 178, 191] |
|  | *Hy. miles* | 36 | [85, 105, 113, 129, 165, 191] |
|  | *Hy. ningxiaensis* | 1 | [105] |
|  | *Hy. paracuneifer* | 6 | [105, 118, 128, 135, 136] |
|  | *Hy. pavlovskii* | 147 | [23, 27, 39, 42, 44, 50, 53, 61, 73, 74, 75, 77, 81, 82, 85, 97, 98, 104, 105, 109, 113, 123, 128, 129, 134, 140, 141, 150, 156, 159, 165, 172, 186, 191] |
|  | *Hy. praesternalis* | 1 | [60] |
|  | *Hy. qinghaiensis* | 1 | [122] |
|  | *Hy. siensis* | 1 | [190] |
|  | *Hy. sinensis* | 1 | [105] |
|  | *Hy. spinaperaf finis* | 3 | [159] |
|  | *Hy. submantana* | 1 | [105] |
|  | *Hy. subminor* | 1 | [105] |
|  | *Hy. subpietus* | 1 | [105] |
|  | *Hy. sungaris* | 1 | [60] |
|  | *Hy. tengi* | 3 | [105] |
|  | *Hy. vacua* | 1 | [118] |
|  | *Hy. wangae* | 1 | [105] |
| *Intermedialia* (2) | *I. hegu* | 1 | [35, 144] |
|  | *I. yunensis* | 1 | [190] |
| *Kleemannia* (2) | *K. plumigera* | 1 | [190] |
|  | *K. plumosus* | 1 | [190] |
| *Laelaps* (19) | *La. agilis* | 53 | [24, 73, 98, 123] |
|  | *La. algericus Hirst* | 80 | [44, 61, 74, 98, 105, 111, 112, 113, 123, 134, 136, 191] |
|  | *La. cheni* | 3 | [159] |
|  | *La. chini* | 30 | [62, 73, 75, 97, 113, 123, 134, 140, 159, 174, 191] |
|  | *La. clethrionomydis* | 59 | [53, 113, 123, 129, 190] |
|  | *La. cochlearis* | 6 | [76] |
|  | *La. echidninus* | 200 | [27, 41, 42, 46, 50, 51, 53, 58, 62, 64, 72, 73, 74, 75, 77, 81, 88, 92, 99, 100, 104, 105, 106, 110, 111, 123, 131, 140, 141, 167, 172, 174, 191] |
|  | *La. extremi* | 13 | [73, 98, 112, 113, 123] |
|  | *La. fukienensis* | 30 | [19, 42, 73, 75, 76, 97, 104, 113, 191] |
|  | *La. hongaiensis* | 4 | [113, 159] |
|  | *La. jettmari* | 188 | [32, 33, 42, 44, 50, 53, 58, 66, 75, 85, 99, 101, 105, 106, 109, 113, 123, 129, 131, 134, 172, 174, 186, 191] |
|  | *La. laeuis* | 4 | [118, 190] |
|  | *La. liui* | 12 | [113, 134, 159, 164, 190] |
|  | *La. micromydis* | 10 | [53, 81, 113, 159, 190] |
|  | *La. multispinosus* | 5 | [159, 190] |
|  | *La. nuttalli* | 140 | [41, 42, 46, 50, 51, 53, 58, 62, 64, 72, 73, 75, 82, 85, 88, 92, 100, 104, 106, 123, 129, 140, 142, 172, 174, 186, 191] |
|  | *La. taingueni* | 18 | [50, 102, 113, 123, 134, 174, 191] |
|  | *La. traubi* | 50 | [113, 123, 140] |
|  | *La. turkestanicus* | 83 | [50, 72, 73, 75, 104, 113, 123, 151] |
| *Laelaspis* (1) | *La. laevis* | 1 | [118, 190] |
| *Lasioseius* (7) | *La. ometes* | 2 | [105, 121] |
|  | *La. paraconfusus* | 1 | [121] |
|  | *La. punctatus* | 2 | [121] |
|  | *La. qianensis* | 3 | [159] |
|  | *La. qinghaiensis* | 4 | [121, 159] |
|  | *La. schizopilus* | 1 | [121] |
|  | *La..multispathus* | 1 | [121] |
| *Leptotrombidium* (73) | *L. (Trombiculindus)hylomydis* | 1 | [35] |
|  | *L. akamushi* | 14 | [159, 170, 190] |
|  | *L. allosetum* | 6 | [159, 170] |
|  | *L. alpinum* | 3 | [159] |
|  | *L. baoshui* | 3 | [159] |
|  | *L. biluoxueshanense* | 3 | [159] |
|  | *L. cangjiangense* | 4 | [35, 159] |
|  | *L. caudatum* | 3 | [159] |
|  | *L. cheni* | 2 | [190] |
|  | *L. deliense* | 126 | [17, 21, 25, 28, 29, 31, 35, 38, 40, 43, 54, 55, 57, 68, 75, 78, 80, 84, 91, 94, 99, 119, 149, 152, 159, 165, 188, 191] |
|  | *L. densipunctatum* | 5 | [132, 159] |
|  | *L. deplanoscutum* | 4 | [152, 159] |
|  | *L. dongluoense* | 3 | [84] |
|  | *L. eothenomydis* | 4 | [89, 159] |
|  | *L. fuji* | 44 | [43, 152, 158, 165, 191] |
|  | *L. fujianense* | 7 | [84, 159] |
|  | *L. gemiticulum* | 2 | [83] |
|  | *L. gongshanense* | 4 | [144, 159] |
|  | *L. hiemalis* | 3 | [159] |
|  | *L. huangdi* | 1 | [69] |
|  | *L. hylomydis* | 2 | [190] |
|  | *L. imphalum* | 4 | [144, 159] |
|  | *L. intermedium* | 56 | [13, 31, 37, 75, 86, 157] |
|  | *L. jiangsuense* | 2 | [190] |
|  | *L. jinmai* | 3 | [159] |
|  | *L. kaohuense* | 7 | [31, 84] |
|  | *L. kawamurai* | 2 | [190] |
|  | *L. kitasatoi* | 8 | [75, 159, 191] |
|  | *L. laxoscutum* | 4 | [159, 190] |
|  | *L. linhuakongense* | 13 | [43, 70, 86, 159, 162, 191] |
|  | *L. linji* | 3 | [159] |
|  | *L. longchuanense* | 3 | [159] |
|  | *L. longimedium* | 3 | [159] |
|  | *L. lushaneusis* | 3 | [159] |
|  | *L. miyajimai* | 1 | [190] |
|  | *L. muntiaci* | 3 | [159] |
|  | *L. myotis* | 3 | [159] |
|  | *L. nujiange* | 1 | [190] |
|  | *L. orientale* | 8 | [37, 81, 190] |
|  | *L. pallidum* | 21 | [31, 75, 84, 191] |
|  | *L. palpale* | 39 | [12, 37, 77, 81, 91, 106, 143, 191] |
|  | *L. pavlovskyi* | 2 | [190] |
|  | *L. qujingense* | 3 | [159] |
|  | *L. robustisetum* | 3 | [159] |
|  | *L. rubellum* | 37 | [47, 158, 171] |
|  | *L. rufocanum* | 4 | [34, 159] |
|  | *L. rupestr* | 3 | [75, 97] |
|  | *L. rusticum* | 9 | [152, 159, 190] |
|  | *L. scutellare* | 87 | [13, 20, 28, 31, 35, 43, 65, 77, 78, 84, 89, 91, 126, 140, 143, 152, 158, 159, 168, 169, 175, 176, 179, 183, 188] |
|  | *L. scutellare basoglabrose* | 10 | [191] |
|  | *L. sheshui* | 3 | [159] |
|  | *L. shumiense* | 1 | [52] |
|  | *L. shuyui* | 3 | [159] |
|  | *L. sinicum* | 1 | [89] |
|  | *L. spicanisdum* | 3 | [159] |
|  | *L. striatum* | 14 | [13, 190] |
|  | *L. subintermedium* | 1 | [34] |
|  | *L. submagenus* | 2 | [75] |
|  | *L. subpalpale* | 10 | [59] |
|  | *L. sucnsc* | 3 | [159] |
|  | *L. taishanicum* | 10 | [77, 86, 159] |
|  | *L. tibet* | 1 | [190] |
|  | *L. tsinghaiense* | 1 | [190] |
|  | *L. vesperlilum* | 1 | [52] |
|  | *L. wangi* | 3 | [159] |
|  | *L. xiaguanense* | 5 | [35] |
|  | *L. yantangshanense* | 1 | [190] |
|  | *L. yongshengense* | 3 | [159] |
|  | *L. youyi* | 1 | [52] |
|  | *L. yui* | 53 | [13, 18, 31, 84, 94, 138, 146, 152, 158, 190] |
|  | *L. yulini* | 3 | [159] |
|  | *L. zeta* | 8 | [37, 159] |
|  | *L. zhangmuense* | 1 | [52] |
| *Macrocheles* (12) | *M. decoloratus* | 71 | [22, 23, 24, 27, 33, 53, 61, 75, 85, 97, 118, 121, 128, 156, 178] |
|  | *M. glaber* | 47 | [53, 73, 75, 77, 95, 102, 105, 128, 142, 150, 174, 190] |
|  | *M. kolpakovae* | 2 | [190] |
|  | *M. matrius* | 44 | [23, 36, 75, 85, 98, 105, 112, 116, 121, 135, 161, 190] |
|  | *M. merdarius* | 17 | [73, 79, 105, 121, 135, 174, 190] |
|  | *M. muscaedomesticae* | 16 | [75, 85, 95, 104, 106, 122, 148, 190] |
|  | *M. penicilliger* | 1 | [190] |
|  | *M. plumiventris* | 7 | [190] |
|  | *M. sinicus* | 2 | [121] |
|  | *M. subbadius* | 1 | [190] |
|  | *M. trausbaicalicus* | 4 | [190] |
|  | *M. vernalis* | 4 | [105, 121] |
| *Microtrombicula* (4) | *M. munda* | 11 | [190] |
|  | *M. ndchatrami* | 1 | [35] |
|  | *M. vitosa* | 4 | [190] |
|  | *M. yanmai* | 1 | [190] |
| *Miyatrombicula* (1) | *Mi. esoensis* | 2 | [190] |
| *Multisetosa* (3) | *Mu. compta* | 2 | [190] |
|  | *Mu. Inner Mongolia* | 1 | [190] |
|  | *Mu. tibet* | 2 | [190] |
| *Myonyssus* (3) | *My. duensis* | 3 | [190] |
|  | *My. ochotonae* | 1 | [190] |
|  | *My. shibatai* | 1 | [120] |
| *Mysolaelaps* (1) | *My. cunicularis* | 3 | [159] |
| *Neogamasus* (1) | *N. mengi* | 1 | [60] |
| *Neoschoengasta* (7) | *N. americana var hexasternosetosa* | 2 | [190] |
|  | *N. americana var solomonis* | 4 | [190, 191] |
|  | *N. gallinarum* | 38 | [27, 190] |
|  | *N. monticola* | 2 | [190] |
|  | *N. pomeranzevi* | 29 | [83, 190, 191] |
|  | *N. shihwanensis* | 8 | [190] |
|  | *N. taoli* | 1 | [190] |
| *Neotrombicula* (10) | *N. anax* | 3 | [98, 112, 190] |
|  | *N. banchang* | 2 | [190] |
|  | *N. gardellai* | 1 | [190] |
|  | *N. hsui* | 3 | [84] |
|  | *N. japonica* | 15 | [37, 66, 83, 190] |
|  | *N. microti* | 1 | [190] |
|  | *N. sinica* | 2 | [145, 190] |
|  | *N. talmiensis* | 1 | [83] |
|  | *N. tamiyai* | 3 | [34, 37, 83] |
|  | *N. wendai* | 4 | [75, 145, 147, 190] |
| *Neparholaspis* (1) | *N. subarcuatus* | 3 | [159] |
| *Notoedres* (1) | *N. cativar* | 2 | [30] |
| *Odontacarus* (2) | *Od. majesticus* | 75 | [43, 84, 86, 91, 191] |
|  | *Od. niaoer* | 1 | [190] |
| *Ololaelaps* (2) | *Ol. sinensisi* | 1 | [105] |
|  | *Ol. ussuriensis* | 8 | [142] |
| *Oribotritia* (5) | *Or. angusta Mahunka* | 1 | [154] |
|  | *Or. asiatica Hammer* | 1 | [154] |
|  | *Or. chichijimensis Aoki* | 1 | [154] |
|  | *Or. gigas Bayoumi & Mahunka* | 1 | [154] |
|  | *Or. hunchunensis* | 1 | [154] |
| *Ornithonyssus* (4) | *Or. bacoti* | 113 | [11, 39, 42, 44, 46, 50, 75, 77, 85, 88, 95, 96, 97, 98, 100, 104, 105, 107, 113, 129, 140, 165, 172, 174, 191] |
|  | *Or. bursa* | 7 | [95, 190] |
|  | *Or. dui* | 1 | [191] |
|  | *Or. sylviarum* | 6 | [75, 85, 97, 191] |
| *Oryctolaelaps* (1) | *Or. bibikovae* | 10 | [105, 123, 190] |
| *Pachylaelaps* (1) | *Pac. siculus* | 6 | [105, 115, 135, 142] |
| *Parasitellus* (2) | *Pa. crinitus* | 1 | [120] |
|  | *Pa. fucorum* | 2 | [120] |
| *Parasitus* (12) | *Pa. beta* | 5 | [105, 128, 135, 155] |
|  | *Pa. bispinatus* | 1 | [60] |
|  | *Pa. coleoptratorus* | 9 | [60, 79, 105, 118, 135, 142] |
|  | *Pa. consanguineus* | 12 | [22, 33, 95, 102, 120, 130, 142, 173, 174] |
|  | *Pa. diviortus* | 5 | [115, 120] |
|  | *Pa. fimetorum* | 18 | [79, 95, 120, 128, 135, 155, 174] |
|  | *Pa. mustearum* | 3 | [105, 120, 142] |
|  | *Pa. taparum* | 2 | [120] |
|  | *Pa. tengkuofani* | 1 | [60] |
|  | *Pa. tichonirovi* | 5 | [105, 128, 135] |
|  | *Pa. wangdunqingi* | 1 | [60] |
|  | *Pa. wentinghuani* | 1 | [60] |
| *Parholaspulus* (2) | *Pa. alstoni* | 2 | [95, 190] |
|  | *Pa. lusventricosus* | 1 | [190] |
| *Pellonyssus* (1) | *Pe. stenosternus* | 2 | [190] |
| *Pergalumna* (3) | *Pe. amorpha* | 1 | [182] |
|  | *Pe. jongkyui* | 1 | [182] |
|  | *Pe. sidorchukae* | 1 | [182] |
| *Pleuronectocelaeno* (1) | *Pl. barbara Athias-Henriot* | 1 | [185] |
| *Podocinum* (2) | *Po. anhuiensis* | 1 | [190] |
|  | *Po. aokiiIsikawa* | 1 | [114] |
| *Poecilochirus* (2) | *Po. necrophori* | 32 | [36, 95, 111, 120, 191] |
|  | *Po. subteraneus* | 11 | [105, 120, 190] |
| *Porrhostaspis* (1) | *Po. setosasp* | 1 | [120] |
| *Proctolaelaps* (4) | *Pr. fiseri* | 5 | [49, 102, 115, 135] |
|  | *Pr. liupanshanensis* | 1 | [120] |
|  | *Pr. pistilli* | 3 | [159] |
|  | *Pr. pygmaeus* | 62 | [39, 44, 60, 79, 82, 95, 105, 115, 121, 129, 135, 140, 155, 174] |
| *Qinghailaelaps* (1) | *Q. gui* | 1 | [105] |
| *Rhyzolaelaps* (1) | *Rh. rhizmydis* | 1 | [54] |
| *Riedlinia* (1) | *Ri. sinicum* | 1 | [190] |
| *Rudnicula* (2) | *Ru. tianmu* | 1 | [190] |
|  | *Ru. tsochiensis* | 1 | [190] |
| *Schizocyrtillus* (1) | *S. fuzhouensis* | 1 | [185] |
| *Schoengastia* (12) | *S. loudangicola* | 4 | [190] |
|  | *S. obtusispura* | 1 | [190] |
|  | *S. pseudoschuffmeri* | 1 | [190] |
|  | *S. yunnanensis* | 3 | [190] |
| *Schoengastiella* (8) | *S. confuciana* | 21 | [43, 75, 77, 84, 86, 97, 191] |
|  | *S. himalayana* | 1 | [190] |
|  | *S. luensis* | 1 | [190] |
|  | *S. Niviventer* | 3 | [190] |
|  | *S. punctata* | 2 | [190] |
|  | *S. qomolangma* | 1 | [190] |
|  | *S. saduski* | 16 | [13, 190] |
|  | *S. xizangensis* | 1 | [190,] |
| *Sinoseius* (1) | *Si. lobatus* | 2 | [105, 121] |
| *Siseca* (1) | *Sis. xixie* | 1 | [190] |
| *Spinturnix* (3) | *Sp. pipistrella* | 1 | [190] |
|  | *Sp. plecotinus* | 1 | [105] |
|  | *Sp. setosus* | 1 | [190] |
| *Steatonyssus* (2) | *St. bat* | 1 | [190] |
|  | *St. longispinosus* | 4 | [75, 105, 134, 190] |
| *Tragardhula* (2) | *Tra. accuscutellaris* | 6 | [190] |
|  | *Tra. koomori* | 1 | [190,] |
| *Tricholaelaps* (1) | *Tr. myonysognathus* | 99 | [39, 42, 44, 46, 50, 73, 75, 82, 85, 88, 101, 104, 113, 123, 129, 134, 141, 147, 172, 174, 191] |
| *Trombicula* (4) | *T. henesis* | 17 | [190] |
|  | *T. qujiang* | 1 | [190] |
|  | *T. tungshihensis* | 2 | [190] |
|  | *T. wichmanni* | 24 | [190] |
| *Trombicula(Eutrombicula)* (1) | *Trombicula(Eutrombicula). hirsti* | 4 | [191] |
| *Trombiculindus* (9) | *T. acanthosphenus* | 1 | [75] |
|  | *T. bambusoides* | 43 | [75, 84, 97, 158, 159, 190] |
|  | *T. chilie* | 3 | [159] |
|  | *T. cuneatus* | 8 | [190] |
|  | *T. duoji* | 1 | [190] |
|  | *T. kuanye* | 3 | [159] |
|  | *T. qianye* | 3 | [159] |
|  | *T. sanxiaensis* | 1 | [75] |
|  | *T. yunnanus* | 5 | [93, 159, 190] |
| *Typhlodromus* (1) | *Typ. macrum* | 1 | [115] |
| *Tyrophagrs* (1) | *Tyr. mimlongior* | 1 | [190] |
| *Varroa* (2) | *Va. Clareae* | 2 | [105, 190,] |
|  | *Va. destructor* | 2 | [85, 105] |
| *Veigaia* (1) | *Ve. kochi* | 1 | [33] |
| *Vulgarogamasus* (14) | *V. burchanensis* | 5 | [95, 124] |
|  | *V. haiyuanensis* | 3 | [105, 118, 124] |
|  | *V. ningxiaensis* | 2 | [124] |
|  | *V. oligochaetus* | 1 | [124] |
|  | *V. oudemansi* | 7 | [105, 124, 173] |
|  | *V. palmatussp* | 1 | [124] |
|  | *V. plumosus* | 3 | [159, 163, 165] |
|  | *V. plunosus* | 1 | [124] |
|  | *V. qiangorlosana* | 3 | [159] |
|  | *V. qinghaiensis* | 1 | [124] |
|  | *V. radialis* | 1 | [124] |
|  | *V. sinicus* | 1 | [124] |
|  | *V. squarrosus* | 2 | [124] |
|  | *V. trifidus* | 5 | [22, 74, 124, 155] |
| *Walchia* (19) | *W. acustascuta* | 3 | [13] |
|  | *W. chinensis* | 80 | [13, 14, 26, 35, 43, 65, 71, 72, 75, 84, 103, 144, 170, 190] |
|  | *W. chuanica* | 6 | [159] |
|  | *W. enode* | 3 | [159] |
|  | *W. ewingi* | 15 | [35, 159, 190] |
|  | *W. fragilis* | 41 | [37, 69, 83, 86, 191] |
|  | *W. fulleri* | 1 | [190] |
|  | *W. isonyehia* | 1 | [190] |
|  | *W. koi* | 5 | [132] |
|  | *W. luoban* | 1 | [190] |
|  | *W. micropelta* | 28 | [35, 133, 144, 146, 189, 190] |
|  | *W. octosetosa* | 1 | [190] |
|  | *W. oligosetosa* | 17 | [75, 84, 190] |
|  | *W. pacifica* | 51 | [13, 43, 72, 75, 77, 84, 86, 97, 102, 190] |
|  | *W. parapacifica* | 49 | [43, 65, 72, 84, 191] |
|  | *W. rustica* | 3 | [190] |
|  | *W. Tibet* | 1 | [190] |
|  | *W. xishaensis* | 3 | [159] |
|  | *W. zangnanica* | 3 | [159] |
| *Walchiella* (1) | *Wa. Tibet* | 1 | [190] |
| *Whartonia* (2) | *Wh. recurvata* | 1 | [190] |
|  | *Wh. mapaensis* | 1 | [190] |

**Table S2**: **The specific references for** **pathogens detected in blood-sucking mites in China from 1978 to 2020.**

| **Pathogen** | **Reference** |
| --- | --- |
| *Orientia tsutsugamushi* | [194‒196, 198, 199, 201, 202, 204, 206‒213, 215, 216, 218‒228, 231, 232, 234‒244, 247-253, 255, 257‒261, 263‒266, 268‒270, 272‒273, 282‒304, 306‒313, 315, 317‒330, 332‒346] |
| *Coxiella burnetii* | [267] |
| *Rickettsia felis* | [314, 331] |
| *Rickettsia australis* | [331] |
| Unnamed *Rickettsia* sp. TwKM02 | [331] |
| *Yersinia pestis* | [203, 229] |
| Hemorrhagic fever with renal syndrome virus | [192, 193, 197, 200, 205, 214, 217, 230, 233, 245, 246, 254, 256, 262, 271, 280, 281, 305, 347] |
| Severe fever with thrombocytopenia syndrome virus | [316] |

**Table S3**: **The inclusion and exclusion criteria for screening articles**.

| **Criteria** | **Guidance** | **Outcome** |
| --- | --- | --- |
| Title/Abstract screening |  |  |
| #1: Mites or agents | Does the Title/Abstract refer the blood-sucking mites and mite-associated agents? | If Yes, remain and evaluate #2. If No, exclude. |
| #2: Source of investigation | Does the Title/Abstract refer the blood-sucking mites and mite-associated agents which are from natural environment? | If Yes, remain and evaluate #3. If No, exclude. |
| #3: Not review | Does the Title/Abstract refer the article which is Not a review without geographical information? (Not reviewing the published articles, with presenting new primary data) | If No, remain for full text review. If Yes, exclude. |
| Full text screening |  |  |
| #1: Re-screening | Does the article meet the screening criteria before? 1-blood-sucking mites and mite-associated agents  2-infection in natural environment 3-not review without geographical information 4-not insecticide, testing tool, drug or vaccine trials 5-not molecular research of mites or mite-associated agents | If Yes, remain and evaluate #2. If No, exclude. |
| #2: Testing | Does the article refer the specific detection methods of agents? 1-detailed mites specimen used for testing  2-agent-based testing method (e.g. serological or molecular) 3-specific agent identified in the detection | If Yes, remain and evaluate #3. If No, exclude from database of agents. |
| #3: Geographical information | Does the article refer the geographical information? 1-geographic location information at province, city or county administrative divisions levels 2-exact locations or only marked the latitude and longitude | If Yes, remain for data extracting. If No, exclude. |

**Table S4**: **Clustering analysis of eco-climatic predictors at the county level based on pairwise Pearson correlation coefficients.** Pairwise correlations above 0.8 are shown, and blank off-diagonal cells all have correlations < 0.8. Predictors grouped to the same cluster are colored the same. From each cluster, only one predictor (marked with ^a^) is chosen to be used in county-level BRT models to avoid multicollinearity.

|  | bio1 ^a^ | bio2 ^a^ | bio3 ^a^ | bio4 ^a^ | bio5 ^a^ | bio6 | bio7 | bio8 | bio9 | bio10 | bio11 | bio12 ^a^ | bio13 | bio14 | bio15 ^a^ | bio16 | bio17 ^a^ | bio18 | bio19 |
| --- | --- | --- | --- | --- | --- | --- | --- | --- | --- | --- | --- | --- | --- | --- | --- | --- | --- | --- | --- |
| bio1 |  |  |  |  |  | 0.967 |  |  | 0.962 |  | 0.965 |  |  |  |  |  |  |  |  |
| bio2 |  |  |  |  |  |  |  |  |  |  |  |  |  |  |  |  |  |  |  |
| bio3 |  |  |  |  |  |  |  |  |  |  |  |  |  |  |  |  |  |  |  |
| bio4 |  |  |  |  |  |  | 0.967 |  |  |  |  |  |  |  |  |  |  |  |  |
| bio5 |  |  |  |  |  |  |  | 0.815 |  | 0.880 |  |  |  |  |  |  |  |  |  |
| bio6 |  |  |  |  |  |  |  |  | 0.965 |  | 0.992 |  |  |  |  |  |  |  |  |
| bio7 |  |  |  |  |  |  |  |  |  |  |  |  |  |  |  |  |  |  |  |
| bio8 |  |  |  |  |  |  |  |  |  | 0.934 |  |  |  |  |  |  |  |  |  |
| bio9 |  |  |  |  |  |  |  |  |  |  | 0.975 |  |  |  |  |  |  |  |  |
| bio10 |  |  |  |  |  |  |  |  |  |  |  |  |  |  |  |  |  |  |  |
| bio11 |  |  |  |  |  |  |  |  |  |  |  |  |  |  |  |  |  |  |  |
| bio12 |  |  |  |  |  |  |  |  |  |  |  |  | 0.961 |  |  | 0.973 |  | 0.893 |  |
| bio13 |  |  |  |  |  |  |  |  |  |  |  |  |  |  |  | 0.993 |  | 0.950 |  |
| bio14 |  |  |  |  |  |  |  |  |  |  |  |  |  |  |  |  | 0.970 |  | 0.924 |
| bio15 |  |  |  |  |  |  |  |  |  |  |  |  |  |  |  |  |  |  |  |
| bio16 |  |  |  |  |  |  |  |  |  |  |  |  |  |  |  |  |  | 0.955 |  |
| bio17 |  |  |  |  |  |  |  |  |  |  |  |  |  |  |  |  |  |  | 0.981 |
| bio18 |  |  |  |  |  |  |  |  |  |  |  |  |  |  |  |  |  |  |  |
| bio19 |  |  |  |  |  |  |  |  |  |  |  |  |  |  |  |  |  |  |  |

**Table S5**: **The social, environmental and ecoclimatic variables used for ecological modeling for mite species and mite-borne pathogens at county level in this study.**

| **Category** | **Variable** | **Description** | **Resolution** |
| --- | --- | --- | --- |
| **Ecoclimatic** | BIO01^a^ | Annual mean temperature (℃) | Station |
|  | BIO02 | Mean diurnal range (Mean of monthly (max temp-min temp)) (℃) | Station |
|  | BIO03 | Isothermally (BIO02/BIO07) (*100) | Station |
|  | BIO04 | Temperature seasonality (standard deviation*100) | Station |
|  | BIO05 | Max temperature of warmest month (℃) | Station |
|  | BIO06 ^a^ | Min temperature of coldest month (℃) | Station |
|  | BIO07 | Annual range of temperature (BIO05-BIO06) (℃) | Station |
|  | BIO08 ^a^ | Mean temperature of wettest quarter (℃) | Station |
|  | BIO09 ^a^ | Mean temperature of driest quarter (℃) | Station |
|  | BIO10 | Mean temperature of warmest quarter (℃) | Station |
|  | BIO11 ^a^ | Mean temperature of coldest quarter (℃) | Station |
|  | BIO12 ^a^ | Annual precipitation (mm) | Station |
|  | BIO13 ^a^ | Precipitation of wettest month (mm) | Station |
|  | BIO14 | Precipitation of driest month (mm) | Station |
|  | BIO15 ^a^ | Precipitation seasonality (Coefficient of variation) | Station |
|  | BIO16 | Precipitation of wettest quarter (mm) | Station |
|  | BIO17 ^a^ | Precipitation of driest quarter (mm) | Station |
|  | BIO18 ^a^ | Precipitation of warmest quarter (mm) | Station |
|  | BIO19 | Precipitation of coldest quarter (mm) | Station |
| **Environmental** | Plain | Presence of plain | County |
|  | Basin | Presence of basin | County |
|  | Mountain land | Presence of mountain land | County |
|  | Hill | Presence of hill | County |
|  | Plateau | Presence of plateau | County |
|  | Paddy Field | Percentage coverage of paddy field (1%) | 1 km*1 km |
|  | Rainfed Cropland | Percentage coverage of rainfed cropland (1%) | 1 km*1 km |
|  | Forest | Percentage coverage of forest (1%) | 1 km*1 km |
|  | Spinney | Percentage coverage of spinney (1%) | 1 km*1 km |
|  | Open Woodland | Percentage coverage of open woodland (1%) | 1 km*1 km |
|  | Other Woodland | Percentage coverage of other woodland (1%) | 1 km*1 km |
|  | High Coverage Grasslands | Percentage coverage of high coverage grassland (1%) | 1 km*1 km |
|  | Moderate Coverage Grasslands | Percentage coverage of moderate coverage grasslands (1%) | 1 km*1 km |
|  | Low Coverage Grasslands | Percentage coverage of low coverage grasslands (1%) | 1 km*1 km |
|  | River | Percentage coverage of river (1%) | 1 km*1 km |
|  | Lake | Percentage coverage of lake (1%) | 1 km*1 km |
|  | Reservoir | Percentage coverage of reservoir (1%) | 1 km*1 km |
|  | Permanent Glacial Snow | Percentage coverage of permanent glacial snow (1%) | 1 km*1 km |
|  | Mud Flat | Percentage coverage of mud flat (1%) | 1 km*1 km |
|  | Shoaly Land | Percentage coverage of shoaly land (1%) | 1 km*1 km |
|  | Rural Residential Land | Percentage coverage of rural residential land (1%) | 1 km*1 km |
|  | Other Construction Land | Percentage coverage of other construction land (1%) | 1 km*1 km |
|  | Sand | Percentage coverage of sand (1%) | 1 km*1 km |
|  | Gobi | Percentage coverage of gobi (1%) | 1 km*1 km |
|  | Saline And Alkaline Land | Percentage coverage of saline and alkaline land (1%) | 1 km*1 km |
|  | Marsh Land | Percentage coverage of marsh land (1%) | 1 km*1 km |
|  | Bare Land | Percentage coverage of bare land (1%) | 1 km*1 km |
|  | Bare Exposed Rock Or Gravel | Percentage coverage of bare exposed rock or gravel (1%) | 1 km*1 km |
|  | Mammalian Richness | Number of mammalian species per square kilometre | 1 km*1 km |
|  | Elevation | Average elevation (m) | 1 km*1 km |
| **Economic** | GDP | Real GDP per capita (100 million) | County |
| **Demographic** | Population Density | People per square kilometre | County |
| **Mite Distribution** | *L. deliense* | Predicted spatial distribution of L. deliense (1%) | County |
|  | *L. fuji* | Predicted spatial distribution of L. fuji (1%) | County |
|  | *L. intermedium* | Predicted spatial distribution of L. intermedium (1%) | County |
|  | *Od. majesticus* | Predicted spatial distribution of Od. majesticus (1%) | County |
|  | *L. rubellum* | Predicted spatial distribution of L. rubellum (1%) | County |
|  | *L. scutellare* | Predicted spatial distribution of L. scutellare (1%) | County |
|  | *L. palpale* | Predicted spatial distribution of L. palpale (1%) | County |
|  | *As. indica* | Predicted spatial distribution of As. indica (1%) | County |
|  | *L. yui* | Predicted spatial distribution of L. yui (1%) | County |

^a^ Used as predictors in the logistic model for selection of counties for mite survey. The output sampling probabilities of counties (after taking reciprocal) were used as weights for the BRT models for the 21 blood-sucking mite species.

**Table S6**: **BRT-model-estimated mean (standard deviation) relative contributions of top environmental and ecoclimatic factors (RC≥5%) to the spatial distribution of the Cluster Ⅰ.** Mean AUCs (95% percentiles) and partial area AUC ratio (calculated at tolerance level of 0.2) are given.

| **Category** | **Variable** | ***Leptotrombidium yui*** | ***Leptotrombidium scutellare*** | ***Ornithonyssus bacoti*** | ***Odontacarus majesticus*** | ***Leptotrombidium deliense*** |
| --- | --- | --- | --- | --- | --- | --- |
| **Ecoclimatic** | Annual Mean Temperature | 6.84 (2.27) | 7.11 (1.60) | 6.13 (1.74) | 7.50 (3.04) | 32.92 (6.08) |
|  | Mean Diurnal Range |  |  | 5.97 (1.85) | 8.41 (3.70) |  |
|  | Isothermality | 7.85 (2.95) |  |  |  |  |
|  | Temperature Seasonality | 7.57 (2.40) |  |  |  | 10.28 (2.06) |
|  | Max Temperature of Warmest Month | 5.34 (1.92) |  |  |  |  |
|  | Total precipitation |  |  |  | 10.95 (2.78) | 12.76 (4.58) |
|  | Precipitation of Driest Quarter | 13.44 (2.48) | 23.67 (3.13) |  |  |  |
| **Environmental** | Elevation |  |  |  | 7.79 (2.27) |  |
|  | Mammalian Richness | 12.48 (3.02) | 6.98 (1.61) | 13.91 (2.78) | 9.46 (2.09) |  |
|  | Paddy Field | 6.43 (2.42) |  | 6.55 (1.76) | 5.76 (1.71) |  |
|  | Rainfed Cropland |  |  | 5.16 (1.38) |  |  |
|  | Spinney |  |  | 6.41 (1.69) |  | 5.00 (1.51) |
|  | High Coverage Grasslands |  |  | 6.84 (1.45) | 5.52 (1.83) |  |
|  | Reservoir | 6.23 (2.04) |  |  | 11.59 (3.03) | 5.41 (1.56) |
|  | Bare Exposed Rock Or Gravel | 5.14 (2.14) |  |  |  |  |
| **Other** | GDP |  | 5.46 (1.81) | 4.95 (1.54) |  |  |
|  | Population Density |  |  | 5.99 (1.58) |  |  |
| **AUC** | Train | 0.995 (0.988‒0.999) | 0.991 (0.979‒0.998) | 0.978 (0.958‒0.993) | 0.993 (0.988‒0.997) | 0.983 (0.976‒0.992) |
|  | Test | 0.891 (0.807‒0.951) | 0.847 (0.784‒0.896) | 0.764 (0.693‒0.832) | 0.927 (0.888‒0.962) | 0.921 (0.879‒0.950) |
| **Partial AUC Ratio** | Train | 1.89 | 1.82 | 1.75 | 1.84 | 1.73 |
|  | Test | 1.62 | 1.41 | 1.26 | 1.69 | 1.56 |

**Table S7**: **BRT-model-estimated mean (standard deviation) relative contributions of top environmental and ecoclimatic factors (RC≥5%) to the spatial distribution of the Cluster** **Ⅱ.** Mean AUCs (95% percentiles) and partial area AUC ratio (calculated at tolerance level of 0.2) are given.

| **Category** | **Variable** | ***Leptotrombidium intermedium*** | ***Leptotrombidium fuji*** | ***Leptotrombidium rubellum*** | ***Ascoschoengastia indica*** | ***Tricholaelaps myonysognathus*** | ***Laelaps***  ***nuttalli*** |
| --- | --- | --- | --- | --- | --- | --- | --- |
| **Ecoclimatic** | Annual Mean Temperature |  |  |  |  | 12.05 (2.42) | 5.26 (1.10) |
|  | Isothermality | 8.63 (4.18) | 9.88 (4.74) | 19.14 (6.49) | 5.30 (2.40) |  |  |
|  | Temperature Seasonality | 7.71 (2.47) | 7.52 (2.45) | 8.35 (2.99) |  |  |  |
|  | Max Temperature of Warmest Month | 7.10 (2.34) | 8.09 (2.48) |  |  |  |  |
|  | Precipitation Seasonality |  | 7.88 (2.92) | 5.63 (2.80) |  | 5.59 (1.47) |  |
|  | Precipitation of Driest Quarter | 7.59 (2.29) | 8.50 (2.50) | 5.28(2.50) |  |  |  |
| **Environmental** | Elevation | 5.45 (2.09) |  |  | 7.42 (3.16) |  |  |
|  | Mammalian Richness | 14.68 (5.06) | 14.99 (4.97) | 26.10 (6.94) | 44.70 (6.51) | 9.80 (2.36) | 17.60 (3.25) |
|  | Paddy Field |  |  |  |  |  | 5.25 (1.39) |
|  | Forest | 5.52 (1.98) | 8.85 (3.04) |  |  |  | 8.34 (2.40) |
|  | Spinney | 9.11 (2.59) |  |  |  |  |  |
|  | Open Woodland |  |  |  |  | 8.50 (2.11) |  |
|  | Other Woodland |  |  |  | 7.56 (3.45) | 6.19 (1.66) |  |
|  | High Coverage Grasslands |  |  |  | 5.25 (2.39) |  |  |
|  | Low Coverage Grasslands |  |  |  |  | 8.37 (2.12) |  |
|  | Shoaly Land |  | 7.22 (2.53) |  | 6.32 (2.61) |  |  |
|  | Bare Exposed Rock Or Gravel | 6.67 (3.21) | 6.07 (2.52) |  |  |  |  |
| **AUC** | Train | 0.976 (0.959‒0.989) | 0.995 (0.989‒1.000) | 0.999 (0.998‒1.000) | 0.995 (0.992‒0.999) | 0.992 (0.982‒0.999) | 0.973 (0.954‒0.988) |
|  | Test | 0.784 (0.666‒0.890) | 0.891 (0.825‒0.944) | 0.953 (0.899‒0.985) | 0.919 (0.845‒0.972) | 0.851 (0.809‒0.901) | 0.808 (0.759‒0.850) |
| **Partial AUC Ratio** | Train | 1.84 | 1.91 | 1.94 | 1.91 | 1.80 | 1.69 |
|  | Test | 1.28 | 1.62 | 1.80 | 1.66 | 1.46 | 1.33 |

**Table S8**: **BRT-model-estimated mean (standard deviation) relative contributions of top environmental and ecoclimatic factors (RC≥5%) to the spatial distribution of the Cluster Ⅲ.** Mean AUCs (95% percentiles) and partial area AUC ratio (calculated at tolerance level of 0.2) are given.

| **Category** | **Variable** | ***Hypoaspis***  ***lubrica*** | ***Hirstionyssus isabellinus*** | ***Haemolaelaps glasgowf*** | ***Laelaps***  ***jettmari*** | ***Eulaelaps***  ***stabularis*** |
| --- | --- | --- | --- | --- | --- | --- |
| **Ecoclimatic** | Annual Mean Temperature | 6.57 (2.11) | 15.99 (6.43) | 8.04 (2.08) |  | 7.91 (1.73) |
|  | Mean Diurnal Range | 6.71 (1.98) |  |  |  | 5.15 (1.21) |
|  | Isothermality | 5.47 (1.36) | 5.47 (2.55) |  |  |  |
|  | Temperature Seasonality |  | 16.36 (7.39) | 17.78 (2.66) | 9.96 (1.45) | 6.85 (1.64) |
|  | Total precipitation |  |  |  | 8.55 (1.60) |  |
|  | Precipitation of Driest Quarter |  |  | 5.07 (1.54) | 6.61 (1.34) | 13.57 (1.76) |
| **Environmental** | Elevation |  |  |  | 6.36 (1.56) |  |
|  | Mammalian Richness | 6.23 (1.65) | 9.55 (3.84) | 5.45 (0.96) |  | 5.07 (1.09) |
|  | Rainfed Cropland | 6.52 (1.73) |  |  |  | 5.62 (1.27) |
|  | Forest |  | 6.43 (2.56) |  |  |  |
|  | Spinney |  |  |  |  | 5.48 (1.21) |
|  | High Coverage Grasslands | 5.08 (1.46) |  |  |  | 5.58 (1.23) |
|  | Moderate Coverage Grasslands | 9.97 (2.13) | 13.10 (5.34) |  | 7.19 (1.34) |  |
|  | Reservoir | 5.54 (1.64) |  |  |  |  |
|  | Shoaly Land |  |  | 5.90 (1.32) |  |  |
| **AUC** | Train | 0.982 (0.965‒0.995) | 0.978 (0.964‒0.990) | 0.972 (0.949‒0.990) | 0.984 (0.971‒0.996) | 0.974 (0.953‒0.992) |
|  | Test | 0.804 (0.717‒0.862) | 0.815 (0.698‒0.896) | 0.779 (0.740‒0.819) | 0.816 (0.771‒0.874) | 0.774 (0.729‒0.825) |
| **Partial AUC Ratio** | Train | 1.78 | 1.85 | 1.52 | 1.65 | 1.62 |
|  | Test | 1.33 | 1.28 | 1.21 | 1.32 | 1.28 |

**Table S9**: **BRT-model-estimated mean (standard deviation) relative contributions of major environmental and ecoclimatic factors (RC≥5%) to the spatial distribution of the Cluster Ⅳ.** Mean AUCs (95% percentiles) and partial area AUC ratio (calculated at tolerance level of 0.2) are given.

| **Category** | **Variable** | ***Hirstionyssus sunci*** | ***Laelaps echidninus*** | ***Eulaelaps shanghaiensis*** | ***Hypoaspis pavlovskii*** |
| --- | --- | --- | --- | --- | --- |
| **Ecoclimatic** | Annual Mean Temperature | 5.03 (1.56) | 10.47 (1.75) | 5.03 (2.25) | 10.01 (2.50) |
|  | Max Temperature of Warmest Month |  |  |  | 5.71 (1.31) |
|  | Total precipitation |  | 12.12 (1.95) |  |  |
|  | Precipitation Seasonality |  |  |  |  |
|  | Precipitation of Driest Quarter | 9.34 (2.53) |  | 8.52 (2.81) |  |
| **Environmental** | Elevation | 6.89 (1.68) |  | 11.86 (2.52) | 9.21 (2.08) |
|  | Mammalian Richness | 7.87 (1.85) | 12.28 (2.49) | 5.63 (1.45) |  |
|  | Rainfed Cropland | 6.65 (1.35) |  | 11.09 (2.05) | 7.02 (1.94) |
|  | Spinney |  | 7.20 (2.16) | 6.71 (1.81) |  |
|  | Open Woodland | 7.25 (1.65) |  | 6.16 (1.80) | 7.08 (1.76) |
|  | Other Woodland |  |  | 7.25 (2.64) |  |
|  | High Coverage Grasslands |  |  | 13.45 (3.21) |  |
|  | Moderate Coverage Grasslands | 13.28 (1.94) | 8.49 (1.71) | 12.47 (1.75) | 13.93 (2.66) |
|  | Low Coverage Grasslands | 5.27 (1.45) |  |  |  |
|  | Rural Residential Land |  |  | 5.28 (2.21) | 5.42 (1.38) |
| **AUC** | Train | 0.980 (0.961‒0.994) | 0.955 (0.930‒0.980) | 0.995 (0.987‒0.999) | 0.967 (0.944‒0.986) |
|  | Test | 0.785 (0.720‒0.846) | 0.784 (0.733‒0.833) | 0.859 (0.772‒0.938) | 0.796 (0.734‒0.849) |
| **Partial AUC Ratio** | Train | 1.75 | 1.55 | 1.87 | 1.68 |
|  | Test | 1.26 | 1.26 | 1.44 | 1.27 |

**Supplementary References**

1. Yao HW, Wang YX, Mi XM, Sun Y, Liu K, Li XL, et al. The scrub typhus in mainland China: spatiotemporal expansion and risk prediction underpinned by complex factors. Emerg Microbes Infect 2019; 8: 09–19.

2. Elliott I, Pearson I, Dahal P, Thomas NV, Roberts T, Newton PN. Scrub typhus ecology: a systematic review of *Orientia* in vectors and hosts. Parasit Vectors 2019; 12: 513.

3. Matthee S, Stekolnikov AA, Mescht LV, Froeschke G, Morand S. The diversity and distribution of chigger mites associated with rodents in the South African savanna. Parasitology 2020; 147: 038–47.

4. Acharya BK, Chen W, Ruan Z, Pant GP, Yang Y, Shah LP, et al. Mapping environmental suitability of scrub typhus in Nepal using Maxent and Random Forest Models. Int J Environ Res Public Health 2019; 16: 3.

5. Yu X, Ye RY, Cao HL, Xiao H, Chen X, Ruqi ZR, et al. Scrub typhus in Jiangsu Province, China: epidemiologic features and spatial risk analysis. BMC Infect Dis 2018; 18: 372.

6. Amiri M, Tarkesh M, Jafari R, Jetschke G. Bioclimatic variables from precipitation and temperature records vs. remote sensing-based bioclimatic variables: Which side can perform better in species distribution modeling? Ecol Inform 2020; 7.

7. O'Donnell MS, Ignizio DA. Bioclimatic predictors for supporting ecological applications in the conterminous United States. U.S. Geological Survey Data Series 2012; 10p.

8. Hamilton LC, Center DR. Statistics with Stata. California: Duxbury Press; 1992.

9. Zhao GP, Wang YX, Fan ZW, Ji Y, Liu MJ, Zhang WH, et al. Mapping ticks and tick-borne pathogens in China. Nature communications 2021; 12: 1075.

10. Chen XB. Survey of chigger mites on rats in southern Kiangsu province with descriptions of two new species (acariformes: Trombiculidae). Insect Science 1978; 2: 197–203. (in Chinese)

11. Fan PF, Chen DX, Zhang ZH. Gamasid mites in Anhui province-Ⅰ. Anhui indoor rodents leather mite (abstract). Academic Medicine Bengbu 1981; 3: 220. (in Chinese)

12. Yao WB, Chen GD. Studies on larva of chigger mites in Inner Mongolia. The medical report 1981; 1: 31–44. (in Chinese)

13. Chen XB, Liu JR. Studies on the fauna of chigger mites in southern Anhui and Bengbu. Journal of Bengbu Medical College 1982; 1: 25–28.

14. Wang SQ, Liang LS, Guo KY, Yu ES. Investigation on the foci of scrub typhus in winter. Fujian Journal of Medicine 1983; 6: 23–24. (in Chinese)

15. Yu ZZ. Studies on chigger mites from Yunnan and a new subgenus. Yunnan medicine 1983; 6: 377–384. (in Chinese)

16. Lee GM. A New Species of *Gahrliepia* (Acari: Trombiculidae) from a Shrew-Hedgehog Insectivore in China. Journal of Medical Entomology 1984; 21.

17. Fan RS, Zhang Y, Tan JM, Pan QM, Huang Q, Li XS, et al. Epidemiology and ecology of rickettsia diseases in the People's Republic of China. Rev Infect Dis 1987; 9: 823–840. (in Chinese)

18. Wei JJ, Tong GZ, Shi SF. Study on *Leptotrombidium Insularae*-a new vector of tsutsugamushi diseases in China. PLA Medical Journal 1987; 6: 415–418. (in Chinese)

19. Yang BY, Cai HQ. A report of investigation on mouse and Its Ectozal Parasite of SanDu Area. Jinan Medical Journal (Medical Special Edition) 1988; 4: 51–55. (in Chinese)

20. Su DM, Jiang RJ, Wang ZD, Wang BX, Ji GD, Yang LB, et al. Investigation report on the epidemic of tsutsugamushi in Dongtai city. Jiangsu medicine 1989; 5: 239–241. (in Chinese)

21. Wang DQ, Yu ZZ. Chigger mites of the genus *Leptotrombidium s.str.* in China with keys to species and their distribution. Endemic Diseases Bulletin 1989; 1: 45–56. (in Chinese)

22. Ma LM. A preliminary survey of gamasid mites in the northern Qinghai-Tibet Plateau. Sichuan Journal of Zoology 1990; 3: 12–13. (in Chinese)

23. Ye RY, Yu X, Cao HL, Chen XR. The fauna of ticks and medical mites in the Ertix River Valley and its neighboring mountainous areas in Xinjiang, China. Endemic Diseases Bulletin 1990; 4: 27–31. (in Chinese)

24. Yu X, Ye RY, Cao HL, Xiao H, Chen X, Ruqi ZR, et al. A report on the fauna of ticks and mites in Tacheng Emin Valley and its adjacent mountains. Endemic Bulletin 1990; 4: 23–26. (in Chinese)

25. Zhou PS. Survey of the natural foci of the Tsutsugamushi Disease in the Southwest Region of Guangxi. Chinese Journal of Vector Biology and Control 1991; 3: 206–209. (in Chinese)

26. Wang JL, Yang YF, Yao YC. Type and characteristice of epidemic focus of tsutsugamushi disease in Yimeng Mountain Area. Journal of Linyi Medical School 1991; 3: 235–237. (in Chinese)

27. Li WX. The ecogeographical distribution of gamasides in Liaoning Province. Acta Entomologica Sinica 1991; 1: 108–116. (in Chinese)

28. Cao XR, Wang JJ, Zhang YG, Wang YN, Yang CM, Xu ZP, et al. Recent studies on scrub typhus and *Rickettsia tsutsugamushi* in Shandong Province China. European Journal of Epidemiology 1991; 6 . (in Chinese)

29. Feng XG, Lei YM, Chen YM, Zhang HL. Antigen typing and serotyping of *Rickettsia tsutsugamushi* epidemic strains in Huaping County, Yunnan Province. Yunnan medicine 1992; 6: 368–369. (in Chinese)

30. Liao GP, Guo CH, Pan XY, Yang JZ, Zhang FS. Investigation of ticks and mites in vitro of domestic animals and poultry. Zhejiang Agricultural Sciences 1992; 6: 295–296. (in Chinese)

31. Wang DQ, Yu ZZ. Chigger mites of the genus *Leptotrombidium*: key to species and their distribution in China. Medical and veterinary entomology 1992; 6 . (in Chinese)

32. Wang DQ. *Laelaps jettmari* and its morphological difference in different districts of China (Acari: Laelapidae). Endemic Diseases Bulletin 1993; 3: 78–82. (in Chinese)

33. Zhang GD. Investigations on rodent and its ectoparasite in Guinan county, Qinghai Province. Chinese Journal of Vector Biology and Control 1993; 5: 355–358. (in Chinese)

34. Lu ZX, Hu LM, Cai ZL, Jin XT, Sun HL, Zheng XH, et al. Epidemiological investigation of *Tsutsugamushi* natural foci in Hunchun city. Public Health in China 1993; 5: 207–208. (in Chinese)

35. Yang GR, Yu ZZ, Gong ZD, Tao HH, Yang H, Wu YX, et al. A study on chigger mites in Xigu flatland district besides Lancan river. Chinese Journal of Vector Biology and Control 1993; 6: 428–430+491–492. (in Chinese)

36. Ye RY, Yu X, Zhang ZJ, Re ZW, Cao HL, Chen W, et al. Fauna and medical importance of acarine in Eastern Xinjiang. Endemic Diseases Bulletin 1993; 4: 100–104+143. (in Chinese)

37. Sun BY. The ecological and geographical distribution of the trombiculid mites in Liaoning Province. Acta Entomologica Sinica 1994; 1: 71–77. (in Chinese)

38. Liao HR. A summary of the investigation on *Rattus rattoides exiguus* and its ectoparasites in Fujian. Wuyi Science Journal 1994; 158–161. (in Chinese)

39. Huang XQ, Yang YM, Shen LJ, Guo XG. A list of gamasid mites parasitized on the body surface of small animals in Weishan county. Journal of Dali Medical College 1994; 2: 8–10+57. (in Chinese)

40. Lei YM, Feng XG, Chen YM, Zhang HL. Serum classification of *Rickettsia tsutsugamushi* in Yunnan Province. Chinese Journal of Zoonoses 1994; 2: 62–63. (in Chinese)

41. Liu JH, Long ZM, Yan AW, Huang JJ, Chen HY. An analysis on gamasid mites for ectoparasites of rodents in Hainan Province. Entomological Knowledge 1994; 4: 229–231. (in Chinese)

42. Mo YC, Pan GX. Preliminary investigation on rodents and parasitic fleas mites in southeast Guizhou province. Guizhou Medicine 1994; 4: 243–244. (in Chinese)

43. Wang ZB, Song JY. Distribution and geographical division of chigger mites in Jiangxi Province. Jiangxi Plant Protection 1994; 3: 1–5+18. (in Chinese)

44. Wu AG, Guo XG. An investigation of gamasid mites on small mammals in the flat areas of Gengma, Yunnan. Endemic Diseases Bulletin 1994; 1: 100. (in Chinese)

45. Wu AG, Guo XG. A Research on the Species Constitution and Interspecific Association of Gamasid Mite in Gengma, Yunnan. Journal of Dali Medical College 1994; 2: 36–38+60. (in Chinese)

46. Chi YD, Chen DR. An investigation on the gamasids from rodents in Sanming City Area. Fujian Animal Husbandry and Veterinary 1995; 3: 6–8. (in Chinese)

47. Yu ES. Epidemiological analysis on tsutsugamushi disease of islands in two coasts of the Taiwan Strait. Straits Journal of Preventive Medicine 1995; 1: 7–9. (in Chinese)

48. Gao XP, Bai XL, Ma LM. Study on mites of soil in Ningxia. Journal of Ningxia Agricultural College 1995; 3: 18–23. (in Chinese)

49. Ma LM. Five new records of gamasine mites from China (Acari:Mesostigmata). Acta Arachnologica Sinica 1995; 2: 159–160. (in Chinese)

50. Long LL, Chen Q, Qin ZS, Peng DH, Wu SQ, Yu XL, et al. Study on category and ecology of gamasides in Xiangxi Self Government. Practical preventive medicine 1995; 2: 68–71. (in Chinese)

51. Guo XG, Ye BH, Gu YM, Chen YM. Spatial distribution pattern of dominant gamasid mite population on the surface of *Rattus flavescens*. Medical animal control 1996; 3: 17–19. (in Chinese)

52. Zhang YZ, Deng CY, Wang DQ. Four new species of the genus *Leptotrombidium* from Xizang of China (Acari:Trombiculidae). Entomological Journal of East China 1996; 2: 10–16. (in Chinese)

53. Zhao Y, Zhang LN, Wang Y, Ma LM. Gamasid mites from Heilongjiang Province. Chinese Journal of Vector Biology and Control 1996; 6: 477–480. (in Chinese)

54. Zhou PS, Cai HZ, Shi JZ. Study on comprehensive prevention and control of tsutsugamushi disease in the Wuzhishan area of Hainan Province. Public Health in China 1996; 7: 304. (in Chinese)

55. Chen YM, Feng XG, Lei YM. Studies on Serotyping of *Rickettsia tsutsugamushi* in Yunnan. Chinese Journal of Zoonoses 1997; 1: 13–16. (in Chinese)

56. Shi YL, Gao XD, Liang XC, Yao CX, Ding XL, Xi JX, et al. The investigation and analysis of epidemic hemorrhagic fever in Minxian County, Gansu. Endemic Diseases Bulletin 1997; 2: 67–69+66. (in Chinese)

57. Gao GY, Wang ZC. Studies on serological typing of *Rickettsia tsutsugamushi* in Binchuan County of Yunnan. Chinese Journal of Vector Biology and Control 1998; 4: 53–54. (in Chinese)

58. Liu GP, Tao ZG, Wang CM. Faunal and ecological studies of gamasid mites in frontiers of northeast China. Chinese Journal of Vector Biology and Control 1998; 5: 19–21. (in Chinese)

59. She JJ, Zhang Y, Huang CA, Yu MM, Jiang KJ, Wu GH. Preliminary study on *Leptotrombidium* (*L*.) *subpalpale* as spreading medium of HFRS. Chinese Journal of Vector Biology and Control 1998; 1: 55–58. (in Chinese)

60. Cao SL, Xu LX, Wang GF, Wang YZ, Wang GM, Ma LM. Collection and records of gamasid mites in soil in Baicheng area. Chinese Journal of Vector Biology and Control 1998; 5: 9. (in Chinese)

61. Deng GL, Jiang W, Ye RY, Qiao YJ, Yu X. The fauna of ticks and mites in the Yeerqiang river valley in Xinjiang, China. Endemic Diseases Bulletin 1999; 3: 58–60. (in Chinese)

62. Guo XG, Qian TJ. Research on spatial pattern of gamasid mites from the mountains of Gaoligong and Dandanglika. Journal of Dali Medical College 1999; 2: 1–3. (in Chinese)

63. Wang QL. A preliminary list of the genus *Acari* from China. Journal of Chengde Medical College 1999; 1: 58–59. (in Chinese)

64. Wan XY, Zhong HP, Lu SP, He Q. Investigation on rodents and ectoparasites at Chenglingji port inYueyang. Practical Preventive Medicine 1999; 2: 64–65. (in Chinese)

65. Zhong YH. Collection report of chigger mites in some areas of Zhaoqing. Medical animal control 1999; 2: 93–94. (in Chinese)

66. Zhang LH, Zhang GD, Ma LM. The discovery of natural foci of tsutsugamushi disease in southern Shanxi Province and its zoo-epidemiologic characteristics. Journal of Parasite and Medical Entomology 1999; 3: 47–52. (in Chinese)

67. Hong J, Kong QA, Zhang BG, Jing RX. List of blood-sucking arthropods from Fengcheng, Liaoning. Medical animal control 2000; 1: 53. (in Chinese)

68. Jiang PL, Huang JL, Peng GF, Liu JH, Zhu SF, Wang ZB, et al. The confirmation of the epidemic area of tsutsugamushi disease on Nan 'ao county in China. Chinese Journal of Zoonoses 2000; 6: 24–27. (in Chinese)

69. Jian S, Zhou LQ, Chen SL, Li CM, Kong LY, Gang ZW, et al. Investigation on the vector and host of tsutsugamushi disease in epidemic focus, Taihang Mountains, Hebei Province. Chinese Journal of Vector Biology and Control 2000; 6: 462–465. (in Chinese)

70. Yang ZQ, Liu YX, Yu XM, Wu QY, Xing RY. Investigation on natural foci of autumn-winter type tsutsugamushi disease in Shandong province. Chinese Journal of Epidemiology 2000; 4: 43–46. (in Chinese)

71. Yuan GL, Zheng ZJ, Chen WJ, Li XY, Li FP. Epidemiological investigation of tsutsugamushi disease in Ningde region from 1997 to 1998. Chinese Journal of Zoonoses 2000; 6: 109. (in Chinese)

72. Huang JL, Wang SS, Zhu SF, Jiang PL, Zeng NH, Wang ZB. The community composition and distribution of murine-like animals and their ectoparasites on Nanao and Nanpenglie Islands. Chinese Journal of Vector Biology and Control 2001; 3: 177–181. (in Chinese)

73. Wu HX, Zhang LP, Xue JZ, Yao JL. Effect of malachite green and temperature to *daphnia longispina*. Sichuan Journal of Zoology 2001; 4: 198–201. (in Chinese)

74. Zhang LH, Zhang GD, Ma LM. Collection and new records of gamasid mites in qinghai. Chinese Journal of Vector Biology and Control 2001; 3: 185. (in Chinese)

75. Li ZJ, Liu YR, Dong MJ, Liu LP, Xiang HY. A surey on gamasid mites and trombiculid mites with delimitation of zoogeographical regions in Yichang, Hubei Province. Chinese Journal of Vector Biology and Control 2002; 4: 279–281. (in Chinese)

76. Zheng Y, Yang XZ, Li HL, Wei SZ. List of gamasid mites in Qinghai Province. Endemic Diseases Bulletin 2002; 2: 71–74. (in Chinese)

77. Zhou CH, Lv GY, Qian SB. Distribution of rodents and vectors in natural foci of plague in Jianping county. Chinese Journal of Endemic Disease Control 2002; 3: 191. (in Chinese)

78. Zu WG, Li CM, Chen SL, Guo YX, Kong LY, SHI ZL. Investigation of tsutsugamushi foci in Taihang mountain area of Hebei Province. Chinese Journal of Parasitic Diseases Control 2002; 4: 73–74. (in Chinese)

79. Cui SQ, Zhang YF, Wang BL, Fu MC. Comparative analysis of gamasid mite detection in surrounding environment and imported goods at Ji 'an port. Chinese Journal of Vector Biology and Control 2003; 3: 220–221. (in Chinese)

80. Fan RS, Zhang Y, Tan JM, Pan QM, Huang Q, Li XS, et al. Baseline survey on taxonomy and population fluctuation of murine animals and their ectoparasites: forecast research on the related diseases of natural focus in Maoming Port. Chinese Journal of Vector Biology and Control 2003; 5: 364–367. (in Chinese)

81. Guo TY, Xu RM. Study on time niche among the ectoparasites of the rodent in Dongling Mountain in Beijing. Chinese Journal of Vector Biology and Control 2003; 1: 30–32. (in Chinese)

82. Hu Y, Ma LM. Collection of gamasid mites from Zhenjiang city and new records in Jiangsu Province, with descriptions of nymphs of three species (*Acari*). Entomolog ical Journal of East China 2003; 2: 115–119. (in Chinese)

83. Liu GP, Li DL, Chen CT, Liu XZ. Chigger mites from three provinces of northeast China. Chinese Journal of Vector Biology and Control 2003; 6: 444–446. (in Chinese)

84. Yuan GL, Chen WJ, Li XY, Li FP. Geographical epidemiology of *Tsutsugamushi* in Ningde City. Chinese Journal of Vector Biology and Control 2003; 5: 372–374. (in Chinese)

85. Liu YR, Yang ZQ. A preliminary list of gamasid mites in Hubei Province. Acta Arachnologica Sinica 2004; 1: 57–63. (in Chinese)

86. Xue J, Zhou GZ, Liu YX. The faunal study of chigger mites in Shandong Province. Chinese Journal of Vector Biology and Control 2004; 6: 452–454. (in Chinese)

87. Ma Y, Yang XZ, Tang XY. A new species of the genus *Eulaelaps* from Qinghai Province, China (Acari, Mesostigmata, Laelapidae). Acta Zootaxonomica Sinica 2005; 2: 355–357. (in Chinese)

88. Meng XX, Li HF, Zhang ZH. Investigation on the population distribution and seasonal fluctuation of gamasid mites in Dezhou City. Chinese Journal of Vector Biology and Control 2005; 5: 62–63. (in Chinese)

89. Niu AQ, Men XY, Dong WG, Qian TJ, Bao HE, Guo XG. Investigation on the species of chigger mites in surrounding regions of Erhai Lake in Dali, Yunnan. Chinese Journal of Parasitic Diseases Control 2005; 5: 11–15. (in Chinese)

90. Wang YY, Liu GP, Li Do, Chen CT. Study of species composition on the ectoparasites of the rodent in frontiers of Korea, Russia and northeast China. Chinese Journal of Vector Biology and Control 2005; 1: 44–46. (in Chinese)

91. Jiang HJ, Cai GZ, Su CJ, Huang WX. The investigation report of Trombiculid background at Xiaocuo Port. Chinese Frontier Health Quarantine 2006; 4: 218–220. (in Chinese)

92. Luo LP, Guo XG. Species investigation on ectoparasitic gamasid mites on *Rattus flavipectus* from 25 counties in Yunnan of China. Chinese Journal of Vector Biology and Control 2006; 5: 395–398. (in Chinese)

93. Niu AQ, Guo XG, Men XY. Investigation of ectoparasitic chigger mites on *Eothenomys miletus* in Dali of Yunnan. Journal of Tropical Medicine 2006; 2: 145–148. (in Chinese)

94. Zhang CH, Liu JR, Ye YL. Investigation on *Tsutsugamushi foci* in a reservoir area in eastern Fujian. Chinese Journal of Vector Biology and Control 2006; 5: 352. (in Chinese)

95. Lin JZ, Ma LM, Zhang YX, Ji J, Chen Xi. Investigation of free living gamasid mite in China (Acari:Gamasina). Wuyi Science Journal 2007; 120–154. (in Chinese)

96. Luo LP, Guo XG. Primary analysis of ectoparasitic gamasid mites on *Apodemus chevrieri*. Chinese Journal of Zoonoses 2007; 8: 844–847. (in Chinese)

97. Wang DJ, Wang CQ, Li ZJ, Zhou XC. A list of chigger mites and gamasid mites in Xingshan County of the Three Gorges Reservoir. Journal of Pathogen Biology 2007; 1: 80–81. (in Chinese)

98. Yin XP, Peng DX, Li D. Investigation on the fauna of rodent parasitic mite in Alashan Pass port area. Chinese Journal of Frontier Health and Quarantine 2007; 2: 93–95. (in Chinese)

99. Zhao XZ, Zhang Y. Investigation of rodent density and ectoparasites in Hainan province. Chinese Journal of Vector Biology and Control 2007; 6: 519–521. (in Chinese)

100. Zhong HP, Wan XY, Lu SP, Wang FJ. Investigation on vector organisms at Changsha international airport. Chinese Frontier Health Quarantine 2007; 2: 83–89. (in Chinese)

101. Ding LM, Xu YX. List of rodents and in vitro parasites in Shanghai Pudong international airport. Chinese Journal of Vector Biology and Control 2008; 2: 174. (in Chinese)

102. Lin JZ, Zhang YX, Ji J, Chen X, Sun L, Zhai TY, et al. Investigation of free living gamasid mites in China (II) (Acari: Mesostigmata). Wuyi Science Journal 2008; 24: 28–40. (in Chinese)

103. Wang SS, Huang JL, Su JX, Peng GF, Wang Y, Li MM. Epidemiological study of tsutsugamushi disease in Naozhou Island of Guangdong Province. Journal of Tropical Medicine 2008; 1: 58–60. (in Chinese)

104. Zhou SH, Li SY, Chen Wj. Investigation on ectoparasitic mites on rodents in Sanduao island of Fujian province. Chinese Journal of Vector Biology and Control 2008; 19: 546–549. (in Chinese)

105. Bai XL, Yan LM, Wu XL, Wei H, Qi RJ. Composition and distribution and harm of Gamasid mites in NingxiaHui Autonomous Region. Medical animal control 2009; 25: 487–493. (in Chinese)

106. Guo TY, Che ZJ, Liu YY, Cao JZ, Geng HS, Sun JL, et al. Surveillance of ectozoa on the body of rodent and hedge pig in Beijing ports. Chinese Journal of Vector Biology and Control 2009; 20: 27–29. (in Chinese)

107. Hong J, Zhang HW. List of known medical insects in Fengcheng. Medical animal control 2009; 25: 512–514. (in Chinese)

108. Lin JZ, Zhang YX, Ji J, Chen X, Sun L, Zhai TY, et al. Investigation of free living gamasid mites in China (III) (Acari: Mesostigmata). Wuyi Science Journal 2009; 25: 4–8. (in Chinese)

109. Liu XQ, Zhang Y, Su C, Tang Y, Shi S. Changchun City in 2008 rat body fieas, mites survey report. Chinese Journal of Endemic Disease Control 2009; 24: 292–293. (in Chinese)

110. Liu Y, Wang DM. Background investigation of medical vectors in Xinyuan Shipyard. Straits Journal of Preventive Medicine 2009; 15: 61–62. (in Chinese)

111. Xue XN, Hou W, Sun BJ. Investigation on medical vectors at Qingdao International Airport. Chinese Frontier Health Quarantine 2009; 32: 22–26+34. (in Chinese)

112. Yi HQ, Ye RY, Xue GH. Investigation on rodents and their ectoparasites at Xinjiang Wulasitai Port. Chinese Frontier Health Quarantine 2009; 32: 375–377. (in Chinese)

113. Yi Y. Classification of main gamasid mites on the body surface of rodents in Yunnan province. Master thesis: Guizhou University; 2009. (in Chinese)

114. Bei NX, Zhou X, Chen WP. A new species of *Cheiroseius* and a newly recorded species of podocinum from China (Acari, Mesostigmata, Aceosejidae, Podocinidae). Acta Zootaxonomica Sinica 2010; 35: 262–265. (in Chinese)

115. Lin JZ, Ma LM, Zhang YX, Ji J, Chen Xi. Investigation of free living gamasid mite in Henan, China (IV) (Acari: Mesostigmata). Wuyi Science Journal 2010; 26: 1–10. (in Chinese)

116. Liu GP, Ren QM, Xing AH, Wang F, Liu JQ. Investigation on *Rattus norvegicus* and its ectoparasites from frontiers of northeast China. Chinese Health Insecticide Equipment 2010; 16: 196–198. (in Chinese)

117. Su HY, Gao XP, Bai XL. Investigation on Parasitic Gamasid on *Phrynocephalus przewalskii* (Strauch, 1876) in Ningxia. Endemic Diseases Bulletin 2010; 25: 4–5. (in Chinese)

118. Xin ZW, Bai XL, Rao R. Investigations on Mesostigmatic Mites from Yanchi, Ningxia, China (1) Description of Deutonymph Hypoaspis (Cosmolaelaps). ENDEMIC DISEASES BULLETIN 2010; 25: 1–3+8. (in Chinese)

119. Zhang Y, Wu GH, Deng XZ, Wang ZC, Cao GW. Relationship between geographic characteristics and prevalence of tsutsugamushi disease in southeastern coastal areas of China. Public Health in China 2010; 26: 174–175. (in Chinese)

120. Liu ZJ, Zhang JJ. Small-sinica of Hirstionyssinae Evans et Till and *Myonyssinae Tiraboschi* in northwest China. Chin J Hyg Insect ＆ Equip 2011; 17: 132–134. (in Chinese)

121. Liu ZJ, Zhang JJ. List of four kinds of Acaridae in Northwest China. Chin J Hyg Insect＆Equip 2011; 17: 373–375. (in Chinese)

122. Liu ZJ, Zhang JJ. Small-sinica of Hypoaspidinae Vitzthum and Haemogamasinae Oudemans in northwest China. Chin J Hyg Insect＆Equip 2011; 17: 217–220. (in Chinese)

123. Liu ZJ, Zhang JJ. Small － sinica of Laelapidinae Berlese in Northwest China. Chin J Hyg Insect＆Equip 2011; 17: 277–280+282. (in Chinese)

124. Luo F, Liu ZJ, Zhang JJ. Parasitidae insects in northwest China. J Med Pest Control 2011; 27: 309–310. (in Chinese)

125. Xie LX, Yang MF, Huang R. A new species of the genus *Epidamaeus* (Acari, Oribatida, Damaeidae) from China. ZooKeys 2011; 19. (in Chinese)

126. Zhan YZ. Community structure and host selection of chigger mites in 19 counties. Master thesis: Dalli College; 2011. (in Chinese)

127. Zhan YZ, Guo XG, Zuo XH, Wang QH, Wu D. Distribution of *Ascoschoengastia indica* in 19 counties of Yunnan province. Chin J Victor Biol & Control 2011; 22: 521–524. (in Chinese)

128. Gao XP, Bai XL, Ma LM. Investigations on mesostigmatic mites from Ningxia, China (Acari) (3). Bull Dis Control Prev 2012; 27: 21–23. (in Chinese)

129. Heng QJ, Feng SQ, Liu N, He YM, Li H, Zhu B, et al. Species and geographical distribution of fleas and gamasid mites on the rat-shape animals in Chongqing city. Chin J Hyg Insect ＆ Equip 2012; 18: 413–415. (in Chinese)

130. Lin JZ, Zhang YX, Ji J, Chen X, Ma LM. Investigation of free living gamasid mites in China (V) (Acari: Mesostigmata). Wuyi Science Journal 2012; 28: 23–27. (in Chinese)

131. Qiao FG, Bai XL, Ma LM. Investigations on mesostigmatic mites from Ningxia, China (Acari) (4). Bull Dis Control Prev 2012; 27: 24–26+32. (in Chinese)

132. Lin SJ. Researches on parasitical condition of chigger mites on small mammals. Master thesis: Dali College; 2012. (in Chinese)

133. Sun XM. Researches on parasitical condition of chigger mites on some rodents. Master thesis: Dali College; 2012. (in Chinese)

134. Zhou SH, Deng YQ, Li SY, Wang LL. Supplementary records of Dermanyssoid mites (Acari：Parasitiformes) in Fujian province. Chin J Victor Biol & Control 2012; 23: 467–470. (in Chinese)

135. Bai XL, Gao XP, Wei H. Species records relation on Mesostigmatic mites and Insect related in Ningxia ( 1). J Med Pest Control 2013; 29: 1302–1305. (in Chinese)

136. Bai XL, Ma LM. Investigations on mesostigmatic mites from Ningxia and neighboring Provinces (Acari) (2). Bull Dis Control Prev 2013; 28: 13–16. (in Chinese)

137. Gao XP, Bai XL, Tian T. Investigations on mesostigmatic mites from Pingluo, Ningxia. Bull Dis Control Prev 2013; 28: 17–19. (in Chinese)

138. Guo B, Geng ML, Guo XG. Distribution and host selection of *Leptotrombidium yui* in some areas of Yunnan Province. Journal of Dali University 2013; 12: 20–25. (in Chinese)

139. Guo B, Guo XG, Geng ML, Qian TJ, Dong WG, Wang QH. Distribution and host selection of *Helenicula simena* in some areas of Yunnan Province, China. Chinese Journal of Zoonoses 2013; 29: 418–421. (in Chinese)

140. Guo XG, Speakman JR, Dong WG, Men XY, Qian TJ, Wu D, et al. Ectoparasitic insects and mites on Yunnan red-backed voles (*Eothenomys miletus*) from a localized area in southwest China. Parasitology Research 2013; 112: 0. (in Chinese)

141. Huang XQ, Yang YM, Shen LJ, Guo XG. Analysis of gamasid mites (Acari: Mesostigmata) associated with the Asian house rat, *Rattus tanezumi* (Rodentia: Muridae) in Yunnan Province, Southwest China. Parasitology Research 2013; 112. (in Chinese)

142. Lin JZ, Zhang YX, Ji J, Chen X, Sun L, Zhai TY, et al. Investigation of free living gamasid mites in China (VI) (Acari: Mesostigmata). Wuyi Science Journal 2013; 29: 144–155. (in Chinese)

143. Liu YX, Jia N, Xing YB, Suo JJ, Du MM, Jia N, et al. Consistency of the key genotypes of orientia tsutsugamushi in scrub typhus patients, rodents, and chiggers from a new endemic focus of northern China. Cell Biochemistry and Biophysics 2013; 67. (in Chinese)

144. Wang QH, Shi AM, Guo XG, Song WY, Zhao N, Dong WG. Investigation of chigger mites on small mammals in a flatland area of Menghan, Xishuangbanna, Yunnan Province. Chin J Parasitol Parasit Dis 2013; 31: 303–306. (in Chinese)

145. Wang X, Yang HQ, Li HL, Wei YW, Wu KM, Yang N, et al. Investigation on species of chigger mites in Qinghai Province. Chin J Ctrl Endem Dis 2013; 28: 423–425. (in Chinese)

146. Zhan YX, Guo XG, Speakman JR, Zuo XH, Wu D, Wang QH, et al. Abundances and host relationships of chigger mites in Yunnan Province, China. Medical and Veterinary Entomology 2013; 27 . (in Chinese)

147. Gao XP, Bai XL, Tian T. Investigations on mesostigmatic mites from Ningxia, China (Acari) (4). Bull Dis Control Prev 2014; 29: 18–21. (in Chinese)

148. Li HL, Wei YW, Li C. Cluster analysis of gamasid mites in Qinghai Province, China. Chinese Journal of Zoonoses 2014; 30: 67–73. (in Chinese)

149. Li YJ, Liang HJ, Xing TY. Meteorological factors and risk of scrub typhus in Guangzhou, southern China, 2006–2012. BioMed Central 2014; 14. (in Chinese)

150. Lin JZ, Zhang YX, Ji J, Chen X, Sun L, Zhai TY, et al. Investigation of free living gamasid mites in China (VII) (Acari: Mesostigmata). Wuyi Science Journal 2014; 30: 41–57. (in Chinese)

151. Ma HR, Yan Y, Bai XL, Zhang T, Tian T, Guo FQ. Investigations on mesostigmatic mites from Jingyuan, Ningxia, China (Acari) (1). Bull Dis Control Prev 2014; 29: 14–18. (in Chinese)

152. Zhu QR. Study on the ecology of surface chigger mites of three species of house mice, including *Rattus flavescens*, in Yunnan province. Master thesis: Dali College; 2014. (in Chinese)

153. Yang Y, Cai ZM, Bai XL, Ma LM. Investigations on mesostigmatic mites from Ningxia, China (Acari) (5). Bull Dis Control Prev 2014; 29: 15–17. (in Chinese)

154. Liu D. Review of *Oribotritia* (Acari, Oribatida, Oribotritiidae) with a world checklist and description of a new species from China. Zootaxa 2015; 4007. (in Chinese)

155. Lin JZ, Dai WA, Lv Z, Yang J, Zhang YX, Chen X, et al. Investigation of free living gamasid mites in China (VIII) (Acari: Mesostigmata). Wuyi Science Journal 2015; 31: 43–54. (in Chinese)

156. MA M, Li SC, Fan QH. Mites and ticks (Acari) in Shanxi Province, China: an annotated checklist. Zootaxa 2015; 4006. (in Chinese)

157. Peng PY, Guo XG, Song WY, Hou P, Zou YJ, Fan R, et al. Analysis of ectoparasites (chigger mites, gamasid mites, fleas and sucking lice) of the Yunnan red-backed vole (*Eothenomys miletus*) sampled throughout its range in southwest China. Medical and veterinary entomology 2015; 29. (in Chinese)

158. Peng PY, Guo XG, Ren TG, Song WY. Faunal analysis of chigger mites (Acari: Prostigmata) on small mammals in Yunnan province, southwest China. Parasitology Research 2015; 114. (in Chinese)

159. Yang PB, Song WY, She HY, Wu XS, Jiang P, Zou YJ, et al. Diversity of ectoparasites on *Eothenomys miletus* in mountainous areas of southwest Sichuan. Sichuan Journal of Zoology 2015; 34: 239–244. (in Chinese)

160. Lin JZ, Bai XL, Zhang YX, Chen X, Sun L, Zhai TY, et al. Redescription and distribution of *Hypoaspis kargi Costa* in China (Acari: Mesostigmata: Laelapidae). Wuyi Science Journal 2016; 32: 32–34. (in Chinese)

161. Lin JZ, Zhang YX, Ji J, Chen X, Ma LM. Investigation of free living gamasid mites in China (IⅩ) (Acari: Mesostigmata). Wuyi Science Journal 2016; 32: 1–13. (in Chinese)

162. Cao M, Che L, Zhang JH, Hu JL, Feng YJ. Determination of scrub typhus suggests a new epidemic focus in the Anhui Province of China. Scientific reports 2016; 6:

163. Peng PY, Guo XG, Song WY, Hou P, Zou YJ, Fan R, et al. Ectoparasitic chigger mites on large oriental vole (*Eothenomys miletus*) across southwest, China. Parasitology research 2016; 115 .

164. Peng PY, Guo XG, Ren TG, Dong WG, Song WY. An updated distribution and hosts: trombiculid mites (Acari: Trombidiformes) associated with small mammals in Yunnan Province, southwest China. Parasitology Research 2016; 115.

165. Peng PY, Guo XG, Ren TG, Song WY, Dong WG, Fan R. Species diversity of ectoparasitic chigger mites (Acari: Prostigmata) on small mammals in Yunnan Province, China. Parasitology Research 2016; 115.

166. Zhang XX, Meng QL, Qiao J. Morphology identification and comparison of cox1 gene of *Dermanyssus gallinae* in Shihezi, Xinjiang. Journal of Shihezi University：Natural Science 2016; 34: 30–35. (in Chinese)

167. Chai Q, Ning T, Li CP. *Laelaps echidninus* found on skin of *Apodemus agrarius* in Wuhu area. Chin J Schisto Control 2017; 29: 340–341. (in Chinese)

168. He YL, Yang HY, Yu CX, Zhang X, Yi QH, Ma ZL, et al. Study on foci of tsutsugamushi epidemic area in Taizhou city, 2013-2014. Chinese Journal of Preventive Medicine 2017; 51: 252–256. (in Chinese)

169. Huang Y, Zhao L, Zhang Z, Liu M, Xue Z, Ma D, et al. Chigger Mite (Acari: Trombiculidae) survey of rodents in Shandong Province, Northern China. The Korean Journal of Parasitology 2017; 55. (in Chinese)

170. Guo ZN, Lin ZM, Wang JX. Survey of chigger mites and their hosts in Xiamen City. Chin J Hyg Insect ＆ Equip 2017; 23: 131–133. (in Chinese)

171. Jiang WL, Guo XG, Song WY, Peng PY, Ren TG, Qian TJ, et al. A further study on the distribution of *Leptotrombidium rubellum* in Yunnan province. Journal of Pathogen Biology 2017; 12: 979–982+993.

172. Tang TY, Tang Y, Tang J. Investigation on rodents, bats and parasite in Dazhu County of Sichuan Province. J Med Pest Control 2017; 33: 93–94+97. (in Chinese)

173. Lin JZ, Zhang YX, Chen X, Ji J, Ma LM. Investigation of free living gamasid mites in China (Ⅹ) (Acari: Mesostigmata). Wuyi Science Journal 2017; 33: 1–12. (in Chinese)

174. Lu MG, Jiang QL, Gong ZY, Ni QX, Ma LM. A list of Gamasid mites (Acari: Gamasina) in Zhejiang province. Chin J Vector Biol & Control 2017; 28: 269–273. (in Chinese)

175. Su J, Li ML, Wang RL. Epidemic and diagnosis characteristic of Akamushi disease in Henan in 2016. Henan J Prev Med 2017; 28: 481–483. (in Chinese)

176. Huang YT, Zhao Li, Zhang ZT, Liu MM, Xue ZF, Ma DQ, et al. Detection of a novel Rickettsia from *Leptotrombidium scutellare* Mites (Acari: Trombiculidae) From Shandong of China. Journal of medical entomology 2017; 54 . (in Chinese)

177. Chang HQ, Wang ZY, Liu H. Four new feather mite species of the genus *Anhemialges* *Gaud*, 1958 (Astigmata: Analgidae) from China. Zootaxa 2018; 4531 . (in Chinese)

178. Lin JZ, Yang J, Zhang YX, Dai WA, Chen X, Xiang D, et al. Investigation of free living gamasid mites in China (ⅩI) (Acari: Mesostigmata). Wuyi Science Journal 2018; 34: 16–32. (in Chinese)

179. Zhang WZ, Ma TZ, Wu D. Ecological investigation and analysis of small mammals and their ectoparasites in Wutai Mountain scenic area, Beijing. Chin J Vector Biol & Control 2018; 29: 27–31. (in Chinese)

180. Zheng LH, Chen J. Taxonomic study on the genus *Fissicepheus* (Acari: Oribatida: Otocepheidae) from China. Zootaxa 2018; 4410: 539–550. (in Chinese)

181. Lin JZ, Dai WA, Zhang YX, Lin S, Chen X, Sun L. Investigation of free living gamasid mites in China (ⅩII) ( Acari: Mesostigmata). Wuyi Science Journal 2019; 35: 127–134. (in Chinese)

182. Zheng QF, Liang WQ, Ren GR, Yang MF. A new species and two newly recorded species of the subgenus *Pergalumna* (*Pergalumna*) (Acari, Oribatida, Galumnidae) from China. Zootaxa 2019; 4647. (in Chinese)

183. Wang YJ, Yan DM, Li GC. Epidemiological characteristics of scrub typhus in Pinggu district of Beijing, China, in 2008-2018. Chin J Vector Biol & Control 2019; 30: 244–247. (in Chinese)

184. Liang WQ, Yang MF, Ren GR, Zheng QF. New species and new records of the subgenus *Galumnella* (*Galumnella*) (Acari: Oribatida: Galumnellidae) from China. Zootaxa 2019; 4647 . (in Chinese)

185. Yun X, Li X, Zhang FP. Two new records of the Family Celaenopsidae (Acari: Mesostigmata) from China, with description of a new species. Zootaxa 2019; 4604 . (in Chinese)

186. Li TG, Yang ZC, Dong ZQ, Wang M. Analysis of rodent surveillance at Raohe port in Heilongjiang province. Chinese Frontier Health Quarantine 2020; 43: 254–256+272. (in Chinese)

187. Liu D. Contribution to the knowledge of the ptyctimous mite genus *Austrophthiracarus* (Acari, Oribatida, Steganacaridae) with descriptions of two new species from China. Zootaxa 2020; 4786 . (in Chinese)

188. teshu G. Epidemiology of tsutsugamushi disease and its relationship with meteorological factors in Xiamen city, China. PLoS neglected tropical diseases 2020; 14: 0.

189. Ding F, Jiang WL, Guo XG, Fan R, Mao KY, Zhao CF, et al. A preliminary report on Walchia microplate in Yunnan Province. Sichuan Journal of Zoology 2020; 39: 555–562. (in Chinese)

190. Pan ZW, Deng GF. China Economic Entomology, Book 17 Acarina, Gamasidae. Beijing: Science Press; 1980. (in Chinese)

191. Cao WC, Fang LQ, Wang JL. Atlas of Epidemiology of Natural Epidemic Diseases in China. Beijing: Science Press.; 2019. (in Chinese)

192. Zhang Y, Li FQ, Shen JZ. Study on natural infection of EHFV in *Letotrombidium* (*L*.) *scutellare*. Virologica Sinica 1995; 1: 94–96. (in Chinese)

193. ZHU J, Zhang Y, Tang JQ, Li XF, Guo HB, Pan XZ, et al. Nested reverse transcription-polymerase chain reaction for detection of hemorrhagic fever with renal syndrome virus R. Chinese Journal of Zoonoses 1998; 5: 2–5. (in Chinese)

194. Wang SQ, Liang LS, Guo KY, Yu ES. Investigation on the foci of scrub typhus in winter. Fujian Journal of Medicine 1983; 6: 23–24. (in Chinese)

195. Zhou PL, Liu LQ, Zhu LD, Mai ZQ, Cai HZ. Investigation on rodent mite and tsutsugamushi in southern mountainous area of Hunan province (abstract). Medical data of Guanghou 1983; 1: 95–97. (in Chinese)

196. Wang SQ, Guo YY. Vertical distribution of chigger mites and their hosts. Fujian Journal of Medicine 1985; 6: 26–27. (in Chinese)

197. Zhang Y, Li XF, Zhu J, Tang JQ, Li YX, Wu GH, et al. Investigation on the natural infection of gamasid mite with epidemic hemorrhagic fever virus. Jiangsu medicine 1985; 6: 2–4. (in Chinese)

198. Liu GD, Liu GP, Quan LH, Lou D, Zhang XW. Investigation report of rickettsial disease in Hunchun area of Jilin province. People's Military Surgeon 1987; 12: 25. (in Chinese)

199. Wei JJ, Tong GZ, Shi SF. Study on *Leptotrombidium Insularae*-a new vector of tsutsugamushi diseases in China. PLA Medical Journal 1987; 6: 415–418. (in Chinese)

200. Zhang Y, Zhao XZ, Zhang BG. Investigation on the host animal of epidemic hemorrhagic fever in shrew musk. Chinese Journal of Public Health Management 1987; 4: 209–210. (in Chinese)

201. Liu SL, Wang LJ. Epidemiological investigation of tsutsugamushi in fei county. Shandong medicine 1988; 11: 9. (in Chinese)

202. Su DM, Jiang RJ, Wang ZD, Wang BX, Ji GD, Yang LB, et al. Investigation report on the epidemic of tsutsugamushi in Dongtai city. Jiangsu medicine 1989; 5: 239–241. (in Chinese)

203. Wang ZC, Yuan YH, Li XH, Li ZH, Li SQ. A strain of Yersinia pestis was isolated from the body of the hamster Acari erectus for the first time. Chinese Journal of Zoonoses 1989; 5: 54. (in Chinese)

204. Fang YM, You ZQ, Xun L, Lin L, Miu FX, Bi JQ, et al. A report of 23 cases of scrub typhus in Jinhu County. Jiangsu Medical Journal 1990; 9: 519. (in Chinese)

205. Qian JY, Zhang Y, Tao KH, Zhu J, Wu GH. Investigation of the relationship between chigger mites and hemorrhagic fever with renal syndrome. Chinese Journal of Vector Biology and Control 2000; 3: 166–168. (in Chinese)

206. Wang JL, Yang YF, Yao YC. Investigations on outbresk of tsutsugamushi disease in Four Suceessive Years in a Village. Journal of Linyi Medical College 1990; 3: 215–217. (in Chinese)

207. Yang ZQ, Yu XM, Jia JQ. Rapid Dectection of Rotavirus in Stools by Double-antibody Sandwish ELISA (One-step Method). Chinese Journal of Zoonoses 1990; 6: 27. (in Chinese)

208. Yu XM, Yang ZQ, Li P, Meng XR. Serological investigation of rickettsial infection in some population in Jinan and Junan county. Journal of Preventive Medicine of Chinese People's Liberation Army 1990; 4: 399–400. (in Chinese)

209. Liu GD, Han WG, Li L, Cao JW, Sun JB, Han ZY, et al. Investigation on natural infection of tsutsugamushi among healthy people in Dachangshan Island. People's Military Surgeon 1991; 6: 15+22. (in Chinese)

210. Liu GD, Han WG, Li L, Guo YX, Wang JQ, Cai SR, et al. Investigation on the natural infection of tsutsugamushi in some healthy people in the ocean island of Liaodong province. Chinese Journal of Zoonoses 1991; 3: 60–61. (in Chinese)

211. Zhou PS. Survey of the natural foci of the Tsutsugamushi Disease in the Southwest Region of Guangxi. Chinese Journal of Vector Biology and Control 1991; 3: 206–209. (in Chinese)

212. Wang JL, Yang YF, Yao YC. Type and Characteristics of epidemic focus of Tsutsugamushi disease in Yimeng Mountain Area. Journal of Linyi Medical College 1991; 3: 235–237. (in Chinese)

213. Cao XR, Wu JJ, Zhang YG, Wang YN, Yang CM, Xue ZP, et al. Recent studies on scrub typhus and *Rickettsia tsutsugamushi* in Shandong Province--China. Eur J Epidemiol 1991; 7: 304–306. (in Chinese)

214. Zhao Y, Zhang LN, Wang Y, Ma LM. Study of gamasid mites as the vector reservoir of Epidemic Hemorrhagic Fever (EHF). Chinese Journal of Vector Biology and Control 1991; 5: 316–318. (in Chinese)

215. Feng XG, Yuan QH, Chen YM, Zhang HL. Antigen typing and serotyping of *Rickettsia tsutsugamushi* epidemic strains in Huaping County, Yunnan Province. Yunnan medicine 1992; 6: 368–369. (in Chinese)

216. Liu GD, Li L, Han WG, Hao WH, Zhang ZC, Guo YX, et al. Investigation on natural infection of tsutsugamushi in some healthy people in Zhangzidao. Journal of Preventive Medicine of Chinese People's Liberation Army 1992; 2: 43–45. (in Chinese)

217. Zhang Y, Li XF, Zhu J, Tang JQ, Wu GH, Zhang LL. Preliminary studies on prolif eration of hemorrhagic fever with renal syndrom virus in *Leptotrombidium* (*L*.) *Scutellare*. Chinese J Exp Clin Virol 1997; 3: 63–66. (in Chinese)

218. Piao CG, Cui ZS, Li M, Cui Zg, Yu XJ, Bi DZ, et al. Serological confirmation of *Tsutsugamushi rickettsia* infection in Chunhua town of Hunchun city. Chinese Journal of Zoonoses 1992; 3: 34–35. (in Chinese)

219. Yang ZQ, Yu XM, Li P. Seroepidemiological investigation of tsutsugamushi in some areas of Shandong Province. Chinese Journal of Public Health 1992; 11: 522. (in Chinese)

220. Zhang ZL, Luo YQ, Zhang Y. Investigation of natural Ctesia infection in Tianjin population. Chinese Journal of Public Health 1992; 6: 253–254. (in Chinese)

221. Lu ZX, Hu LM, Cai ZL, Jin XT, Sun HL, Zheng XH, et al. Epidemiological investigation of tsutsugamushi natural foci in Hunchun city. Public Health in China 1993; 5: 207–208. (in Chinese)

222. Wu YS, Zhang HL, Liu GZ, Fang YM. Serum antibody and its typing of tsutsugamushi in some areas of Jiangsu province. Chinese Journal of Zoonoses 1993; 5: 41–42. (in Chinese)

223. Guo HB, Wu GH, Xu MH, Liu Y, Yu MM, Shen JZ, et al. Investigation on natural foci of tsutsugamushi in autumn and winter. Chinese Journal of Epidemiology 1994; 1: 27–30. (in Chinese)

224. Lei YM, Feng XG, Chen YM, Zhang HL. Serum classification of *Rickettsia tsutsugamushi* in Yunnan Province. Chinese Journal of Zoonoses 1994; 2: 62–63. (in Chinese)

225. Lu ZX, Hu LM, Cai ZL, Jin XT, Sun HL, Zheng XH, et al. Confirmation of natural foci of tsutsugamushi in eastern Heilongjiang province. Shenyang army medicine 1994; 6: 540–541. (in Chinese)

226. Lu ZX, Hu LM, Cai ZL, Jin XT, Li YL. Epidemiological investigation of tsutsugamushi disease in natural foci of Mishan city. Chinese Journal of Public Health 1994; 5: 200–201. (in Chinese)

227. Lu ZX, Hu LM, Cai ZL, Jin XT, Zhao ZL, Chen TC, et al. Hunchun: a newly discovered natural foci of scrub typhus disease. Chinese Journal of Epidemiology 1994; 1: 31–33. (in Chinese)

228. Lu ZX, Hu LM, Cai ZL, Jin XT, Zhao YG, Zhu ZH, et al. Epidemiological investigation of tsutsugamushi disease in natural foci of Liaoning province. Chinese Journal of Public Health 1994; 12: 535–536. (in Chinese)

229. Wu AG, Guo XG. Research on the species constitution and interspecific association of gamasid mite in Gengma, Yunnan. Journal of Dali Medical College 1994; 2: 36–38+60. (in Chinese)

230. Liu FM, Sun CH, Liu XP, Wang ZL, Zhen TM, Hu YX. The experimental studies on the Poison Effects of Different Cloths Bed Nets impregnated with different dosages of Alphamethrin. Chinese Journal of Vector Biology and Control 1994; 2: 103. (in Chinese)

231. Lu ZX, Hu LM, Cai ZL, Jin XT, Chen TC, Li ZY. Confirmation of tsutsugamushi natural foci in northeast China. Medical Information of Chinese PLA 1995; 1: 40. (in Chinese)

232. Wang XJ, Wang QZ, Li Z. Study on the pathogen of tsutsugamushi in Shandong province. Chinese Journal of Zoonoses 1995; 5: 47–48. (in Chinese)

233. Yu HC, Sun W, Liu Z, Tan X, Wang J, Hu S, et al. Experimental study on infection of Epidemic Haemorrhagic Fever Virus in *Leptotrombidium palpale*. Chinese Journal of Zoonoses 1995; 3: 10–11. (in Chinese)

234. Hu LM, Lu ZX, Cai ZL, Jin XT, Zhao ZL. Serological investigation of scrub typhus in some areas of northeast China. Chinese Journal of Epidemiology 1996; 1: 32. (in Chinese)

235. Lu ZX, Hu LM, Cai ZL, Shen BJ, Liu GP, Lu H, et al. Basic characteristics of natural foci of scrub typhus in northeast China. Chinese Journal of Public Health 1996; 8: 349–350. (in Chinese)

236. Lu ZX, Hu LM, Jin XT, Lu H. A *Rickettsia tsutsugamushi* strain was isolated from the blood of the patient. Chinese Journal of Public Health 1996; 2: 86. (in Chinese)

237. Wang JL, Li P, Duan AX. A Research Report of *Shashitsu* in Mengyin County. Journal of Linyi Medical College 1996; 2: 123–125. (in Chinese)

238. Zhou PS, Cai HZ, Shi JZ. Study on comprehensive prevention and control of tsutsugamushi disease in the Wuzhishan area of Hainan Province. Public Health in China 1996; 7: 304. (in Chinese)

239. Chen LB, Tian FJ, Sun T, Wang XJ, Du XG, Ma LZ, et al. The epidemic of scrub typhus was first found in Rencheng District of Jining City. Chinese Journal of Zoonoses 1997; 5: 70–71. (in Chinese)

240. Chi YD, Chen DR. Studies on Serotyping of *Rickettsia tsutsugamushi* in Yunnan. Chinese Journal of Zoonoses 1997; 1: 13–16. (in Chinese)

241. Lin BH, Sun XJ, Zhan ZN, Lin YZ, Li WG. Serological and etiological investigation of rickettsial disease in Hainan Island. Chinese Journal of Microbiology and Immunology 1997; 5: 55. (in Chinese)

242. Liu YX, Wu QY, Sun HL, Yang ZQ, Meng XR, Su M, et al. Studies on clinical epidemiology and etiology of scrub typhus of autumn-winier type in Fei county, Shandong province. Journal of Preventive Medicine of Chinese People's Liberation Army 1997; 4: 25–28. (in Chinese)

243. Liu YX, Wu QY, Su M, Zhang XL. Investigation of host animals and chigger mites in autumn and winter tsutsugamushi foci in Fei county, Shandong province. Journal of Medical Pest Control 1997; 3: 145–148. (in Chinese)

244. Lu ZX, Hu LM, Cai ZL, Lu H, Jin XT, Zhao ZL, et al. Host investigation of natural foci of scrub typhus in northeast China. Chinese Journal of Vector Biology and Control 1997; 3: 222–223. (in Chinese)

245. Zhuge HX, Meng YC, Lan MY. Natural infection in and transmission of EHF virus through biting by gamasid mites, *Tricholaelaps myonyssognathus* and *Eulaelaps stabularis*. Chinese Journal of Public Health 1987; 6: 335–336. (in Chinese)

246. Zhang Y, Wu GH. Study on detection of hemorrhagic fever with renal syndrome virus structural protein in chigger mite. Chinese Journal of Zoonoses 1998; 1: 7–9. (in Chinese)

247. Sun HL, Yang ZQ, Liu YX. Investigation on natural infection of *Rickettsia tsutsugamushi* in some areas of Shandong province. Preventive Medicine Tribune 1997; 2: 117. (in Chinese)

248. Wu JP, Cai N, Lu ZX. Investigation of *Rickettsia tsutsugamushi* antibody in Hunchun area of Jilin province. Shenyang army medicine 1997; 5: 426–427. (in Chinese)

249. Zheng XZ, Huo Q, Jiang ZL. Epidemiological investigation of tsutsugamushi in southern Shanxi. Journal of Preventive Medicine of Chinese People's Liberation Army 1997; 3: 44–45. (in Chinese)

250. Zhou XR, Liu SK, Ji BX, Dou J, Dang RL, Wang TX. Serological Survey on *Rickettsia tsutsugamushi* Infection Among INhabitants and Sheep in Ali Prefecture, Tibet. Endemic Diseases Bulletin 1997; 2: 76–77. (in Chinese)

251. Gao LF, Wu XL, Hu HM, Qian FB, Luo QM, Yuan JY, et al. Studies on serological typing of *Rickettsia tsutsugamushi* in Binchuan County of Yunnan. Chinese Journal of Vector Biology and Control 1998; 4: 53–54. (in Chinese)

252. Huang XD, Cheng P, Zhao YQ, Wen JL, Zhao JX, Liu Hm, et al. Epidemiological investigation of tsutsugamushi in Nanri Island. Chinese Journal of Zoonoses 1998; 4: 77–78+72. (in Chinese)

253. Liu YX, Wu QY, Yang ZQ, Peng ZL, Miao ZS, Cong LZ. Dynamic observation of *Rickettsia tsutsugamushi* antibody in rat serum in Fei county, Shandong province. Chinese Journal of Public Health 1998; 2: 61. (in Chinese)

254. She JJ, Zhang Y, Huang CA, Yu MM, Jiang KJ, Wu GH. Preliminary Study on *Leptotrombidium* (*L*.) subpalpale as Spreading Medium of HFRS. Chinese Journal of Vector Biology and Control 1998; 1: 55–58. (in Chinese)

255. Wei CZ, Wang YF. Investigation on tsutsugamushi foci in forest area of Yongan city. Strait Journal of Preventive Medicine 1998; 3: 26–27. (in Chinese)

256. Zhuge HX, Meng YC, Wu JW, Zhu ZY, Liang WF, Yao PP. Studies on the experimental transmission of rattus-borne hantavirus by *Ornithonyssus bacoti*. Chinese Journal of Parasitology and Parasitic Diseases 1998; 6: 47–50. (in Chinese)

257. Feng XG, Yuan QH, Zi DY, Zhang HL, Chen YM. Serological Type of Patients with *Rickettsia tsutsugamushi* in Dali prefecture of Yunnan Province. Endemic Diseases Bulletin 1999; 4: 18–19. (in Chinese)

258. Liu YX, Wu QY, Yang ZQ, Peng ZL, Miao ZS, Qin DT, et al. Investigation on Epidemiology and Affecting Factors of Autumn-winter Type Scrub Typhus in Fei County, Shandong Province. Disease Surveillance 1999; 2: 11–15. (in Chinese)

259. Xu J, Chen LF, Lu ZX, Sun CQ, Liu ZW, Sun RF, et al. Serological investigation of *Rickettsia tsutsugamushi* infection in some population in Heilongjiang province. Chinese Journal of Public Health 1999; 12: 61–62. (in Chinese)

260. Yuan QH, Feng XG, Mi ZQ, Zhang HL, Zi DY, Chen YM. Investigation on the outbreak of tsutsugamushi in Binchuan County, Yunnan Province. Chinese Journal of Zoonoses 1999; 1: 78–79. (in Chinese)

261. Zhang RM, Wang MY. Investigation report on an outbreak of forest encephalitis. Disease surveillance 1999; 4: 149. (in Chinese)

262. Zhang Y, Zhu J, Tao KH, Wu GH, Guo HB, Wang JJ, et al. Detection of Hemorrhagic Fever with Renal Syndrome Virus Structural Protein and Gene in Gamasid mite and Chigger Mite. Chinese Journal of Vector Biology and Control 1999; 4: 58–60. (in Chinese)

263. Chen L, Yan YS, He S. Study on vector of *Orientia tsutsugamushi* and specimens of blood clots and spleen collected from artificial infected mice and field rodents by nested polymerase chain reaction. Chinese Journal of Vector Biology and Control 2000; 3: 216–219. (in Chinese)

264. Chen XR, Yu Q, Zhang YG. Investigation of scrub typhus in Shanxi Province and study on its etiology. Military Medical Sciences 2000; 4: 275–277+281. (in Chinese)

265. Jiang PL, Wang SS, Huang JL, Peng JF, Zeng NH, Liu JH, et al. The confirmation of the epidemic area of tsutsugamushi disease on Nanao county in chi. Chinese Journal of Zoonoses 2000; 6: 24–27. (in Chinese)

266. Luo LP, Guo XG, Qian TJ. Investigation of antibody to tsutsugamushi in wild rodents in some areas of three provinces in northeast China. Chinese Journal of Zoonoses 2000; 5: 65. (in Chinese)

267. Yu SR. Research progress of Q fever in China. Chinese Journal of Epidemiology 2000; 6: 56–59. (in Chinese)

268. Xue JQ, Ming J, Yang HL. Investigation on Tsutsugamushi Disease Prevalence in Feicheng, Shandong Province. Disease Surveillance 2000; 10: 367–369. (in Chinese)

269. Yuan GL, Zheng ZJ, Chen WJ, Li XY, Li FP. Epidemiological investigation of tsutsugamushi disease in Ningde region from 1997 to 1998. Chinese Journal of Zoonoses 2000; 6: 109. (in Chinese)

270. Zeng NH, Wang ZB, Huang JL, Peng GF, Wang SS, Jiang PL, et al. Investigation and analysis of tsutsugamushi foci in Shantou coastal island. Journal of Preventive Medicine of Chinese People's Liberation Army 2000; 3: 196–197. (in Chinese)

271. Zhang Y, Zhu J, Deng XZ, Wu GH, Zhang JJ, Zhou YP. Study on detection of Hemorrhagic Fever with Renal Syndrome Virus gene in cultured cells of Gamasid Mites and Chigger Mites. CHI PUBLIC HEALTH 2000; 12: 24–25. (in Chinese)

272. Chen SL, Li CM, Shi J, Zhou LQ, Kong LY, Xu JH, et al. Epidemiological study on incidence characteristic of Tsutsugamushi disease in certain village of Hebei Province. Chinese Journal of Vector Biology and Control 2001; 2: 125–126. (in Chinese)

273. Chen Sl, Zhang YL, Gou YX, Zhou LQ, Li CM, Li GY, et al. Seroepidemiological study of Tsutsugamushi Disease in Hebei Province. Chinese Journal of Vector Biology and Control 2001; 2: 122–124. (in Chinese)

274. Feng XG, Chen YM, Zi DY. A Survey and Research of Scurs Typhus in Jinshajiang River basin of Northern Part of Yunnan. Chinese Journal of Vector Biology and Control 2001; 2: 120–121. (in Chinese)

275. Feng XG, Lei YM, Chen YM, Zhang HL. Serum classification of tsutsugamushi disease patients in Yingjiang County, Yunnan Province. Chinese Journal of Vector Biology and Control 2001; 2: 133. (in Chinese)

276. Huang JL, Wang SS, Zhu SF, Jiang PL, Zeng NH, Wang ZB. Isolation and Identification of *Rickettsia Tsutsugamushi* from NanAo and Nan Peng Lie Island. Chinese Journal of Public Health 2001; 4: 25–26. (in Chinese)

277. Jiang PL, Wang SS, Huang JL, Peng JF, Zeng NH, Liu JH, et al. Characteristics of tsutsugamushi foci in Nanao county, China. Chinese Journal of Preventive Medicine 2001; 4: 32. (in Chinese)

278. Tang TK, Zhan DC, Lu ZR, Fan CH, Peng XJ. Epidemiological investigation of tsutsugamushi in tropical coral islands. CHI PUBLIC HEALTH 2001; 10: 59–60. (in Chinese)

279. Yuan QH, Yang WH, Mi ZQ, Huang WL, Zhang HL. Antibody investigation of tsutsugamushi disease in fever patients in Dali area of Yunnan province. Endemic Diseases Bulletin 2001; 2: 31–32. (in Chinese)

280. Zhang Y, Wu GH, Deng XZ, Wang ZC, Cao GW. Research progress of gamasid mites and chigger mites as vectors of hemorrhagic fever with renal syndrome. Chinese Journal of Zoonoses 2001; 3: 87–88+96. (in Chinese)

281. Zhang Y, Hu YL, Zhao XZ, Wu GH, Jiang KJ. Experimental study on the roles of gasmid mite and chigger mite in the transmission of hemorrhagic fever with renal syndrome virus. Chinese Journal of Epidemiology 2001; 5: 38–40. (in Chinese)

282. Wu GH. Epidemiological study of tsutsugamushi in east China. Jiangsu Journal of Preventive Medicine 2002; 4: 81–83. (in Chinese)

283. Hu FQ, Hu LM. Serological investigation of four kinds of *Rickettsiasis* in Kuandian area, Liaoning province. Chinese Journal of Zoonoses 2002; 6: 111–118. (in Chinese)

284. Zu WG, Li CM, Chen SL, Guo YX, Kong LY, SHI ZL. Investigation of tsutsugamushi foci in Taicang mountain area of Hebei Province. Chinese Journal of Parasitic Diseases Control 2002; 4: 73–74. (in Chinese)

285. Cao M, Guo Hb, Tang J. Detection of antibody against *Orientia tsutsugamushi* in serum of garrison and resident population on an island. Journal of Preventive Medicine of Chinese People's Liberation Army 2003; 1: 65. (in Chinese)

286. Pan RS, Zhang Y, Tan JM, Huang Q, Li XS, Mo GY. Baseline survey on taxonomy and population fluctuation of murine animals and their ectoparasites; forecast research on the related diseases of natural focus in Maoming port. Chinese Journal of Vector Biology and Control 2003; 5: 364–367. (in Chinese)

287. Li HB, Wei AM, Zhang ZQ, Wang LQ, Zhao XL, Zheng J, et al. Investigation of antibody against tsutsugamushi in human serum in some areas of three provinces in northeast China. Shenyang army medicine 2003; 3: 219. (in Chinese)

288. Yuan GL, Chen WJ, Li XY, Li FP. Geographical epidemiology of tsutsugamushi in Ningde city. Chinese Journal of Vector Biology and Control 2003; 5: 372–374. (in Chinese)

289. Zhang Y, Li XS, Huang Q, Pan JM, Zhang XM, Liu YH. The surveillance disease of natural focus and analysis of its epidemic factors at Maoming Port Area. Science of Travel Medicine 2004; 4: 16–18. (in Chinese)

290. Cao M, Guo Hb, Yu XM, Yang WF, Wang BR, Zhang Y, et al. Field detection of samples of scrub typhus by D micro assay and preventive strategies in spring at Pingtan Island in Fujiang province. Medical Journal of National Defending Forces in Southwest China 2005; 5: 467–470. (in Chinese)

291. Wu ZW. Epidemiological investigation of rickettsia in Henan province. Master thesis: Shandong University; 2005. (in Chinese)

292. Xu BL, Chen HM, Zhu Q, Zhang J, Xia SL, Li ML, et al. Epidemiological investigation of the first tsutsugamushi outbreak in Henan province. Henan Journal of Preventive Medicine 2006; 3: 129–131. (in Chinese)

293. Zhang CH, Liu JR, Ye YL, Tang BC. Investigation on tsutsugamushi foci in a reservoir area in eastern Fujian. Chinese Journal of Vector Biology and Control 2006; 5: 352. (in Chinese)

294. Zhang Q, Liu XY, Gao Y, Zhao ZT, Zhang JL, Yang ZQ, et al. Study on the molecular epidemiology regarding the natural infection of *Orientia tsutsugamushi* in 4 species of dominant chiggers collected in various seasons from the foci of Shandong province. Chinese Journal of Epidemiology 2006; 7: 600–603. (in Chinese)

295. Wang SS, Huang JL, Su JX, Xi YZ, Wang Y. Determination of tsutsugamushi foci in Wanshan archipelago of China. Chinese Journal of Zoonoses 2007; 8: 842–843. (in Chinese)

296. Xia SL, Shen XQ, Deng WB, Huang LL, Wang JL, Li LH, et al. The lab identification on tsutsugamushi disease for the first outbreak in Henan province. Chinese Journal of Vector Biology and Control 2007; 3: 230–233. (in Chinese)

297. Li MM, Wang SS, Li J, Liu JH, Su JX, Tang BR, et al. Seroepidemiological investigation on *Orientia tsutsugamushi* of the important South-Sea islands of Guangdong Province, China. Journal of Pathogen Biology 2008; 10: 743–744+753. (in Chinese)

298. Wang SS, Huang JL, Su JX, Peng GF, Wang Y, Li MM. Epidemiological study of tsutsugamushi disease in Naozhou Island of Guangdong Province. Journal of Tropical Medicine 2008; 1: 58–60. (in Chinese)

299. Wang SS, Huang JL, Su JX, Peng GF, Wang Y. Characteristics of tsutsugamushi foci in Leizhou peninsula, China. Chinese Journal of Vector Biology and Control 2008; 1: 70–72. (in Chinese)

300. Wu ZF, Deng HZ, Zhu FX, Wen JH, Liu SH. Investigation of tsutsugamushi disease in Ganzhou city, Jiangxi province. Journal of New Medicine 2008; 6: 390–391. (in Chinese)

301. Liu YX, Zhao ZT, Feng PT, Ma SB, Min JS, Qin DT, et al. Clinical manifestations and epidemic factors of autumn-winter type scrub typhus in children from northern new endemic area. Chinese journal of pediatrics 2008; 46 . (in Chinese)

302. Chen WH, Chen Q, Hua GR. Epidemiological investigation of Tsutsugamushi Disease in Huaiyuan County. Chinese Journal of General Practice 2009; 7: 297–298. (in Chinese)

303. Liu YX, Feng D, Suo JJ, Xing YB, Liu G, Liu HJ, et al. Clinical characteristics of the autumn-winter type scrub typhus cases in south of Shandong province, northern China. BioMed Central 2009; 9 . (in Chinese)

304. Liu YX, Jia N, Xing YB, Suo JJ, Liu G, Xiao HJ, et al. Characteristics of pediatric scrub typhus in a New Endemic Region of Northern China. The Pediatric Infectious Disease Journal 2009; 28: 2. (in Chinese)

305. Qian JY, Deng XZ, Zhang Y. Investigation of *Leptotrombidium scutellare*-hemorrhagic fever with renal syndrome vector. Chinese Journal of Vector Biology and Control 2009; 20: 583–584. (in Chinese)

306. Chai CL, Lu QY, Sun Jm. Seroepidemiological investigation of tick-borne diseases in human and domestic animals in Zhejiang Province. Chinese Journal of Epidemiology 2010; 10: 1144–1147. (in Chinese)

307. Chang K, Lee NY, Ko WC, Lin WR, Chen YH, Tsai JJ, et al. Seroepidemiologic investigation on rickettsiosis of humans and domestic animals in Yunnan province. Chinese Journal of Zoonoses 2010; 26: 189–192+197. (in Chinese)

308. Liu H, Cao MH, Zhang YG, Shi YL, Wang J, Zhang LJ. Seroepidemiological investigation on Tsutsugamushi Disease of people and livestock in different region of Anhui Province. Anhui Journal of Preventive Medicine 2010; 16: 12–13. (in Chinese)

309. Jiang RJ, Shen JJ, Zhang YZ, Zhang HJ, Guo HB, Zhang SY, et al. Epidemiological study of scrub typhus disease in Yancheng city during 2006 to 2010. Journal of Medical Pest Control 2011; 27: 1079–1081. (in Chinese)

310. lei YL, Chen XY, Liu FM, Shi GX, Mei JH, Wang XG, et al. Detection of *Rickettsiae tsutsugamushi* in rodents in Lishui city of Zhejiang Province. Disease Surveillance 2011; 26: 118–119. (in Chinese)

311. Ren LS, Dang RL, Liu XM, Gao JY, Ma DX. Investigation on animal natural infection of *Orientia tsutsugamushi* in some areas of Changji area in Xinjiang. Journal of Tropical Medicine 2011; 11: 1052–1053+1068. (in Chinese)

312. Dang RL, Ren LS, Li HL, Ma DX, Dong LN. Investigation on host animal and transmission media in epidemic focus of tsutsugamushi in northern areas of Xinjiang. Journal of Medical Pest Control 2012; 28: 1188–1190. (in Chinese)

313. Li J, Tan ZY, Li L. Cross-sectional survey on the prevalence of antibodies to several types of *Rickettsia* in human and livestock in Jiangsu province. Suzhou University Journal of Medical Science 2012; 32: 445–449+593. (in Chinese)

314. Liu D. Study of Composition of rodents and their ectoparasites and pathogens infection in the adjacent port area of Changbai mountain of China and Democratic People’s Repiblic of Korea. PhD thesis: Academy of Military Sciences of the People's Liberation Army; 2012. (in Chinese)

315. Ren LS, Dang RL, Ma DX, Gao JZ, Liu XM. Investigation on infection in mankind and animal of *Orientia tsutsugamushi* in some areas in Xinjiang， China. Journal of Tropical Medicine 2012; 12: 1249–1251+1257. (in Chinese)

316. Wang QK, Ge Hm, Li ZF, Shan YF, Cui L, Wang YP. Vector research of severe fever with thrombocytopenia syndrome virus in gamasid mites and chigger mites. Chinese Journal of Vector Biology and Control 2012; 23: 452–454. (in Chinese)

317. Li WB, Dou XF, Zhang LQ, Lv YN, Wang QY, Li XY, et al. Laboratory diagnosis and genotype identification of scrub typhus from Pinggu district, Beijing, 2008 and 2010. The American journal of tropical medicine and hygiene 2013; 89. (in Chinese)

318. Xiang F, Tian LL, Zhang LQ, Wang QY, Li XY, Lin H. Epidemiological characteristics of scrub typhus and estimation of the real case number in Beijing. Occupation and Health 2013; 29: 2765–2768. (in Chinese)

319. Ya HX, Wang JL. Serotyping and sequence analysis of *Oreintia tsutsugamushi* from patients with fever of unknown origin in Dali, Yunnan. Chinese Journal of Zoonoses 2013; 29: 899–903. (in Chinese)

320. Zhang JT, Zhang HJ, Guo TY, Chen CR, Yu Q. Seroepidemiological survey of Rickettsial disease in people and livestock in Lushi County, 2009. Henan Journal of Preventive Medicine 2013; 24: 418–421. (in Chinese)

321. Zhao W, Lao SJ, Wang CM, He HX. Molecular epidemiology of *Orientia tsutsugamushi* in chiggers and ticks from domestic rodents in Shandong, northern China. BioMed Central 2013; 6. (in Chinese)

322. Wei L, Wang XW, Wang CM, He HX. Study on ecology of small mammals and their ectoparasites in six areas of Sichuan province, China. Chinese Journal of Vector Biology and Control 2014; 25: 309–313. (in Chinese)

323. Shen AR, Luo QS, Yao LC, Shi M, Tian ZM. Analysis of epidemic situation of Tsutsugamushi disease in Longchuan county during 2009 － 2013. Journal of Medical Pest Control 2014; 30: 393–395. (in Chinese)

324. Chen YL, Yang YS, Jia LL, Dou XF, Liu YN, Geng LB, et al. Seroprevalence of scrub typhus in the north mountainous area of Beijing. Chinese Journal of Vector Biology and Control 2016; 27: 597–599. (in Chinese)

325. Jin HZ, Cao JH. Epidemiological characteristics of tsutsugamushi disease in Sheyang from 2006 to 2014. Journal of Medical Pest Control 2016; 32: 535–536+539. (in Chinese)

326. Liao H, Dai CW, Wang J. Distribution and infection status of host animals of tsutsugamushi in Nanshan District, Shenzhen City. Jiangsu Journal of Preventive Medicine 2016; 27: 496–497. (in Chinese)

327. Liu YN, Chen LJ, Dou XF, Sun YL, Zhang XC, Li XY, et al. Seroepidemiological investigation on scrub typhus in suburban districts of Beijing. Chinese Journal of Vector Biology and Control 2016; 27: 257–259. (in Chinese)

328. Cao M, Che L, Zhang JH, Dou XF, Sun YL, Zhang XC, et al. Determination of scrub typhus suggests a new epidemic focus in the Anhui Province of China. Scientific reports 2016; 6:(in Chinese)

329. Xu QY, Li HY, Li F, Yang GF, Zhang LJ. Serological investigation of vector born rickettsioses in agrarian children in Yili prefecture, Xinjiang Uygur Autonomous Region. Chinese Journal of Vector Biology and Control 2016; 27: 58–60. (in Chinese)

330. Zhang SG, Tian LL, Zhang LQ, Wan D. The prevalence of tsutsugamushi disease in Yanqing district of Beijing. Chinese Preventive Medicine 2016; 17: 532–534. (in Chinese)

331. Huang ZS, Guo HB, Liu KY. Detection of a novel *Rickettsia* from *Leptotrombidium scutellare* mites (Acari: Trombiculidae) from Shandong of China. J Med Entomol 2017; 54: 544–549.

332. Lai CH, Chang LL, Lin JN, Chen WF, Kuo LL, Lin SS, et al. High seroprevalence of Mycoplasma pneumoniae IgM in acute Q fever by enzyme-linked immunosorbent assay (ELISA). PLoS ONE 2017; 8: 0.

333. Li GC, Li XY, Liu J, Liu HJ, Liu JL, Li DM, et al. Investigation of chigger mites and infection of *Orientia tsutsugamushi* in small mammals in Qing′an county. Chinese Journal of Vector Biology and Control 2017; 28: 1–3+15.

334. Su J, Li ML, Wang RL. Epidemic and diagnosis characteristic of akamushi disease in Henan in 2016. Henan J Prev Med 2017; 28: 481–483. (in Chinese)

335. Xu HM, Bu D, Gema W, Hu SL, Zhang R, Duoji ZM, et al. Two cases of *Orientia tsutsugamushi* were confirmed by laboratory for the first time in Ningchi, Xizang. Tibetan medicine 2017; 38: 56–58. (in Chinese)

336. Zhan HY, Xu Z, He YL. Epidemiological characteristics and influencing factors of scrub typhus in Jingjiang City of Jiangsu Province from 2011 to 2015. Chinese Journal of Disease Control & Prevention 2017; 21: 1119–1122. (in Chinese)

337. Guo ZN, Chen M, Chen HF, Chen GW. Epidemiology and control strategy on Scrub typhus in Xiamen, China. Chinese Journal of Zoonoses 2018; 34: 1049–1055+1067. (in Chinese)

338. Ya HX, Dong WX, Wei PF, Zhou TH, Zhou JH, Zhang YZ, et al. Investigation on scrub typhus in Yongshan County, Yunnan Province, China. Chinese Journal of Zoonoses 2018; 34: 255–259. (in Chinese)

339. Zhang M, Zhao ZT, Yang HL, Zhang AH, Xu XQ, Meng XP, et al. Analysis of scrub typhus screening results from community hospitals. Acta Parasitological et Medica Entomological Sinical 2018; 25: 200–205.

340. Chang LT, Dao ZH, Liang CW. Characteristics of scrub typhus, murine typhus, and Q fever among elderly patients: Prolonged prothrombin time as a predictor for severity. Journal of Microbiology, Immunology and Infection 2019; 52.

341. Gao XP, Bai XL, Ma LM. Epidemiological analysis of three rodent-borne diseases in Chuxiong, 2006－2017. Modern Preventive Medicine 2019; 46: 1551–1554+1562. (in Chinese)

342. Li GC, Li XY, Chen CW, Liu JQ, Luo YX, Zhou TJ, et al. An investigation of natural focus of scrub typhus in Yongcheng, Henan province, China. Chinese Journal of Vector Biology and Control 2019; 30: 255–258. (in Chinese)

343. Li S, Wang RQ, Cai X, Jin WJ, Liu CC, Tang YQ, et al. Seroepidemiological survey and risk factors analysis of scrub typhus in Changping district of Beijing, China. Chinese Journal of Vector Biology and Control 2019; 30: 248–251. (in Chinese)

344. Peng PY. Species diversity and faunal taxonomy of gamasid mites from small mammals in Yunnan. PhD thesis: Guizhou University; 2019. (in Chinese)

345. Wang YJ, Yan DM, Li GC. Epidemiological characteristics of scrub typhus in Pinggu district of Beijing, China, in 2008–2018. Chin J Vector Biol & Control 2019; 30: 244–247. (in Chinese)

346. Yao LS. Serum C-reactive protein and procalcitonin values in acute Q fever, scrub typhus, and murine typhus. BMC Infect Dis 2020; 20.

347. Liu XH, Guo XG, Qian TJ. Current state of research on the gamasid mite *Eulaelaps stabularis*. Journal of Pathogen Biology 2020; 15: 111–114.
